# Tuberculosis prevalence after 4 years of population-wide systematic TB symptom screening and universal testing and treatment for HIV in the HPTN 071 (PopART) community-randomised trial in Zambia and South Africa: A cross-sectional survey (TREATS)

**DOI:** 10.1371/journal.pmed.1004278

**Published:** 2023-09-08

**Authors:** Eveline Klinkenberg, Sian Floyd, Kwame Shanaube, Linda Mureithi, Thomas Gachie, Petra de Haas, Barry Kosloff, Peter J. Dodd, Maria Ruperez, Chali Wapamesa, James Michael Burnett, Nico Kalisvaart, Nkatya Kasese, Redwaan Vermaak, Albertus Schaap, Sarah Fidler, Richard Hayes, Helen Ayles, on behalf of the TREATS study team

**Affiliations:** 1 London School of Hygiene & Tropical Medicine (LSHTM), London, United Kingdom; 2 Department of Global Health and Amsterdam Institute for Global Health and Development, Amsterdam University Medical Center, Amsterdam, the Netherlands; 3 KNCV Tuberculosis Foundation, Hague, the Netherlands; 4 Zambart, University of Zambia School of Public Health, Lusaka, Zambia; 5 Health Systems Trust, Cape Town, South Africa; 6 School of Health and Related Research, University of Sheffield, Sheffield, United Kingdom; 7 HIV Clinical Trials Unit, Imperial College London, London, United Kingdom

## Abstract

**Background:**

Tuberculosis (TB) prevalence remains persistently high in many settings, with new or expanded interventions required to achieve substantial reductions. The HIV Prevention Trials Network (HPTN) 071 (PopART) community-randomised trial randomised 14 communities to receive the “PopART” intervention during 2014 to 2017 (7 arm A and 7 arm B communities) and 7 communities to receive standard-of-care (arm C). The intervention was delivered door-to-door by community HIV care providers (CHiPs) and included universal HIV testing, facilitated linkage to HIV care at government health clinics, and systematic TB symptom screening. The Tuberculosis Reduction through Expanded Anti-retroviral Treatment and Screening (TREATS) study aimed to measure the impact of delivering the PopART intervention on TB outcomes, in communities with high HIV and TB prevalence.

**Methods and findings:**

The study population of the HPTN 071 (PopART) trial included individuals aged ≥15 years living in 21 urban and peri-urban communities in Zambia and South Africa, with a total population of approximately 1 million and an adult HIV prevalence of around 15% at the time of the trial. Two sputum samples for TB testing were provided to CHiPs by individuals who reported ≥1 TB suggestive symptom (a cough for ≥2 weeks, unintentional weight loss ≥1.5 kg in the last month, or current night sweats) or that a household member was currently on TB treatment. Antiretroviral therapy (ART) was offered universally at clinics in arm A and according to local guidelines in arms B and C. The TREATS study was conducted in the same 21 communities as the HPTN 071 (PopART) trial between 2017 and 2022, and TB prevalence was a co-primary endpoint of the TREATS study. The primary comparison was between the PopART intervention (arms A and B combined) and the standard-of-care (arm C). During 2019 to 2021, a TB prevalence survey was conducted among randomly selected individuals aged ≥15 years (approximately 1,750 per community in arms A and B, approximately 3,500 in arm C). Participants were screened on TB symptoms and chest X-ray, with diagnostic testing using Xpert-Ultra followed by culture for individuals who screened positive. Sputum eligibility was determined by the presence of a cough for ≥2 weeks, or ≥2 of 5 “TB suggestive” symptoms (cough, weight loss for ≥4 weeks, night sweats, chest pain, and fever for ≥2 weeks), or chest X-ray CAD4TBv5 score ≥50, or no available X-ray results. TB prevalence was compared between trial arms using standard methods for cluster-randomised trials, with adjustment for age, sex, and HIV status, and multiple imputation was used for missing data on prevalent TB. Among 83,092 individuals who were eligible for the survey, 49,556 (59.6%) participated, 8,083 (16.3%) screened positive, 90.8% (7,336/8,083) provided 2 sputum samples for Xpert-Ultra testing, and 308 (4.2%) required culture confirmation. Overall, estimated TB prevalence was 0.92% (457/49,556). The geometric means of 7 community-level prevalence estimates were 0.91%, 0.70%, and 0.69% in arms A, B, and C, respectively, with no evidence of a difference comparing arms A and B combined with arm C (adjusted prevalence ratio 1.14, 95% confidence interval, CI [0.67, 1.95], *p* = 0.60). TB prevalence was higher among people living with HIV than HIV–negative individuals, with an age-sex-community adjusted odds ratio of 2.29 [95% CI 1.54, 3.41] in Zambian communities and 1.61 [95% CI 1.13, 2.30] in South African communities. The primary limitations are that the study was powered to detect only large reductions in TB prevalence in the intervention arm compared with standard-of-care, and the between-community variation in TB prevalence was larger than anticipated.

**Conclusions:**

There was no evidence that the PopART intervention reduced TB prevalence. Systematic screening for TB that is based on symptom screening alone may not be sufficient to achieve a large reduction in TB prevalence over a period of several years. Including chest X-ray screening alongside TB symptom screening could substantially increase the sensitivity of systematic screening for TB.

**Trial registration:**

The TREATS study was registered with ClinicalTrials.gov Identifier: NCT03739736 on November 14, 2018. The HPTN 071 (PopART) trial was registered at ClinicalTrials.gov under number NCT01900977 on July 17, 2013.

## Abstract 2

In a population based cohort study, Eveline Klinkenberg and colleagues examine the impact of the PopART intervention on TB prevalence in Zambia and South Africa.

## Author summary

**Why was this study done?:**

- Tuberculosis (TB) prevalence remains persistently high in many settings, with new or expanded interventions required to achieve substantial reductions.
- The HIV Prevention Trials Network (HPTN) 071 Population Effects of Antiretroviral Therapy to Reduce HIV Transmission (PopART) community-randomised trial, conducted in 21 Zambian and South African communities (2013 to 2018), provided an opportunity to test whether the PopART intervention—universal testing and universal treatment for HIV combined with universal symptom screening for TB—could reduce TB prevalence.
- The Tuberculosis Reduction through Expanded Anti-retroviral Treatment and Screening (TREATS) study measured the impact of the PopART intervention on TB prevalence, comparing 14 communities that received the PopART intervention during 2014 to 2017 with 7 communities that received standard-of-care.

**What did the researchers do and find?:**

- We conducted a TB prevalence survey during 2019 to 2021, among around 50,000 randomly selected individuals aged ≥15 years in the 21 urban and peri-urban communities that were included in the HPTN 071 (PopART) trial.
- Overall, estimated TB prevalence was 0.92% (457/49,556). There was no evidence of a difference in TB prevalence between the PopART intervention and standard-of-care communities, with an adjusted prevalence ratio of 1.14, 95% confidence interval (CI) [0.67, 1.95].
- TB prevalence was around 2 times higher among HIV–positive than HIV–negative individuals, adjusted for age, sex, and community, and several times higher among men than women.

**What do these findings mean?:**

- This study found no evidence that the PopART intervention reduced TB prevalence, compared with standard-of-care.
- Systematic screening for TB that is based on symptom screening alone may not be sufficient to achieve a large reduction in TB prevalence over a period of a few years.
- Including chest X-ray screening alongside TB symptom screening could substantially increase the sensitivity of systematic screening for TB, and several years of delivering such a strategy might be able to achieve a considerable reduction in TB prevalence.

## Introduction

Tuberculosis (TB) overtook HIV as the leading infectious cause of death worldwide, prior to the Coronavirus Disease 2019 (COVID-19) pandemic, requiring a major policy shift if it is to be controlled in line with the global commitment to “end TB” [1]. One potential strategy, as outlined in WHO updated screening guidelines of 2021, is to increase systematic screening for TB in settings with high TB prevalence (>0.5%), with diagnostic testing offered to individuals who screen positive (for example on TB symptoms, chest X-ray, or belonging to a “high-risk” population such as household contacts or HIV–positive individuals) [2] or to the whole population [3]. This would have an immediate effect on TB prevalence, and by facilitating early diagnosis has the potential to reduce transmission of infection and later TB incidence and prevalence. Systematic screening for HIV–positive individuals has been recommended by WHO since 2011 [4]; in 2015, WHO guidelines [5] also recommended that antiretroviral therapy (ART) should be offered universally to HIV–positive individuals who know their HIV–positive status, with a reduction in TB incidence among such individuals being one of the expected health benefits.

Meanwhile, 4 community-randomised trials have shown that delivery of universal HIV testing, and universal ART for HIV–positive individuals (universal testing and treatment, UTT), achieves high ART coverage and viral suppression among HIV–positive individuals [6,7]. Two of the trials also found evidence that delivery of UTT reduces HIV incidence in the general adult population. It is possible that delivery of UTT also reduces TB incidence among HIV–positive individuals (through increased ART coverage), and following this it may indirectly reduce general-population TB incidence and prevalence [8].

The HIV Prevention Trials Network (HPTN) 071 (PopART) community-randomised trial was conducted during 2013 to 2018 in 21 communities in Zambia and the Western Cape Province of South Africa and was designed to measure the impact of a combination HIV prevention package (the PopART intervention) on HIV incidence [9]. The study built on previous TB and HIV research that was conducted in many of the same communities, including the ZAMSTAR trial during 2004 to 2011 [10,11]. The PopART package of interventions was centred on universal HIV testing and support for linkage to HIV care, with or without universal ART, and included systematic screening for TB based on TB symptoms. During 2015 to 2018 HIV incidence was on average around 20% lower across the 14 communities that were randomised to receive the PopART intervention than in the 7 communities that were randomised to the standard-of-care arm [7].

To contribute to the evidence base on how to reduce TB burden in HIV-endemic settings, the TREATS (Tuberculosis Reduction through Expanded Anti-retroviral Treatment and Screening) study was designed to measure the population-level impact of the PopART intervention on TB prevalence, transmission, and TB notifications. We report here on the impact of the PopART intervention on TB prevalence; this was measured during 2019 to 2021, 2 to 4 years after the last year of the PopART intervention delivery.

## Methods

### Ethics statement

The TREATS study was approved by the research ethics committees of the London School of Hygiene & Tropical Medicine, the University of Zambia (UNZABREC), and Pharma-Ethics (Pty) in South Africa. Individuals gave written informed consent to survey participation, and for individuals aged 15 to 17 years, assent and parental consent were obtained.

The TREATS study was registered with ClinicalTrials.gov Identifier: NCT03739736 on November 14, 2018. The HPTN 071 (PopART) trial was registered at ClinicalTrials.gov under number NCT01900977 on July 17, 2013.

### HPTN 071 (PopART) community-randomised trial design, PopART intervention, and TREATS study design

The HPTN 071 (PopART) community-randomised trial was conducted during 2013 to 2018. All communities were urban or peri-urban, 12 were in Zambia and 9 were in the Western Cape Province of South Africa, with a total population of around 1 million individuals and estimated adult HIV prevalence of around 15%. In the HPTN 071 (PopART) trial, the 21 communities were matched into 7 triplets based on geographical area and estimated HIV prevalence [9]. Within each triplet, 1 community was randomised to arm A, 1 to arm B, and 1 to arm C, with restricted randomisation used to ensure overall balance among the 3 trial arms in terms of HIV prevalence, ART coverage, and population size. The randomisation of communities to trial arms was done in a public randomisation ceremony in 2013 [9]. The 3 trial arms were arm A (PopART intervention, with UTT and also systematic screening for TB based on TB symptoms), arm B (as arm A, except ART according to local guidelines [universal ART from mid-2016]), or arm C (standard-of-care).

The PopART intervention was delivered by community HIV care providers (CHiPs) for 4 years (2014 to 2017), primarily through a door-to-door approach, with CHiPs aiming to reach all households and all community members who were aged ≥15 years approximately annually. HIV testing was offered to all who participated in the intervention, apart from individuals who self-reported they were HIV–positive. Systematic screening for TB was done for everyone who reported they were not currently on TB treatment, based on TB symptoms. Two sputum samples were requested from individuals who reported any among a cough for ≥2 weeks, unintentional weight loss ≥1.5 kg in the last month, current night sweats, or that a member of their household was currently on TB treatment. CHiPs took the sputum samples to the health clinic, where TB diagnostic testing followed national guidelines; in South African communities Xpert-MTB/RIF was used, and in Zambian communities Xpert-MTB/RIF was used for people living with HIV (PLWH) and smear microscopy was used for all other individuals. CHiPs made at least 1 follow-up visit to individuals who had TB symptoms or were a household contact of a TB patient at the time of the annual visit, to provide TB diagnostic test results and support for starting TB treatment (if required). They also made regular follow-up visits to individuals who were known to be HIV–positive, to support linkage to HIV care and retention on ART. Alongside this, routine TB service provision at the health clinic was strengthened in various ways, in collaboration with each country’s Department of Health or Ministry of Health. This strengthening included additional provision of machines for Xpert-MTB/RIF testing, additional Xpert-MTB/RIF cartridges and reagents, support to staff doing laboratory testing, and support to strengthen recordkeeping for TB registers. TB preventive therapy was recommended for PLWH and individuals aged <5 years old who were a household contact of a TB patient, according to national guidelines, but uptake of such therapy was low during the period of the PopART intervention delivery.

The TREATS study was conducted in the same 21 communities as the HPTN 071 (PopART) trial and was designed as a follow-up study to the HPTN 071 (PopART) trial. The TREATS study was designed to measure the impact of the PopART intervention on TB outcomes, including TB prevalence, and was conducted during 2017 to 2022. The primary comparison of TB prevalence was between arms A and B combined (PopART intervention) versus arm C (standard-of-care). Arms A and B were combined because the TB screening and universal HIV testing components of the PopART intervention were the same throughout the HPTN 071 (PopART) trial, and universal ART was offered in arm B from mid-2016 (when universal ART became national guidelines).

### TREATS TB prevalence survey design

#### Sample size and survey timing

The TREATS TB prevalence survey was conducted among a random sample of individuals aged ≥15 years during 2019 to 2021. The target sample size was 3,000 to 4,000 participants in each arm C community and 1,500 to 2,000 participants in each arm A and arm B community, with a total targeted sample size of around 50,000 participants. With an average of 1,750 participants in each arm A and arm B community, an average of 3,500 participants in each arm C community, an assumed coefficient of variation k of 0.25, and an assumed 50% reduction in TB prevalence in arms A and B compared with arm C, study power was 91% if average arm C TB prevalence was 1%, and 89% if average arm C TB prevalence was 0.8%. The corresponding figures for study power to show a 45% reduction in TB prevalence were 84% and 81%, respectively. The assumption that TB prevalence could be reduced by around 50% after several years of delivery of the PopART intervention was informed by mathematical modelling projections that were done before the start of the TREATS study (see S1 Protocol for more details).

Community sensitisation was conducted prior to the start of prevalence survey activities, including distribution of information leaflets, door-to-door information provision, and involvement of the Community Advisory Board (CAB).

#### Field procedures

Within each community, random sampling was structured according to geographically defined blocks of around 200 households. For every randomly selected block, all households were visited by a research assistant. If an adult household member was found at home, permission was sought to enumerate (list) all household members. In enumerated households, an individual was eligible to participate if they were a community resident aged ≥15 years. Eligible individuals were given barcoded invitation cards and invited to attend a mobile field site (MFS) that was located within the community as close to the sampled block as possible. A OneStopTB Platform (a truck, containing a digital X-ray and mobile laboratory fitted with an Xpert instrument) was stationed at the MFS, and several tents (“stations”) were set up to provide spaces to administer questionnaires and collect samples.

All individuals who attended the MFS and consented to participate in the survey followed a defined order of procedures. First, questionnaire information including sociodemographic and socioeconomic characteristics, TB symptoms, previous history of TB treatment, and behavioural risk factors for TB (smoking, drinking alcohol, occupational exposure), was collected. Subsequently, individuals were invited to have a digital chest X-ray taken, with images read using computer-aided detection software (CAD4TB, version 5.0, Delft Imaging, the Netherlands) that provided a score between 0% and 100% representing the probability that an individual has TB. After this, self-reported information on previous HIV testing and current and past ART use was collected, including whether an individual had ever tested for HIV, the date and result of their last test, and whether they had tested for HIV in the previous 12 months. Following this, HIV testing was offered to all individuals who did not self-report they were HIV–positive. All individuals who tested HIV–positive were referred to HIV services following counselling. An individual was considered to have participated in the survey if they were screened for TB symptoms and attended the X-ray station.

#### Eligibility to provide 2 sputum samples (S1 and S2) for Xpert-Ultra testing

“Sputum-eligible” individuals were identified based on TB screening using symptoms and chest X-ray. An individual was sputum-eligible based on TB symptoms if they had a cough for ≥2 weeks or if they had 2 or more among 5 “TB suggestive” symptoms (cough of any duration, unexpected weight loss for ≥4 weeks, night sweats for ≥2 weeks, chest pains for ≥2 weeks, fever for ≥2 weeks). An individual was sputum-eligible based on chest X-ray if their X-ray CAD4TBv5 score was ≥50, or they did not have an X-ray done, or the X-ray was done but the X-ray score was not captured (S1 Fig). All sputum-eligible individuals were requested to provide 2 “on-the-spot” sputum samples (S1 and S2, taken ≥30 min apart), for Xpert-Ultra testing within the next 24 h; this testing was done in the OneStopTB truck, and followed the manufacturer’s standard operating procedures [12]. Sputum-eligible individuals were asked to return the following day to receive their Xpert-Ultra test results, and during this “day 2” visit they were also reviewed by a medical officer who made decisions on referral to TB or other care based on their screening and test results. There was active tracing of individuals who did not return for the “day 2” follow-up, with a focus on tracing those with positive Xpert-Ultra test results.

Xpert-Ultra results for sputum samples S1 and S2 were classified using the test read-out as *M*. *tuberculosis* (MTB) not detected, MTB-trace-detected, MTB detected very low, low, medium, or high; or as non-determinate (invalid, error, or no result). If the Xpert-Ultra test result was “non-determinate,” the test was repeated up to 2 times, either on the original sample or (if needed) on a new sample.

#### Eligibility to provide sputum samples (S3, or S3 and S4) for culture testing

Collection of sputum samples for culture testing, and the number of samples requested, differed between the first 4 study communities (in which an “intensive diagnostic phase” (IDP) was conducted) and the remaining 17 “non-IDP” communities.

In the 4 IDP communities, all sputum-eligible individuals who returned on “day 2” were requested to provide 1 sputum sample (S3) for culture testing. Comparison of the Xpert-Ultra and culture results in these IDP communities was used to define a subgroup of sputum-eligible individuals who would be “culture eligible” in the 17 non-IDP communities [13].

In the 17 non-IDP communities, sputum-eligible participants who had valid Xpert-Ultra test results from both of the S1 and S2 sputum samples were classified into one of 3 categories (S2 and S3 Figs). First, if both of the S1 and S2 Xpert results were “*M*. *tuberculosis* (MTB) not detected,” then the individual was not culture-eligible and was considered not to have prevalent TB. Second, if both of the S1 and S2 Xpert results were “MTB detected” with a grade of very low or above, and at least one of them was graded as low, medium, or high, then the individual was not culture-eligible and was considered to have prevalent TB. Third, individuals with other combinations of S1 and S2 Xpert test results (for example, both samples “MTB detected” but with a trace-positive or very low grade result, or 1 sample “MTB not detected” and 1 sample “MTB detected”) were “culture-eligible.” Culture-eligible individuals were requested to provide 2 additional sputum samples (S3 and S4) for culture testing, and these culture test results were used to determine their prevalent TB status.

#### Culture testing of sputum samples

All sputum samples that were collected for culture testing were batched and kept in a refrigerator until being transported on the same day to the central laboratory in a cooler box. In Zambia, samples were taken to the Zambart central laboratory in Lusaka, and in South Africa to the National Health Laboratory Service (NHLS) laboratory in Greenpoint, Cape Town. At the culture laboratory, culture testing followed standard procedures that have been described previously [13] and further details are given also in S1 Text and S4 Fig. In short, each sputum sample was inoculated onto 2 mycobacteria growth indicator tubes (MGITs) and incubated for 42 days or until growth was observed. Diagnostic testing was done on growth-positive tubes (S4 Fig, step 2).

The culture result for each sputum sample was defined based on the combination of the culture results from the 2 tubes (S4 Fig, steps 3 to 5). A sample was classified as culture-positive for *M*. *tuberculosis* if ≥1 tube result was positive for *M*. *tuberculosis*. Among samples that were not culture-positive for *M*. *tuberculosis*, samples were classified as culture-negative if ≥1 tube was culture-negative or ≥1 tube was positive for non-tuberculous-mycobacteria. The result was classified as contaminated if both tubes were contaminated and as non-interpretable if either both tubes were non-interpretable, or one was non-interpretable and one was contaminated. We defined a sputum sample culture result as valid if it was culture-positive for *M*. *tuberculosis* or if it was culture-negative, and also from a batch where the positive control grew and the negative control did not; other results were classified as missing.

The culture result for each individual was defined based on the combination of the culture results from the S3 and S4 samples. An individual had a valid culture result if one or both of the S3 and S4 sample results were valid and was classified as having prevalent TB if ≥1 sample was culture-positive for *M*. *tuberculosis*, and as not having prevalent TB if neither sample was culture-positive for *M*. *tuberculosis* and ≥1 sample was culture-negative, with other individuals classified as having missing data on their culture status (S4 Fig, step 5).

#### Definition of the primary outcome of prevalent TB

Sputum-eligible participants who had valid Xpert-Ultra results from both S1 and S2 and were not culture-eligible (according to the non-IDP community definition) were classified as prevalent TB (yes or no) based solely on their Xpert-Ultra results (see above).

Sputum-eligible participants who were culture-eligible (according to the non-IDP community definition) were classified as prevalent TB (yes or no) based solely on their culture results. Those who were culture-positive for *M*. *tuberculosis* were considered to have prevalent TB, those who were culture-negative for *M*. *tuberculosis* were considered not to have prevalent TB, and those with no valid culture result were classified as having missing data on prevalent TB.

Sputum-eligible participants with missing Xpert-Ultra data on one or both of the S1 and S2 sputum samples (a test result that was non-determinate or a sample that was not provided) were classified as having missing data on prevalent TB. It was assumed that participants who were not eligible to provide sputum samples did not have prevalent TB.

#### Data capture and statistical analysis

Data were captured digitally into an electronic data management system (DMS) specifically designed for the TREATS TB prevalence survey while culture data were captured using each laboratory’s existing laboratory information management system (LIMS).

For sputum-eligible participants whose prevalent TB status could not be determined due to missing Xpert-Ultra or culture test results, imputation of the missing data was done (further details in S2 Text). The first step was to impute missing data on prevalent TB (yes or no) among culture-eligible individuals with missing data on culture results, and the second step was to impute missing data on prevalent TB (yes or no) for the sputum-eligible individuals who had missing Xpert-Ultra results. A total of 300 imputed datasets were created (30 times 10; 30 imputed datasets for step 1 and for each of these 30 datasets, 10 imputations were done for step 2).

TB prevalence was then compared among trial arms using a standard “two-stage” approach for the analysis of cluster-randomised trials with <15 clusters per trial arm [14], but accounting for the missing data by applying this two-stage approach to each of the 300 imputed datasets. In Stage 1, a logistic regression model was fitted with prevalent TB (yes or no) as the outcome and study triplet, age group, sex, and HIV status as explanatory variables; one regression model was fitted for all Zambian communities (4 study triplets and 12 communities, with study triplet as a “fixed” effect) and one regression model was fitted for all South African communities (3 triplets and 9 communities, with study triplet as a “fixed” effect). The adjustment for study triplet, age group, sex, and HIV status was prespecified in the statistical analysis plan (see Supporting information file S1 SAP). The country-specific logistic regression models were then used to predict the probability that an individual had prevalent TB, under the null hypothesis of no intervention effect. These predicted probabilities were then summed across all individuals in the same community, as an estimate of the expected number (E) of individuals in the community with prevalent TB under the null hypothesis. In subgroup analysis, the fitting of the Stage 1 model and the summation of observed and expected values were done with restriction to individuals belonging to the subgroup—for example with restriction to males, or with restriction to HIV–negative individuals. In Stage 2, for each community the ratio of the observed (including individuals imputed to have prevalent TB) number of individuals with prevalent TB (O) to the expected number (E) was calculated. A linear regression model with log(O/E) as the outcome and triplet and trial arm as explanatory variables was then fitted, to obtain estimates of the TB prevalence ratios (and corresponding 95% confidence intervals (CIs)) comparing arms A and B combined with arm C (primary analysis) as well as comparing each of arms A and B separately with arm C. Lastly, the prevalence ratio estimates from each of the imputed datasets were averaged (using a geometric mean) to obtain an overall summary estimate, and Rubin’s rules were used to obtain 95% CIs that accounted for the imputation of missing data. Subgroup analysis for males and females, older and younger individuals, HIV–positive and HIV–negative individuals, and individuals who had been resident in the study communities for ≥5 years, was prespecified. Analyses were conducted in Stata 17.

## Results

### Enumeration and participation

**Fig 1:**
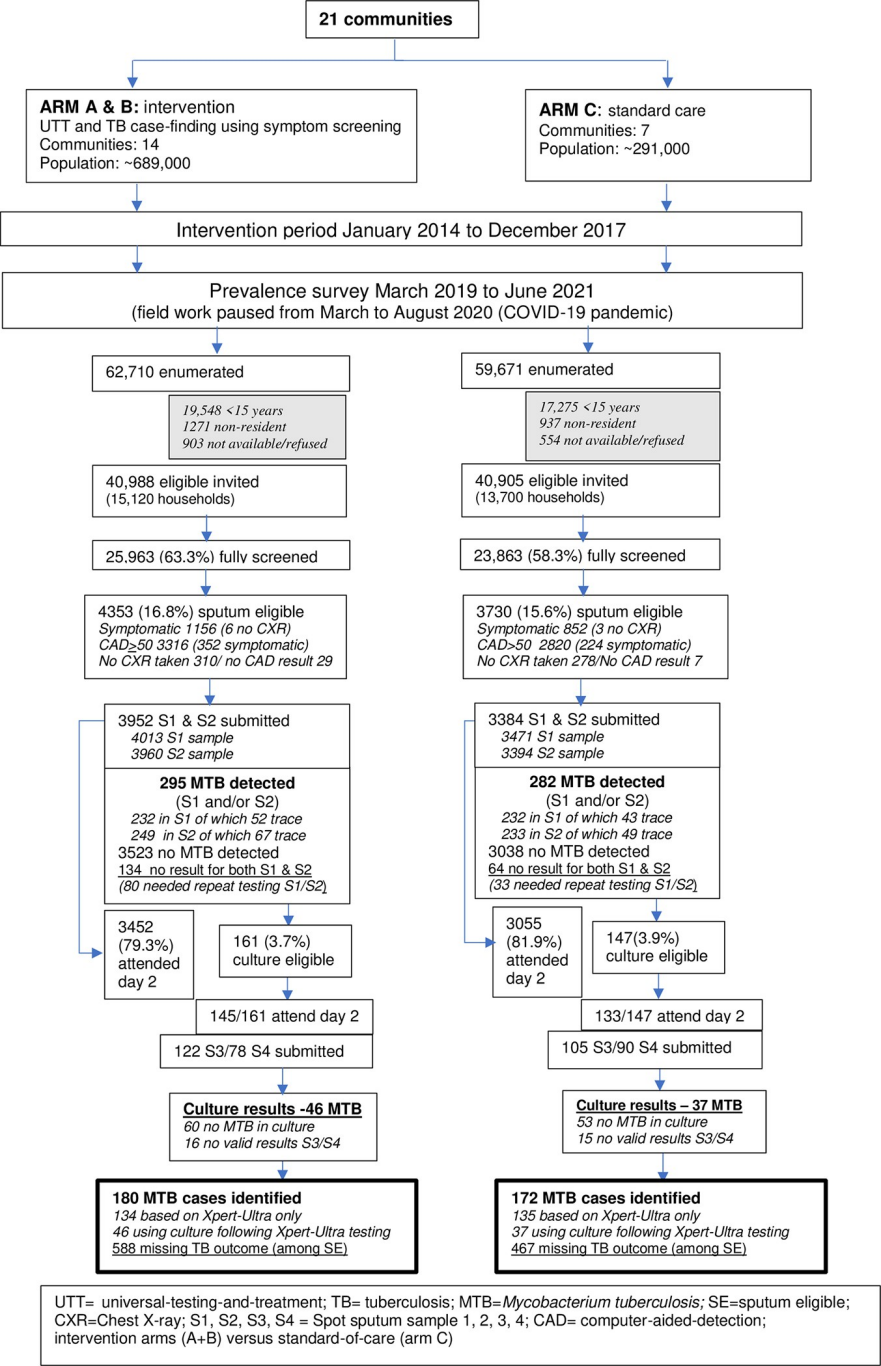
Flow diagram by trial arm of participant enrolment, sample collection, and MTB detection. MTB, *M*. *tuberculosis*; TB, tuberculosis; UTT, universal testing and treatment.

A total of 122,381 household members (all ages) were enumerated, 89,850 in Zambia and 32,531 in South Africa, 83,092 were eligible to participate, and 49,556 (59.6%) participated (Figs 1 and S5). The participation rate varied slightly by trial arm (62.2% in arm A, 60.9% in arm B, 57.7% in arm C, S6 Fig), was higher in South African than Zambian communities (69.3% versus 55.0%, S5 Fig), and higher in females than males (66.1% versus 51.8%). The proportion of participants who were eligible to provide sputum samples based on the TB symptoms they reported was similar in South African (4.4%) and Zambian (3.8%) communities and varied slightly by trial arm (4.7%, 4.3%, and 3.6% in arms A, B, and C, respectively). The proportion of participants who were eligible to provide sputum samples based on an abnormal X-ray was higher in South African (16.8%) than Zambian (11.4%) communities, and similar by trial arm (13.4%, 13.4%, and 13.0% in arms A, B, and C, respectively). Overall, 8,083 (16.3%) participants were sputum-eligible and this was similar among trial arms (16.7%, 17.3%, and 15.6% in arms A, B, and C, respectively, Figs 1 and S6), and 80.5% (6,508/8,083) returned for the day 2 visit (78.9% in arm A, 79.8% in arm B, and 81.9% in arm C). Day 2 attendance differed by country, being 77% in Zambian communities and 84% for South African communities (S5 Fig).

### Characteristics of participants

The age-sex profile of participants was similar by trial arm (Table 1). A slightly higher proportion of participants had been resident in the community for ≥5 years in arms A and B than in arm C (73.7% in arms A and B combined versus 69.9% in arm C). Education level patterns were similar by trial arm, though in arm C a slightly higher proportion had completed secondary school grade or higher education (72.2% in arms A and B combined versus 77.3% in arm C).

**Table 1:** Characteristics (absolute numbers and relevant proportions) of the study population by arm and overall combined.

	Arm A		Arm B		Arm C		Totals	
	***n***	**%**	***n***	**%**	***n***	**%**	***n***	**%**
**Number seen at site (fully screened)**	**13,906**		**11,787**		**23,863**		**49,556**	
**Number female**	8,552	61.5%	7,282	61.8%	14,252	59.7%	30,086	60.7%
**Age group**								
15–24 years	5,056	36.4%	4,417	37.5%	9,114	38.2%	18,587	37.5%
25–34 years	3,639	26.2%	2,930	24.9%	6,073	25.4%	12,642	25.5%
35–44 years	2,357	16.9%	1,995	16.9%	3,866	16.2%	8,218	16.6%
45–54 years	1,418	10.2%	1,205	10.2%	2,288	9.6%	4,911	9.9%
55+ years	1,436	10.3%	1,240	10.5%	2,522	10.6%	5,198	10.5%
**Length of residence**								
<1 years	685	4.9%	570	4.8%	1,583	6.6%	2,838	5.7%
1–5 years	2,984	21.5%	2,528	21.4%	5,594	23.4%	11,106	22.4%
>5–10 years	3,226	23.2%	2,909	24.7%	4,900	20.5%	11,035	22.3%
>10 years	7,011	50.4%	5,780	49.0%	11,786	49.4%	24,577	49.6%
**Education level**								
None	483	3.5%	502	4.3%	626	2.6%	1,611	3.3%
Primary school	3,412	24.5%	2,750	23.3%	4,791	20.1%	10,953	22.1%
Secondary school	9,405	67.6%	8,060	68.4%	17,276	72.4%	34,741	70.1%
Higher education	606	4.4%	475	4.0%	1,170	4.9%	2,251	4.5%
**History TB treatment**								
Previous TB	1,304	9.4%	976	8.3%	2,076	8.7%	4,356	8.8%
Current TB	128	0.9%	76	0.6%	91	0.4%	295	0.6%
HIV status*								
Negative	10,688	76.9%	8,916	75.6%	17,391	72.9%	36,995	74.7%
Positive	2,322	16.7%	1,847	15.7%	3,808	16.0%	7,977	16.1%
Unknown	896	6.4%	1,024	8.7%	2,664	11.2%	4,584	9.3%
**HIV and ART status**								
Negative	10,688	76.9%	8,916	75.6%	17,391	72.9%	36,995	74.7%
Self-reported positive, on ART	1,962	14.1%	1,476	12.5%	3,160	13.2%	6,598	13.3%
Self-reported positive, not on ART	151	1.1%	133	1.1%	285	1.2%	569	1.1%
Tested HIV–positive in survey	209	1.5%	238	2.0%	363	1.5%	810	1.6%
Unknown	896	6.4%	1,024	8.7%	2,664	11.2%	4,584	9.3%

* After completing TREATS study procedures so including testing done as part of TREATS.

ART, antiretroviral therapy; HIV, human immunodeficiency virus; TB, tuberculosis; TREATS, Tuberculosis Reduction through Expanded Anti-retroviral Treatment and Screening.

Overall, 4,356 (8.8%) of participants self-reported they had previously been treated for TB and 295 (0.4%) self-reported being on TB treatment on the day of the survey. This was higher in South Africa (14.2% and 1.1%, respectively) than in Zambia (5.5% and 0.3%, respectively) (S1 Table). Self-reported previous TB treatment and current TB treatment were slightly higher in arm A (9.4% and 0.9%, respectively) than in arm B (8.3% and 0.6%) and arm C (8.7% and 0.4%).

Overall, 96% (47,581/49,556) of participants completed the HIV interview, and this did not differ by trial arm (S2 Table). Overall, 42,008 (88.3%) reported they had previously been tested for HIV, and this was slightly lower in arm C (85.9%) than in arm A (91.4%) and arm B (89.5%). Overall, 68.2% either self-reported they were HIV–positive or that they had tested HIV–negative in the previous 12 months, and this was slightly higher in arm A (71.4%) than in arm B (67.0%) and arm C (67.0%). Overall, 14.5% (7,181/49,556) of participants self-reported they were HIV–positive, and among them 92.1% (6,613/7,181) self-reported they were currently on ART, and these figures were similar by trial arm.

Among the 40,409 participants who completed the HIV interview and did not self-report being HIV–positive, 73.9% (*n* = 29,882) accepted an offer of HIV testing. The proportion who accepted this offer was slightly lower in arm C (70.2%) than in arm A (74.6%) and arm B (81.0%), and 2.7% (*n* = 803) tested HIV–positive and this was similar by trial arm (S2 Table).

Following survey participation, 16.1% of participants were known to be HIV–positive; 13.3% of participants self-reported they were HIV–positive and taking ART, 1.1% self-reported they were HIV–positive and not taking ART, and 1.6% did not self-report as HIV–positive but tested HIV–positive in the survey. Seventy-five percent of participants tested HIV–negative as part of the survey or self-reported an HIV–negative test in the previous 12 months, and the HIV status of the remaining 9.3% was considered unknown. Figures were similar by trial arm, apart from a slightly higher percentage being of unknown HIV status in arm C (Table 1 and S2 Table).

Supporting information S5 and S6 Figs and S1–S3 Tables provide further details by country and arm.

### Provision of sputum samples, valid Xpert-Ultra results, culture eligibility, and valid culture results

Around 93% of sputum-eligible individuals (7,502/8,083) produced at least 1 sputum sample for Xpert-Ultra testing, 91% provided 2 samples (S1 and S2), and 88% (7,138/8,083) had valid Xpert-Ultra results from both sputum samples, among whom 4.3% (308/7,138) were culture-eligible (Fig 1). The proportions were similar by trial arm. Among the 308 culture-eligible individuals, 91% (279/308) attended the day 2 visit, for 227/279 (81%) an S3 sample was provided and for 168 (60%) an S4 sample was provided; 198 (64%) of culture-eligible individuals had valid culture results and this proportion was higher in arm A than in arms B and C (76%, 57%, and 61% in arms A, B, and C, respectively) (Figs 1 and S6). Overall, 352 participants were diagnosed with prevalent TB, 129 in Zambia and 223 in South Africa. Twenty-eight percent of individuals with prevalent TB screened positive on TB symptoms (42% in Zambian and 20% in South African communities, S3 Table).

### TB prevalence estimates and comparison among trial arms

Overall, the estimated proportion of survey participants with prevalent TB (after imputing missing data) was 0.92% (457/49,556), lower in Zambian (0.51%, 158/30,908) than South African communities (1.60%, 299/18,648). TB prevalence estimates varied substantially among communities (Table 2), in Zambia ranging from 0.13% in the arm B and arm C study communities in triplet 4 to 0.84% in the arm A community in triplet 2 and the arm B community in triplet 3, and in South Africa ranging from 1.11% in the arm A community in triplet 5 to 2.33% in the arm C community in triplet 7. The coefficient of between-community variation k was approximately 0.3, after accounting for between-triplet and between-arm variation.

**Table 2:** TB prevalence by trial arm, overall and stratified by sex, age group, and HIV status.

		Arm A		Arm B		Arm A+B		Arm C	
**Overall**	Triplet	*n*/*N*^1^	%^2^	*n*/*N*^1^	%^2^	*n*/*N*^1^	%^2^	*n*/*N*^1^	%^2^
	1	11.4/1,971	0.58	12.9/1,905	0.67	24.3/3,876	0.62	10.4/3,494	0.30
	2	17.5/2,088	0.84	4.9/2,122	0.23	22.4/4,210	0.52	22.6/4,064	0.56
	3	20.7/2,887	0.72	18.7/2,235	0.84	39.4/5,122	0.78	22.1/3,813	0.58
	4	10.9/1,609	0.68	2.1/1,548	0.13	13.0/3,157	0.41	4.2/3,172	0.13
	5	22.7/2,049	1.11	24.5/1,804	1.36	47.2/3,853	1.25	51.9/4,022	1.29
	6	20.7/1,772	1.17	15.5/693	2.24	36.2/2,465	1.46	61.6/3,042	2.03
	7	25.5/1,530	1.66	23.9/1,480	1.62	49.4/3,010	1.63	52.7/2,256	2.33
	Overall (triplets 1–7 combined)	129.4/13,906	0.93	102.4/11,787	0.87	231.8/25,693	0.90	225.5/23,863	0.94
	TB prevalence, geometric mean^3^		**0.91**		**0.70**		**0.80**		**0.69**
	Unadjusted PR, 95% CI, *p*-value	1.31	[0.70, 2.45] *p* = 0.36	1.01	[0.54, 1.90] *p* = 0.97	1.15	[0.67, 1.98] *p* = 0.58	referent	
	aPR^4^, 95% CI, *p*-value	**1.29**	**[0.69, 2.39] *p* = 0.38**	**1.01**	**[0.54, 1.88] *p* = 0.97**	**1.14**	**[0.67, 1.95] *p* = 0.60**	referent	
**Males**	Overall (triplets 1–7 combined)	93.7/5,354	1.75	71.5/4,505	1.59	165.2/9,859	1.68	139.0/9,611	1.45
	TB prevalence, geometric mean		1.70		1.25		**1.46**		**1.07**
	aPR, 95% CI, *p*-value	1.53	[0.79, 2.95] *p* = 0.18	1.12	[0.58, 2.18] *p* = 0.70	**1.31**	**[0.74, 2.32] *p* = 0.32**	referent	
**Females**	Overall (triplets 1–7 combined)	35.7/8,552	0.42	30.9/7,282	0.42	66.6/15,834	0.42	86.5/14,252	0.61
	TB prevalence, geometric mean		0.39		0.33		**0.36**		**0.42**
	aPR, 95% CI, *p*-value	0.90	[0.40, 2.04] *p* = 0.78	0.80	[0.35, 1.80] *p* = 0.55	**0.85**	**[0.42, 1.71] *p* = 0.61**	referent	
**Age <30 years**	Overall (triplets 1–7 combined)	45.0/7,094	0.63	28.4/6,095	0.47	73.4/13,189	0.56	70.2/12,517	0.56
	TB prevalence, geometric mean		0.60		0.18		**0.33**		**0.36**
	aPR, 95% CI, *p*-value	1.73	[0.66, 4.54] *p* = 0.23	0.86	[0.32, 2.32] *p* = 0.74	**1.22**	**[0.53, 2.84] *p* = 0.60**	referent	
**Age ≥30 years**	Overall (triplets 1–7 combined)	84.4/6,812	1.24	74.0/5,692	1.30	158.4/12,504	1.27	155.3/11,346	1.37
	TB prevalence, geometric mean		1.21		1.09		**1.15**		**1.04**
	aPR, 95% CI, *p*-value	1.16	[0.68, 1.98] *p* = 0.56	1.05	[0.62, 1.79] *p* = 0.84	**1.10**	**[0.69, 1.75] *p* = 0.65**	referent	
**HIV–negative**	Overall (triplets 1–7 combined)	81.7/10,688	0.76	64.9/8,916	0.73	146.6/19,604	0.75	148.0/17,391	0.85
	TB prevalence, geometric mean		0.73		0.57		**0.64**		**0.59**
	aPR, 95% CI, *p*-value	1.25	[0.65, 2.42] *p* = 0.47	0.98	[0.51, 1.91] *p* = 0.96	**1.11**	**[0.62, 1.97] *p* = 0.70**	referent	
**HIV–positive**	Overall (triplets 1–7 combined)	33.2/2,322	1.43	22.7/1,847	1.23	55.9/4,169	1.34	53.7/3,808	1.41
	TB prevalence, geometric mean		1.28		1.00		**1.13**		**1.13**
	aPR, 95% CI, *p*-value	1.16	[0.46, 2.89] *p* = 0.72	0.97	[0.38, 2.46] *p* = 0.94	**1.06**	**[0.48, 2.35] *p* = 0.87**	referent	

^1^ Number of individuals with prevalent TB/total participants. Number of individuals with prevalent TB is calculated after multiple imputation of missing data on prevalent TB status and is an average across 300 imputed datasets—hence not a whole number.

^2^ Percentage with prevalent TB.

^3^ Geometric mean of TB prevalence across 7 communities.

^4^ Adjusted triplet, sex, age group, HIV status.

aPR, adjusted PR; CI, confidence interval; HIV, human immunodeficiency virus; PR, prevalence ratio; TB, tuberculosis.

Across the communities in arms A and B, the geometric mean of the 14 community-level TB prevalences was 0.80%, with figures of 0.91% for arm A and 0.70% for arm B (Table 2). Across the arm C communities, the geometric mean of the 7 community-level TB prevalence values was 0.69% (Table 2).

The adjusted prevalence ratio comparing the combined intervention arm (arms A and B) versus the standard-of-care arm (arm C) was 1.14 (95% CI 0.67, 1.95) (Table 2), with no evidence of a difference between the trial arms (*p* = 0.60). There was also no evidence of a difference in TB prevalence between arm A and arm C communities, or between arm B and arm C communities.

TB prevalence was higher in males than females (for example, the geometric mean of the 14 community-level TB prevalence values in arms A and B combined was 1.46% among males compared with 0.36% among females), among older (≥30 years) compared with younger (<30 years) individuals (geometric means of 1.15% and 0.33%, respectively, in arms A and B combined) and among HIV–positive compared with HIV–negative individuals (geometric means of 1.13% and 0.64%, respectively, in arms A and B combined) (Table 2). Across these subgroups, there was no evidence that TB prevalence was different in arms A and B combined than in Arm C, for example among males the adjusted prevalence ratio was 1.31 [95% CI 0.74, 2.32] (Table 2). There was also no evidence of a difference between arms A and B combined compared with arm C when analysis was restricted to individuals who had been resident in the study communities for ≥5 years (adjusted prevalence ratio 1.26, 95% CI 0.68, 2.34).

### TB prevalence estimates, by country and HIV status

TB prevalence was higher among HIV–positive than HIV–negative individuals across almost all combinations of country, sex, and age group (Table 3). In Zambian communities, the age-adjusted odds ratios comparing HIV–positive to HIV–negative individuals were 1.80 [95% CI 1.10, 2.94] among males and 3.75 [95% CI 1.84, 7.66] among females, with weak evidence that the odds ratios differed between males and females (*p* = 0.09). In South African communities, the age-adjusted odds ratios were 1.40 [95% CI 0.85, 2.29] among males and 1.93 [95% CI 1.14, 3.25] among females, with no evidence that the odds ratios differed between males and females (*p* = 0.45). Overall, combining the data on males and females, the age-sex-triplet adjusted odds ratio comparing HIV–positive to HIV–negative individuals was 2.29 [95% CI 1.54, 3.41] in Zambian communities and 1.61 [95% CI 1.13, 2.30] in South African communities. The adjusted odds ratios, comparing HIV–positive with HIV–negative individuals, were similar when restricting HIV–positive individuals to those who self-reported they were HIV–positive and taking ART (S4 Table).

**Table 3:** TB prevalence by country and HIV status, overall and stratified by sex and age group.

	HIV–negative			HIV–positive^1^			Unadjusted OR, comparing HIV–positive with HIV–negative individuals	Adjusted OR^2^, 95% CI	
**Zambia**	n^3^	N^4^	%^5^	n^3^	N^4^	%^5^	OR	OR	95% CI
Male, age <30	28.0	5,886	**0.48**	1.7	162	**1.05**	2.04	2.06	[0.32, 13.42]
Male, age 30–39	18.7	1,590	**1.17**	12.8	339	**3.78**	3.31	3.44	[1.62, 7.30]
Male, age 40–49	20.0	877	**2.28**	12.6	447	**2.81**	1.24	1.24	[0.59, 2.61]
Male, age ≥50	9.4	1,063	**0.89**	2.1	333	**0.64**	0.72	0.77	[0.17, 3.59]
Male, all ages	**76.1**	9,416	**0.81**	**29.2**	1,281	**2.28**	2.86	**1.80**	[1.10, 2.94]
									*P* = 0.19^6^
Female, age <30	9.6	8,857	**0.11**	3.4	842	**0.41**	3.67	3.74	[0.91, 15.37]
Female, age 30–39	1.8	2,579	**0.07**	9.6	1,294	**0.74**	11.45	11.71	[1.72, 79.90]
Female, age 40–49	1.8	1,163	**0.16**	6.4	1,042	**0.62**	4.13	4.33	[0.66, 28.47]
Female, age ≥50	6.8	1,617	**0.42**	2.5	509	**0.49**	1.15	1.20	[0.25, 5.73]
Female, all ages	**20.0**	14,216	**0.14**	**22.0**	3,687	**0.60**	4.25	**3.75**	[1.84, 7.66]
									*P* = 0.34^6^
Males and females, all ages	**96.1**	**23,632**	**0.41**	**51.2**	**4,968**	**1.03**	2.55	**2.29**	**[1.54, 3.41]**
**South Africa**									
Male, age <30	44.8	3,114	**1.44**	2.6	92	**2.88**	1.99	1.97	[0.51, 7.64]
Male, age 30–39	28.5	1,181	**2.41**	6.4	222	**2.88**	1.18	1.26	[0.48, 3.31]
Male, age 40–49	31.1	675	**4.60**	8.7	203	**4.28**	0.92	1.02	[0.43, 2.39]
Male, age ≥50	31.1	944	**3.29**	7.8	141	**5.56**	1.72	1.98	[0.82, 4.76]
Male, all ages	**135.5**	5,914	**2.29**	**25.6**	658	**3.89**	1.72	**1.40**	[0.85, 2.29]
									*P* = 0.70^6^
Female, age <30	28.8	3,662	**0.79**	10.1	549	**1.83**	2.35	2.58	[1.17, 5.71]
Female, age 30–39	18.0	1,468	**1.22**	13.8	950	**1.46**	1.19	1.42	[0.64, 3.14]
Female, age 40–49	3.6	867	**0.42**	7.5	586	**1.28**	3.19	3.92	[0.90, 17.02]
Female, age ≥50	12.6	1,452	**0.87**	1.5	266	**0.56**	0.60	0.76	[0.10, 5.60]
Female, all ages	**63.0**	7,449	**0.85**	**32.9**	2,351	**1.40**	1.66	**1.93**	[1.14, 3.25]
									*P* = 0.40^6^
Males and females, all ages	**198.5**	**13,363**	**1.49**	**58.4**	**3,009**	**1.94**	1.31	**1.61**	**[1.13, 2.30]**

^1^ In Zambia: Overall, 13.5% self-reported they were HIV–positive and taking ART, 1.0% self-reported they were HIV–positive and not taking ART, 1.5% tested HIV–positive. In South Africa: Overall, 12.9% self-reported they were HIV–positive and taking ART, 1.3% self-reported they were HIV–positive and not taking ART, 1.9% tested HIV–positive.

^2^ For subgroup analysis of males and females, adjusted for triplet and estimating age-group-specific odds ratios to compare HIV–positive with HIV–negative individuals (interaction between age group and HIV status, in the logistic regression model); for analysis of males and females combined, adjusted for triplet and allowing the association between age group and prevalent TB to be different for males and females (interaction between sex and age group, in the logistic regression model).

^3^ Number of individuals with prevalent TB.

^4^ Total participants.

^5^ Percentage with prevalent TB.

^6^ *P*-value for evidence for variation in ORs (comparing HIV–positive to HIV–negative individuals) among the 4 age groups.

ART, antiretroviral therapy; CI, confidence interval; HIV, human immunodeficiency virus; OR, odds ratio.

### Sensitivity analyses

Findings about trial arm comparisons were similar in sensitivity analyses (S5 Table), for example in the analysis in which all culture-eligible individuals were assumed to have prevalent TB the adjusted prevalence ratio comparing arms A and B combined with arm C was 1.17 [95% CI 0.68, 2.02], and in the analysis in which all sputum-eligible individuals with missing S1 or S2 Xpert-Ultra test results were assumed not to have prevalent TB the adjusted prevalence ratio comparing arms A and B combined with arm C was 1.11 [95% CI 0.66, 1.89].

## Discussion

### Key findings

We found no evidence that the PopART intervention reduced TB prevalence through UTT for HIV and systematic symptom screening for TB, after 4 years of intervention delivery (2014 to 2017 inclusive) and with TB prevalence measured 2 to 4 years later in 2019 to 2021. This lack of evidence was found overall and also in subgroup analyses among men, women, older and younger individuals, HIV–negative and HIV–positive individuals, and among individuals who had been resident in the study communities for ≥5 years. Findings were also similar in sensitivity analyses that made different assumptions about TB prevalence among survey participants with missing data on whether or not they had TB on the day of the survey. Our findings were thus consistent across subgroups of the population and robust to alternative ways of accounting for missing data on prevalent TB. TB prevalence was on average high across the 21 study communities, at around 0.9% overall, around 1.6% averaging across South African communities, and around 0.5% averaging across Zambian communities. TB prevalence was also higher among men than women, among older than younger individuals, and around 2 times higher among HIV–positive compared with HIV–negative individuals. Most individuals who had prevalent TB were eligible to provide sputum samples for diagnostic testing only on the basis of an abnormal X-ray and not based on the TB symptoms they reported.

### Interpretation of findings and consistency with other studies

Several factors could have contributed to the lack of evidence for impact of the PopART intervention on TB prevalence. First, the TB-specific component of the PopART intervention consisted of systematic TB screening based on TB symptoms. National and subnational TB prevalence surveys have consistently found that a considerable proportion of individuals who have prevalent TB on the day of the survey are identified for diagnostic testing only on the basis of an abnormal X-ray and not based on the TB suggestive symptoms they report [15,16]. This was also found in the TREATS TB prevalence survey, in which only around 30% (40% in Zambian communities and 20% in South African communities) of individuals with prevalent TB screened positive on TB symptoms. In a national TB prevalence survey in Zambia in 2013 to 2014 [17], 61% of prevalent TB cases were eligible to provide sputum samples based on the TB symptoms they reported, with less strict criteria to screen positive on TB symptoms compared with the TREATS survey (at least one of cough ≥2 weeks, fever ≥2 weeks, chest pains ≥2 weeks). In a national TB prevalence survey in South Africa in 2017 to 2019 [18], 42% of prevalent TB cases were eligible to provide sputum samples based on the TB symptoms they reported, again with less strict criteria than in the TREATS survey (at least one of cough of any duration, night sweats for ≥2 weeks, unexplained weight loss for ≥2 weeks, unexplained fever for ≥2 weeks). Evidence is emerging that “subclinical” TB, i.e., persons not experiencing the classical TB symptoms, form a large part of the reservoir of individuals with active TB [19–21].

Second, an interim review of the TB symptom screening component of the PopART intervention identified that it was not prioritised by the CHiPs during years 1 to 2 of intervention delivery. Instead, CHiPs focused on offering universal HIV testing and supporting HIV–positive individuals to register for HIV care, because the primary goal of the intervention was to reduce HIV incidence. CHiPs gave more attention to TB symptom screening during years 3 to 4 of intervention delivery, following re-training and enhanced monitoring, but this effort may have been for too short a time to have much impact on population-wide TB incidence and prevalence. Nevertheless, among individuals who participated in the PopART intervention, the percentage who were eligible to provide sputum samples for TB diagnostic testing based on the TB symptoms they reported was around 1%, 1%, and 2%, respectively, across rounds 1 to 3 of intervention delivery in Zambian communities, the proportion who were diagnosed with TB (among all who participated in the intervention) was around 0.08%, 0.08%, and 0.1%, respectively, and in each round approximately 200,000 individuals aged ≥15 years participated. In the third round of intervention delivery in South African communities, the corresponding figures were that around 6% of individuals were eligible to provide sputum samples for TB diagnostic testing based on the TB symptoms they reported, the proportion of all intervention participants who were diagnosed with TB was 0.4%, and around 85,000 individuals aged ≥15 years participated (estimates for rounds 1 to 2 could not be made due to challenges with some aspects of data collection and recording) [22,23].

Third, we found that TB prevalence was much higher among men than women in the TREATS study communities, consistent with findings from national TB prevalence surveys in Zambia and South Africa [17,18]. However, the PopART intervention was less effective at reaching men than women, for example, in Zambian communities around two-thirds of men participated in the third round of intervention delivery compared with around 85% of women [24]. This lower coverage of the intervention among men is likely a contributing factor to the lack of overall impact of the intervention on community-wide TB prevalence.

Fourth, by the time of the TREATS TB prevalence survey in 2019 to 2021, HIV testing and ART coverage among HIV–positive individuals was on average similar in communities in the standard-of-care arm (arm C) to those in the PopART intervention arm (arms A and B). In contrast, between mid-2016 and mid-2018, the proportion of HIV–positive individuals who were virally suppressed was on average lower in the standard-of-care arm than in the PopART intervention arms [7]. Additionally, a UTT intervention may have a smaller effect on TB prevalence than on TB incidence. HIV infection, in the absence of ART, has previously been shown to increase TB prevalence to a lesser extent than TB incidence [25]. This is due to the shorter average duration of TB in PLWH compared with HIV–negative individuals [26], because symptoms progress more rapidly and so TB is identified earlier or individuals may die from TB more quickly [25,27]. It is less well understood how ART may affect this relationship. For example, an individual who is HIV–positive and on ART, while still more likely to have TB than someone who is HIV–negative, may have a disease course that is more like that of an HIV–negative person in terms of the time between disease onset and diagnosis. On the other hand, HIV–positive individuals who are in HIV care are expected to be screened for TB (based on TB symptoms) every time they attend a health clinic for HIV care, which facilitates early diagnosis of TB disease compared with HIV–positive individuals who are not in HIV care.

Fifth, the COVID pandemic disrupted access to TB diagnostic and treatment services during 2020 to 2021, and it also interrupted and delayed the completion of the TB prevalence survey. While this happened across all study communities, it may have reduced the differences among trial arms in terms of TB prevalence.

Our findings are consistent with the TREATS co-primary endpoint of the incidence of new infection with *M*. *tuberculosis* among young people during 2018 to 2021, for which there was no evidence of lower incidence in arm A compared with arm C communities [28].

### Generalisability, strengths, and limitations

The TREATS TB prevalence survey was large, including around 50,000 individuals, and covering 21 communities. Nevertheless, the study was only powered to detect fairly large reductions (of the order of 40% to 50%) in TB prevalence in the PopART intervention trial arms (arms A and B) compared with the standard-of-care arm, and also the between-community variation in TB prevalence was larger than had been anticipated. Also, the HPTN 071 (PopART) study was designed to be balanced across trial arms on pre-trial HIV prevalence, but not on indicators of TB burden, and for most of the South African communities information on pre-trial TB prevalence was not available. The proportion of individuals who participated in the survey, among all who were invited to participate, was around 5% higher in arms A and B combined compared with arm C, but the age-sex distribution and HIV prevalence among participants was similar across the trial arms, showing that the trial had good balance across trial arms on these key individual characteristics. The missing data on prevalent TB status introduced additional uncertainty to our prevalence estimates and trial arm comparisons, but imputation of missing data was done to limit bias and sensitivity analyses showed that our findings were robust to different assumptions. Lastly, the CHiPs were a similar cadre of staff as could be employed programmatically, so our study provides evidence about the effectiveness of the PopART intervention in reducing TB prevalence in close-to-programmatic conditions.

### Implications of study findings

Our findings indicate that systematic screening for TB that is based on symptom screening alone is not sufficient to achieve a large reduction in TB prevalence over a period of several years. Including chest X-ray screening alongside TB symptom screening could substantially increase the sensitivity of systematic screening for TB and is increasingly feasible with the advent of mobile X-ray machines (including ones that can be transported in a backpack) and computer-assisted diagnosis of digital X-ray images. In settings with high general population TB prevalence (such as the communities in our study), systematic screening for TB based on both symptoms and chest X-ray might be able to achieve a considerable reduction in TB prevalence and transmission, in absolute as well as relative terms; such a strategy could be evaluated in future research. The accuracy, in particular the specificity, of computer-aided diagnosis has been improved since the TREATS TB prevalence survey was conducted [29,30], bringing down the overall costs of X-ray screening by reducing the proportion of individuals who screen positive and are then eligible for diagnostic testing for TB. Additionally, it might be possible to increase the sensitivity of systematic TB screening based on TB symptoms alone through better symptoms enquiry (for example, taking more time over such enquiry to facilitate elicitation of milder or less obvious symptoms).

The 2021 WHO TB screening guidelines included a statement that “Systematic screening for TB disease may be conducted among the general population in areas with an estimated TB prevalence of 0.5% or higher,” but the evidence for this is weak. They also stated that a range of approaches to such screening could be considered, including diagnostic testing (using molecular tests) for everyone or for those who screen positive on TB symptoms or chest X-ray. Our study provides rigorous evidence about the impact of systematic TB symptom screening on TB prevalence in communities with an average prevalence of >0.5%, which can be taken into account in future updates to these guidelines.

Our study does not provide clear evidence about the effect of UTT for HIV on TB prevalence, because ART coverage was high in all trial arms by the time of the TREATS TB prevalence survey. However, TB prevalence remained approximately 2 times higher among HIV–positive than HIV–negative individuals, after adjusting for sex and age group and study triplet, compared to around 3 to 4 times higher in similar study communities in the ZAMSTAR trial TB prevalence survey that was conducted in 2010 when ART coverage was much lower [31]. This suggests that the overall impact of increased coverage of HIV care and ART is to reduce TB prevalence among HIV–positive individuals.

## Conclusions

There was no evidence that the PopART intervention reduced TB prevalence. Potential explanations include: similarly high coverage of HIV testing and treatment in all trial arms by 2019 to 2021; systematic TB symptom screening missing subclinical TB; ART coverage affecting TB prevalence less than TB incidence; the initially lower priority given by the CHiPs to the TB screening component of the PopART intervention; lower coverage of the PopART intervention among men than women; and COVID-19 pandemic effects on TB prevalence during 2020 to 2021. Our findings indicate that the overall impact of increased coverage of HIV care and ART is to reduce TB prevalence among HIV–positive individuals.

## Supporting information

S1 Fig Sputum eligibility definition. (TIF)

S2 Fig Culture eligibility algorithm based on the results of Xpert-Ultra testing on 2 sputum samples (S1 and S2). (TIF)

S3 Fig Decision tree for culture eligibility and collection of S3 and S4 samples. (TIF)

S4 Fig Algorithm to determine final culture result based on the culture results from each of 2 MGIT tubes. (TIF)

S5 Fig Flow diagram by country of participant enrolment, sample collection, and MTB detection. (TIF)

S6 Fig Flow diagram by 3 trial arms of participant enrolment, sample collection, and MTB detection. (TIF)

S1 Text Culture testing of sputum samples. (DOCX)

S2 Text Details on missing value imputation. (DOCX)

S1 Table Characteristics of the TREATS study participants population by arm, country, and overall totals. (DOCX)

S2 Table HIV indicators collected during TREATS by arm, country, and overall totals. (DOCX)

S3 Table Characteristics of participants with prevalent TB, by trial arm and country. (DOCX)

S4 Table TB prevalence by country, comparing individuals who self-reported they were HIV–positive and taking ART with HIV–negative individuals, overall and stratified by sex and age group. (DOCX)

S5 Table TB prevalence by trial arm, sensitivity analyses. (DOCX)

S1 Consort Checklist CONSORT 2010 checklist of information to include when reporting a cluster-randomised trial. Extension of CONSORT for abstracts to reports of cluster-randomised trials.
(PDF)

S1 Protocol Tuberculosis Reduction through Expanded Anti-Retroviral Treatment and Screening (TREATS) Project. (PDF)

S1 SAP Statistical analysis plan. (PDF)

## Abbreviations

ART: antiretroviral therapy
CAB: Community Advisory Board
CHiP: community HIV care provider
CI: confidence interval
COVID-19: Coronavirus Disease 2019
DMS: data management system
HPTN: HIV Prevention Trials Network
IDP: intensive diagnostic phase
LIMS: laboratory information management system
MFS: mobile field site
MGIT: mycobacteria growth indicator tube
MTB: *M*. *tuberculosis*
NHLS: National Health Laboratory Service
PLWH: people living with HIV
TB: tuberculosis
TREATS: Tuberculosis Reduction through Expanded Anti-retroviral Treatment and Screening
UTT: universal testing and treatment
