# Supplementary figures and images for: Tuberculosis prevalence after 4 years of population-wide systematic TB symptom screening and universal testing and treatment for HIV in the HPTN 071 (PopART) community-randomised trial in Zambia and South Africa: A cross-sectional survey (TREATS)
Source: PLoS Med. 2023 Sep 8;20(9):e1004278. doi: 10.1371/journal.pmed.1004278 (PMC10490889; doi:10.1371/journal.pmed.1004278)

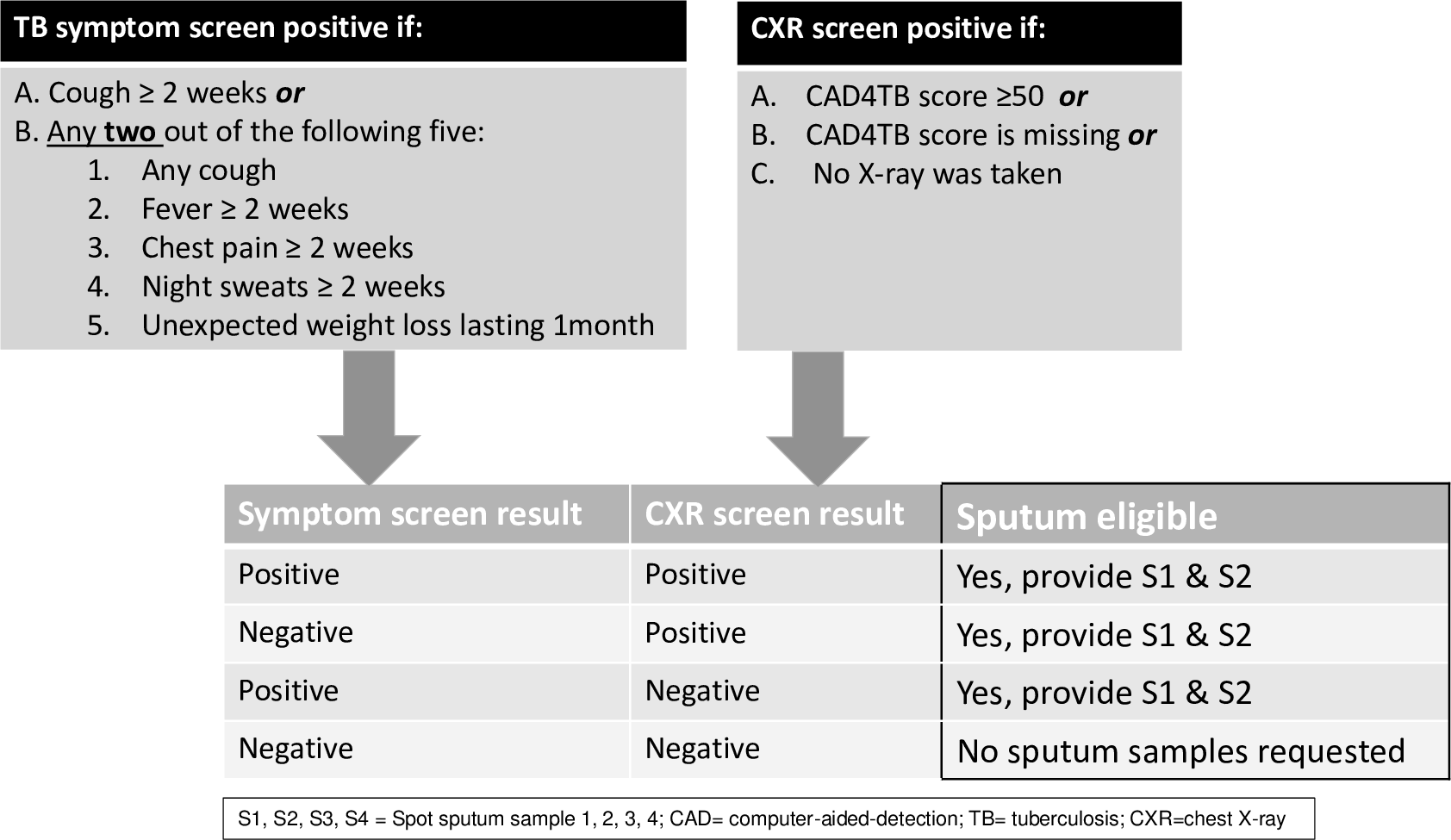

Supplement: S1 Fig — (TIF) [file pmed.1004278.s001.tif]

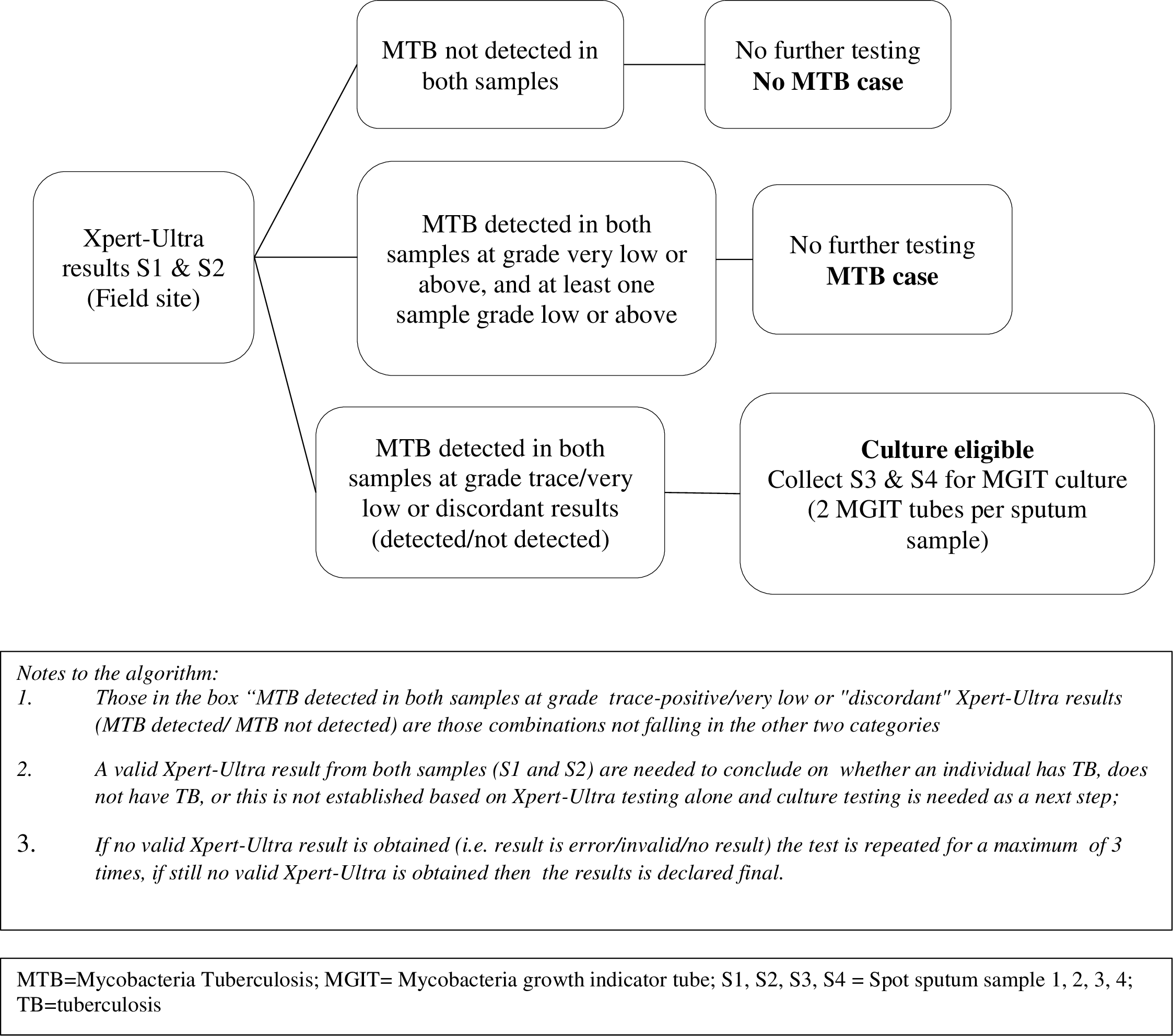

Supplement: S2 Fig — (TIF) [file pmed.1004278.s002.tif]

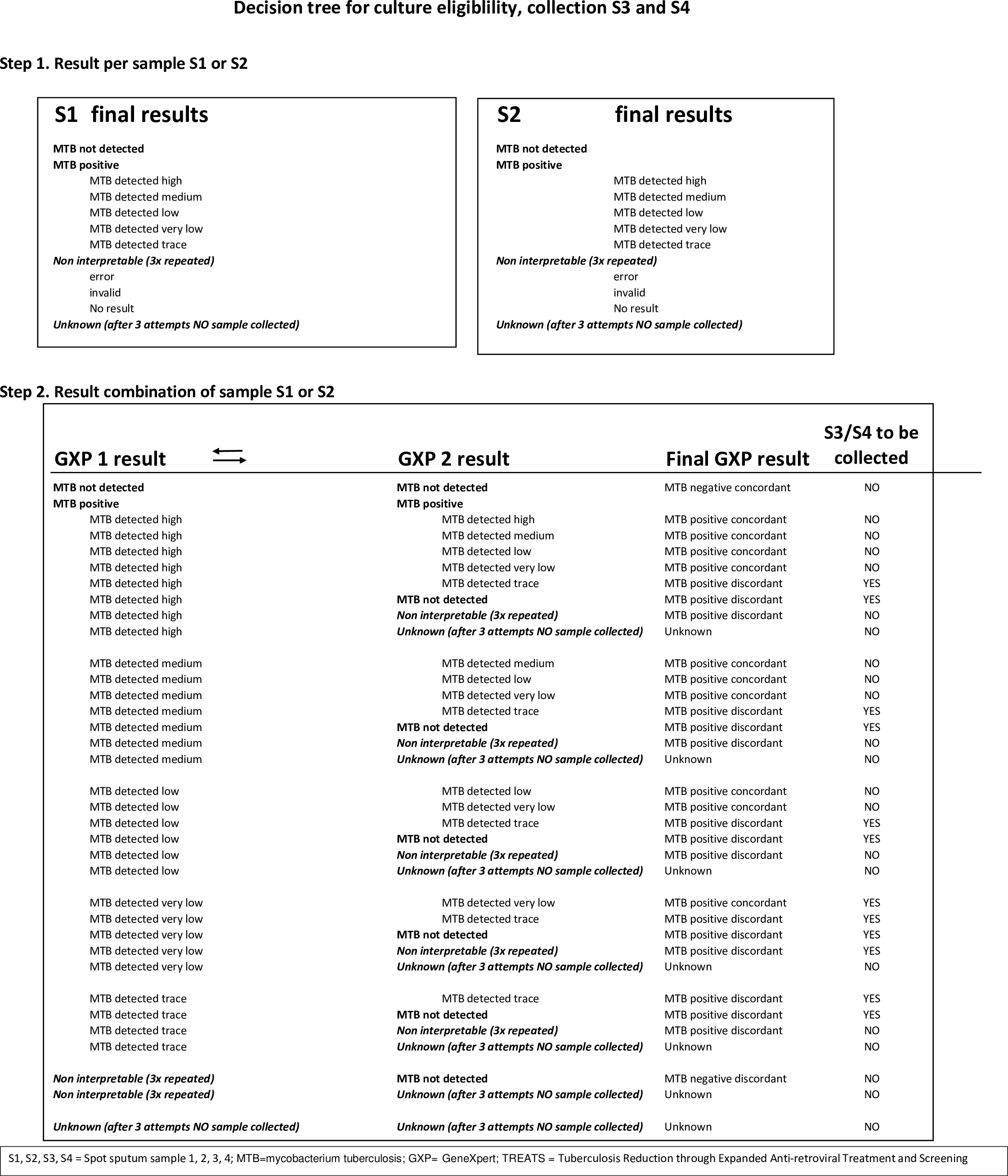

Supplement: S3 Fig — (TIF) [file pmed.1004278.s003.tif]

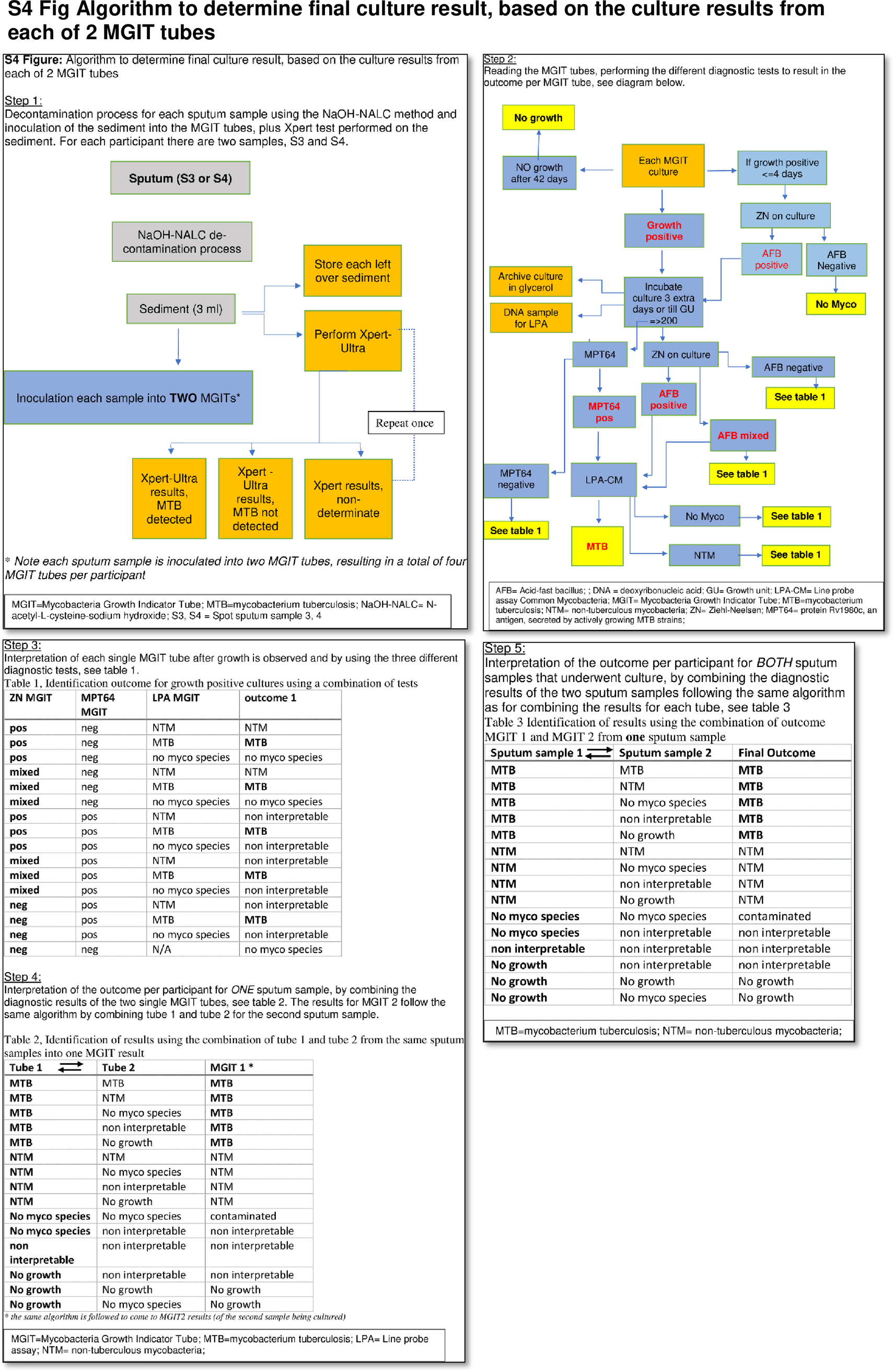

Supplement: S4 Fig — (TIF) [file pmed.1004278.s004.tif]

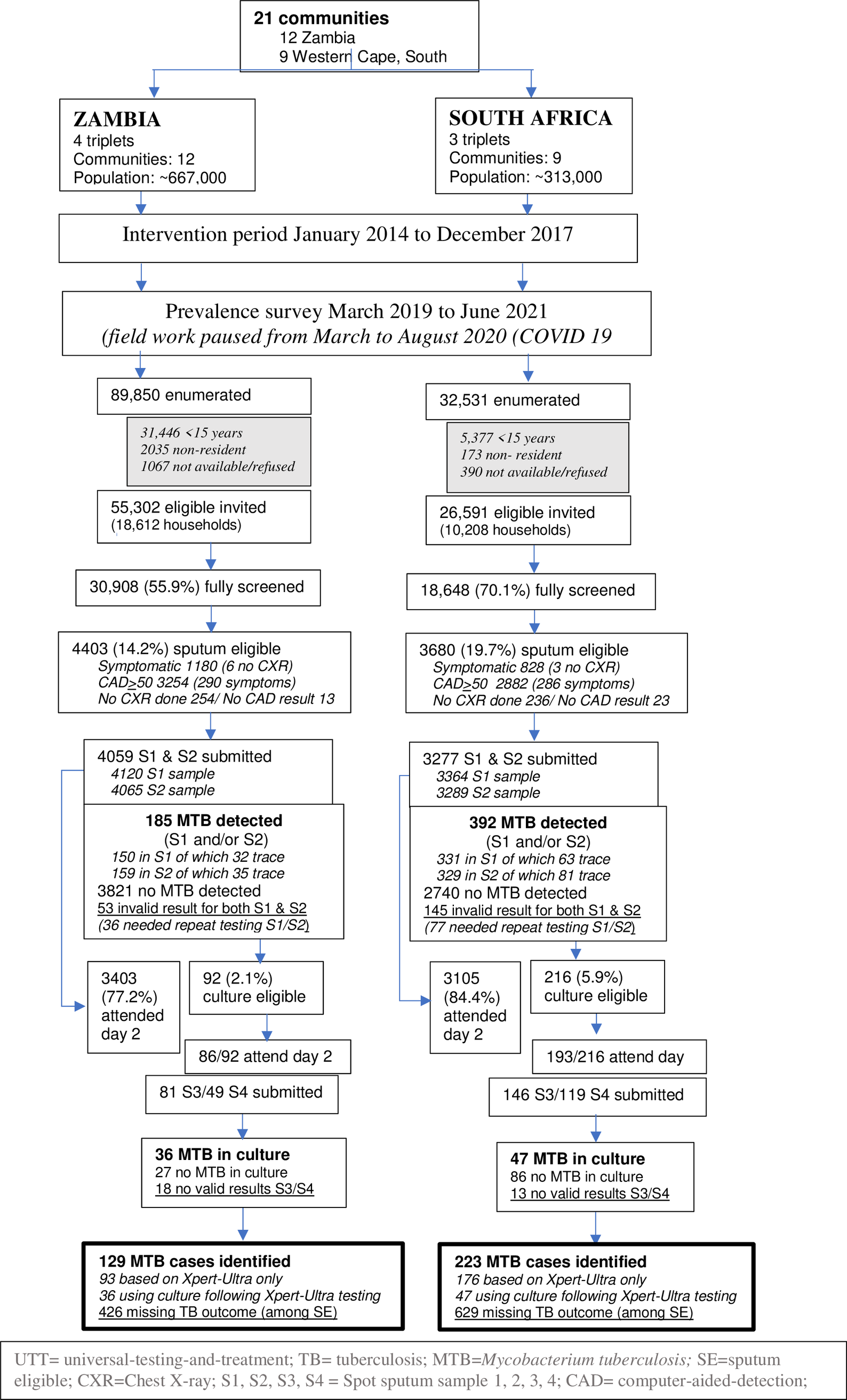

Supplement: S5 Fig — (TIF) [file pmed.1004278.s005.tif]

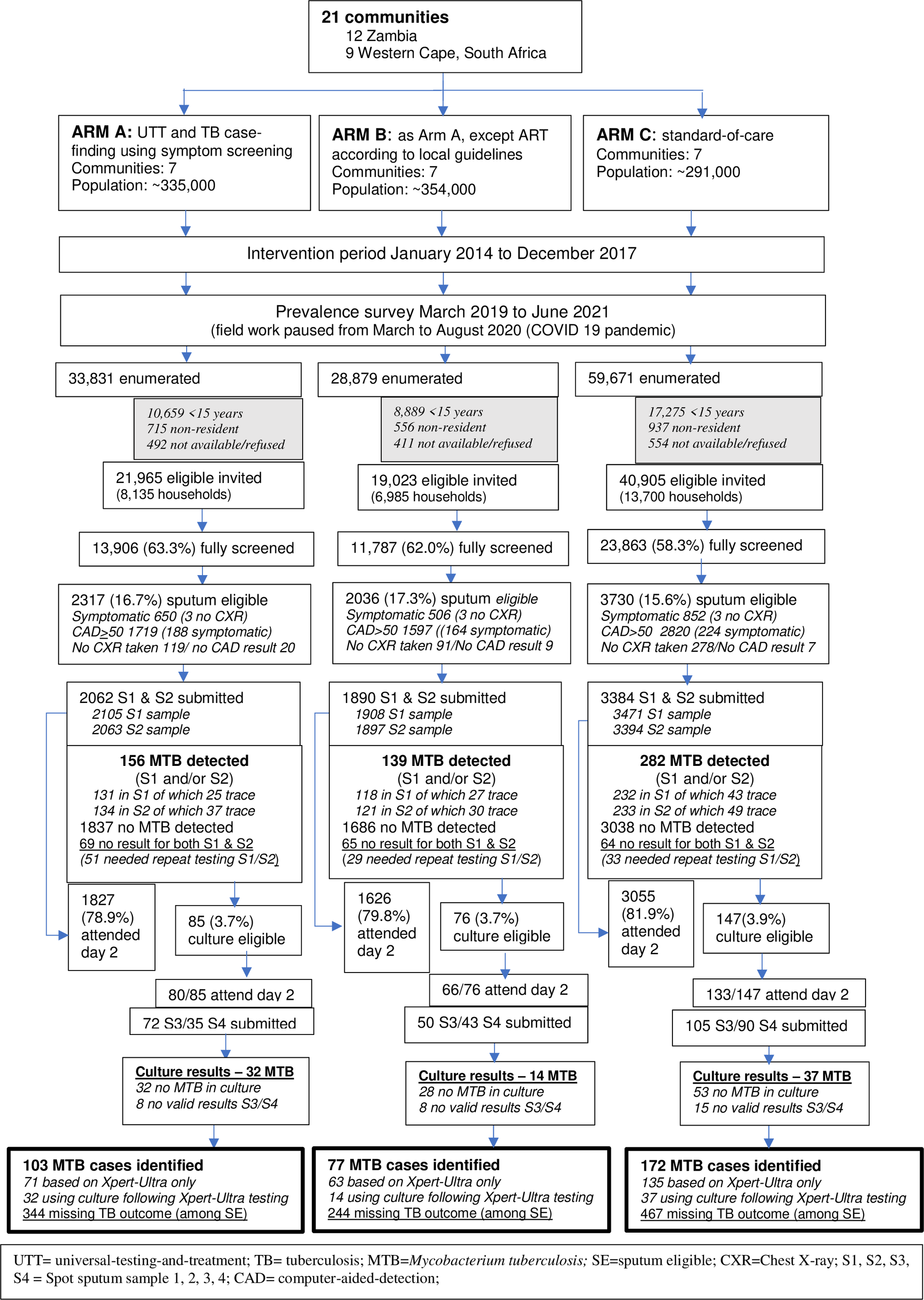

Supplement: S6 Fig — (TIF) [file pmed.1004278.s006.tif]
