# Supplementary material for: Tuberculosis prevalence after 4 years of population-wide systematic TB symptom screening and universal testing and treatment for HIV in the HPTN 071 (PopART) community-randomised trial in Zambia and South Africa: A cross-sectional survey (TREATS)
Source: PLoS Med. 2023 Sep 8;20(9):e1004278. doi: 10.1371/journal.pmed.1004278 (PMC10490889; doi:10.1371/journal.pmed.1004278)
Supplement: S1 Text — (DOCX) [file pmed.1004278.s007.docx]

**S1 text Culture testing of sputum samples**

All sputum samples that were collected for culture testing were batched and kept in a refrigerator until being transported on the same day to the central laboratory in a cooler box. In Zambia, samples were taken to the Zambart central laboratory in Lusaka, and in South Africa to the National-Health-Laboratory-Service (NHLS) laboratory in Greenpoint, Cape Town.

At the culture laboratory, samples were decontaminated using the standard N-acetyl-l-cysteine-sodium-hydroxide (NALC-NaOH) method^[[1]](#footnote-1)^ and after decontamination the resuspended sediment from each sample was inoculated onto two mycobacteria growth indicator tubes (MGIT) and incubated for 42 days or until growth was observed. Decontamination of the samples was done in batches, with each batch including one low-MTB-bacillary-load positive control and one negative control sample.

Growth-positive cultures were tested using culture Ziehl-Neelsen (ZN) staining and *MPT*64 antigen testing to distinguish *M. tuberculosis* from non-tuberculous mycobacteria (NTM) and from contamination, and those that showed acid-fast bacilli or were *MPT*64 positive were tested using the line probe assay (LPA) for MTB complex and clinically relevant NTM (LPA-CM, HAIN). The culture result for each tube was defined based on the combination of the three test results (ZN, *MPT*64 and LPA) as follows: (i) negative (no growth observed) (ii) *M. tuberculosis* (iii) NTM (iv) non-interpretable (when test results were conflicting) or (v) contaminated (S4 Figure).

The culture result for each sputum sample was defined based on the combination of the culture results from the 2 tubes. A sample was classified as culture-positive for *M. tuberculosis* if ≥1 tube result was positive for *M. tuberculosis*. Among samples that were not culture-positive for *M. tuberculosis*, samples were classified as culture-negative if ≥1 tube was culture-negative or ≥1 tube was positive for NTM. The result was classified as contaminated if both tubes were contaminated, and as non-interpretable if either both tubes were non-interpretable, or one was non-interpretable and one was contaminated. We defined a sputum sample culture result as valid if it was culture-positive for *M. tuberculosis* or if it was culture-negative, and also from a batch where the positive control grew and the negative control did not; other results were classified as missing.

The culture result for each individual was defined based on the combination of the culture results from the S3 and S4 samples. An individual had a valid culture result if one or both of the S3 and S4 sample results were valid and was classified as having prevalent TB if ≥1 sample was culture-positive for *M. tuberculosis*, and as not having prevalent TB if neither sample was culture-positive for *M. tuberculosis* and ≥1 sample was culture-negative, with other individuals classified as having missing data on their culture status.

1. GLI, 2014 Mycobacteriology laboratory manual. [↑](#footnote-ref-1)
