# Supplementary material for: Tuberculosis prevalence after 4 years of population-wide systematic TB symptom screening and universal testing and treatment for HIV in the HPTN 071 (PopART) community-randomised trial in Zambia and South Africa: A cross-sectional survey (TREATS)
Source: PLoS Med. 2023 Sep 8;20(9):e1004278. doi: 10.1371/journal.pmed.1004278 (PMC10490889; doi:10.1371/journal.pmed.1004278)
Supplement: S2 Text — (DOCX) [file pmed.1004278.s008.docx]

**S2 Text Details on missing value imputation**

For sputum eligible participants whose prevalent TB status could not be determined due to missing Xpert-Ultra or culture test results, imputation of the missing data was done in two steps. The first step was to impute missing data on prevalent TB (yes or no) among culture-eligible individuals with missing data on culture results. Using the data on individuals whose culture status was observed (positive or negative), tabulations and univariable and multivariable logistic regression were used to identify the strongest predictors of culture status; the strongest predictors were Xpert test results on S1 and S2 (classified as trace-positive only, versus ≥1 sample with a grade of very-low or above), previous history of TB treatment (yes, no, currently on TB treatment), and country. A logistic regression model, using these predictor variables, was then used to multiply impute the missing data on prevalent TB status, creating 30 datasets that each included all of the culture-eligible individuals and in which all of them were classified as yes or no for prevalent TB. Each of these 30 imputed datasets was then combined with a dataset that included all the sputum-eligible individuals who were *not* culture-eligible, to create 30 datasets that included all sputum-eligible individuals. For each of these 30 datasets, the second step (of multiple imputation) was to impute missing data on prevalent TB (yes or no) for the sputum-eligible individuals who had missing Xpert-Ultra test results. A logistic regression model, with predictor variables consisting of previous history of TB treatment, X-ray CAD score (categories <50, 50-69, 70-84, ≥85), sputum-eligible based on TB symptoms (yes or no), HIV and ART status, age group, sex, and community, was used to multiply impute the missing data on prevalent TB status. The multiple imputation was done with stratification on country and trial arm (Arms A and B combined, Arm C), and 10 imputed datasets were created from each of the 30 datasets that were generated in the first step. The rationale for 30 imputed datasets at step 1 was that about 30% of culture results were missing. The rationale for 10 imputed datasets (for each of the 30 datasets created at step 1) was that about 10% of sputum-eligible individuals did not have two Xpert-Ultra test results. This gave a total of 300 imputed datasets that each included all of the sputum-eligible individuals and in which all of them were classified as yes or no for prevalent TB; each of these 300 imputed datasets was then combined with a dataset that included all the individuals who were not sputum-eligible, to create 300 imputed datasets that included all survey participants.
