# Supplementary material for: Tuberculosis prevalence after 4 years of population-wide systematic TB symptom screening and universal testing and treatment for HIV in the HPTN 071 (PopART) community-randomised trial in Zambia and South Africa: A cross-sectional survey (TREATS)
Source: PLoS Med. 2023 Sep 8;20(9):e1004278. doi: 10.1371/journal.pmed.1004278 (PMC10490889; doi:10.1371/journal.pmed.1004278)
Supplement: S1 Table — (DOCX) [file pmed.1004278.s009.docx]

**S1 Table Characteristics of the TREATS study participants population by arm, country and overall totals**

|  | **Arm A** | | **Arm B** | | **Arm C** | | **combined** | | | **Zambia** | | | **South Africa** | | |  |
| --- | --- | --- | --- | --- | --- | --- | --- | --- | --- | --- | --- | --- | --- | --- | --- | --- |
|  | **n** | **%** | **n** | **%** | **n** | **%** | | **n** | **%** | | **n** | **%** | | **n** | **%** | |
| **Number seen at site (fully screened)** | **13,906** |  | **11,787** |  | **23,863** |  | | **49,556** |  | | **30,908** |  | | **18,648** |  | |
| **#/% female** | 8,552 | 61.5% | 7,282 | 61.8% | 14,252 | 59.7% | | 30,086 | 60.7% | | 19,154 | 62.0% | | 10,932 | 58.6% | |
| **age group** |  |  |  |  |  |  | |  |  | |  |  | |  |  | |
| 15-24 years | 5,056 | 36.4% | 4,417 | 37.5% | 9,114 | 38.2% | | 18,587 | 37.5% | | 12,696 | 41.1% | | 5,891 | 31.6% | |
| 25-34 years | 3,639 | 26.2% | 2,930 | 24.9% | 6,073 | 25.4% | | 12,642 | 25.5% | | 7,714 | 25.0% | | 4,928 | 26.4% | |
| 35-44 years | 2,357 | 16.9% | 1,995 | 16.9% | 3,866 | 16.2% | | 8,218 | 16.6% | | 4,943 | 16.0% | | 3,275 | 17.6% | |
| 45-54 years | 1,418 | 10.2% | 1,205 | 10.2% | 2,288 | 9.6% | | 4,911 | 9.9% | | 2,601 | 8.4% | | 2,310 | 12.4% | |
| 55+ years | 1,436 | 10.3% | 1,240 | 10.5% | 2,522 | 10.6% | | 5,198 | 10.5% | | 2,954 | 9.6% | | 2,244 | 12.0% | |
| **length of residence** |  |  |  |  |  |  | |  |  | |  |  | |  |  | |
| <=1 years | 685 | 4.9% | 570 | 4.8% | 1,583 | 6.6% | | 2,838 | 5.7% | | 2,313 | 7.5% | | 525 | 2.8% | |
| 1-5 years | 2,984 | 21.5% | 2,528 | 21.4% | 5,594 | 23.4% | | 11,106 | 22.4% | | 7,957 | 25.7% | | 3,149 | 16.9% | |
| 5-10 years | 3,226 | 23.2% | 2,909 | 24.7% | 4,900 | 20.5% | | 11,035 | 22.3% | | 5,677 | 18.4% | | 5,358 | 28.7% | |
| >10 years | 7,011 | 50.4% | 5,780 | 49.0% | 11,786 | 49.4% | | 24,577 | 49.6% | | 14,961 | 48.4% | | 9,616 | 51.6% | |
| **education level** |  |  |  |  |  |  | |  |  | |  |  | |  |  | |
| None | 483 | 3.5% | 502 | 4.3% | 626 | 2.6% | | 1,611 | 3.3% | | 1,161 | 3.8% | | 450 | 2.4% | |
| Primary school | 3,412 | 24.5% | 2,750 | 23.3% | 4,791 | 20.1% | | 10,953 | 22.1% | | 9,017 | 29.2% | | 1,936 | 10.4% | |
| Secondary school grade | 9,405 | 67.6% | 8,060 | 68.4% | 17,276 | 72.4% | | 34,741 | 70.1% | | 18,946 | 61.3% | | 15,795 | 84.7% | |
| Higher education | 606 | 4.4% | 475 | 4.0% | 1,170 | 4.9% | | 2,251 | 4.5% | | 1,784 | 5.8% | | 467 | 2.5% | |
|  |  |  |  |  |  |  | |  |  | |  |  | |  |  | |
| **history TB treatment** |  |  |  |  |  |  | |  |  | |  |  | |  |  | |
| Previous TB | 1304 | 9.4% | 976 | 8.3% | 2076 | 8.7% | | 4,356 | 8.8% | | 1,714 | 5.5% | | 2,642 | 14.2% | |
| current TB | 128 | 0.9% | 76 | 0.6% | 91 | 0.4% | | 295 | 0.6% | | 99 | 0.3% | | 196 | 1.1% | |
| **HIV status (after TREATs)** |  |  |  |  |  |  | |  |  | |  |  | |  |  | |
| Negative | 10688 | 76.9% | 8916 | 75.6% | 17391 | 72.9% | | 36,995 | 74.7% | | 23,632 | 76.5% | | 13,363 | 71.7% | |
| Positive | 2322 | 16.7% | 1847 | 15.7% | 3808 | 16.0% | | 7,977 | 16.1% | | 4,968 | 16.1% | | 3,009 | 16.1% | |
| Unknown | 896 | 6.4% | 1024 | 8.7% | 2664 | 11.2% | | 4,584 | 9.3% | | 2,308 | 7.5% | | 2,276 | 12.2% | |

*TB=tuberculosis; HIV=human immunodeficiency virus; TREATS =Tuberculosis Reduction through Expanded Anti-retroviral Treatment and Screening*
