# Supplementary material for: Tuberculosis prevalence after 4 years of population-wide systematic TB symptom screening and universal testing and treatment for HIV in the HPTN 071 (PopART) community-randomised trial in Zambia and South Africa: A cross-sectional survey (TREATS)
Source: PLoS Med. 2023 Sep 8;20(9):e1004278. doi: 10.1371/journal.pmed.1004278 (PMC10490889; doi:10.1371/journal.pmed.1004278)
Supplement: S2 Table — (DOCX) [file pmed.1004278.s010.docx]

**S2 Table HIV indicators collected during TREATS by arm, country and overall totals**

|  | **Arm A** | | | **Arm B** | | **Arm C** | | **combined** | | **Zambia** | | **South Africa** | |
| --- | --- | --- | --- | --- | --- | --- | --- | --- | --- | --- | --- | --- | --- |
|  | **n** | **%** | **n** | | **%** | **n** | **%** | **n** | **%** | **n** | **%** | **n** | **%** |
| Total participants | 13,906 |  | 11,787 | |  | 23,863 |  | 49,556 |  | 30,908 |  | 18,648 |  |
| **HIV interview accepted** | **13,432** | **96.6%** | **11,282** | | **95.7%** | **22,869** | **95.8%** | **47,583** | **96.0%** | **30,018** | **97.1%** | **17,565** | **94.2%** |
| HIV ever tested | 12,277 | 91.4% | 10,096 | | 89.5% | 19,635 | 85.9% | 42,008 | 88.3% | 26,226 | 87.4% | 15,784 | 89.9% |
| **HIV known status (last 12m)** |  | 71.4% |  | | 67.0% |  | 67.0% |  | 68.2% |  | 68.5% |  | 67.9% |
| *Negative* | 7,816 | 58.2% | 6,292 | | 55.8% | 12,527 | 54.8% | 26,635 | 56.0% | 16,647 | 55.5% | 9,990 | 56.9% |
| *Positive* | 2,116 | 15.8% | 1,609 | | 14.3% | 3,456 | 15.1% | 7,181 | 15.1% | 4,512 | 15.0% | 2,665 | 15.2% |
| *Unknown* | 3,500 | 26.1% | 3,381 | | 30.0% | 6,886 | 30.1% | 13,767 | 28.9% | 8,859 | 29.5% | 4,910 | 28.0% |
| **On ART (PLHIV)** |  |  |  | |  |  |  |  |  |  |  |  |  |
| *Ever* | 2,069 | 97.8% | 1,533 | | 95.3% | 3,317 | 96.0% | 6,919 | 96.4% | 4,335 | 96.1% | 2,583 | 96.9% |
| *Current* | 1,966 | 92.9% | 1,476 | | 91.7% | 3,171 | 91.8% | 6,613 | 92.1% | 4,197 | 93.0% | 2,415 | 90.6% |
| **Ever taken IPT*** |  |  |  | |  |  |  |  |  |  |  |  |  |
| *Yes current* | 212 | 10.1% | 181 | | 11.3% | 352 | 10.3% | 745 | 10.5% | 671 | 15.0% | 74 | 2.8% |
| *Yes completed* | 486 | 23.3% | 362 | | 22.7% | 724 | 21.1% | 1,572 | 22.1% | 1,085 | 24.2% | 487 | 18.5% |
| *No* | 1,392 | 66.6% | 1,054 | | 66.0% | 2,357 | 68.7% | 4,803 | 67.5% | 2,731 | 60.9% | 2,072 | 78.7% |
| Acceptance HIV testing  (among those offered testing) | 8,438/  11,316 | 74.6% | 7,831/  9,673 | | 81.0% | 13,623/  19,413 | 70.2% | 29,892/  40,402 | 74.0% | 19,119/  25,503 | 75.0% | 10,773/  14,899 | 72.3% |
| HIV newly diagnosed  (among those tested) | 208 | 2.5% | 238 | | 3.0% | 357 | 2.6% | 803 | 2.7% | 457 | 2.4% | 346 | 3.2% |
| **HIV-ART status (after TREATs)** |  |  |  | |  |  |  |  |  |  |  |  |  |
| *Negative* | 10,688 | 76.9% | 8,916 | | 75.6% | 17,391 | 72.9% | 36,995 | 74.7% | 23,632 | 76.5% | 13,363 | 71.7% |
| *Self-reported Positive, on ART* | 1,962 | 14.1% | 1,476 | | 12.5% | 3,160 | 13.2% | 6,598 | 13.3% | 4,187 | 13.5% | 2,411 | 12.9% |
| *Self-reported Positive, not on ART* | 151 | 1.1% | 133 | | 1.1% | 285 | 1.2% | 569 | 1.1% | 318 | 1.0% | 251 | 1.3% |
| *Tested HIV-positive in survey* | 209 | 1.5% | 238 | | 2.0% | 363 | 1.5% | 810 | 1.6% | 463 | 1.5% | 347 | 1.9% |
| *Unknown#* | 896 | 6.4% | 1,024 | | 8.7% | 2,664 | 11.2% | 4,584 | 9.3% | 2,308 | 7.5% | 2,276 | 12.2% |

**if known HIV positive; #The unknown group here is including those that did not participate in the HIV interview; PC= population cohort; IPT: Isoniazid Preventive Therapy;* *HIV=human immunodeficiency virus; ART=antiretroviral therapy; TREATS =Tuberculosis Reduction through Expanded Anti-retroviral Treatment and Screening*
