# Supplementary material for: Tuberculosis prevalence after 4 years of population-wide systematic TB symptom screening and universal testing and treatment for HIV in the HPTN 071 (PopART) community-randomised trial in Zambia and South Africa: A cross-sectional survey (TREATS)
Source: PLoS Med. 2023 Sep 8;20(9):e1004278. doi: 10.1371/journal.pmed.1004278 (PMC10490889; doi:10.1371/journal.pmed.1004278)
Supplement: S3 Table — (DOCX) [file pmed.1004278.s011.docx]

**S3 Table Characteristics of participants with prevalent TB, by trial arm and country**

| **TB cases** | **combined** | | **Arm A** | | **Arm B** | | **Arm C** | | **Zambia** | | **South Africa** | |
| --- | --- | --- | --- | --- | --- | --- | --- | --- | --- | --- | --- | --- |
|  | **n** | **%** | **n** | **%** | **n** | **%** | **n** | **%** | **n** | **%** | **n** | **%** |
| **Number identified** | **352** | **100.0%** | **103** | **29.3%** | **77** | **21.9%** | **172** | **48.9%** | **129** | **36.6%** | **223** | **63.4%** |
| **#/% female** | 107 | 30.4% | 25 | 24.3% | 19 | 24.7% | 63 | 36.6% | 30 | 23.3% | 77 | 34.5% |
| **age group** |  |  |  |  |  |  |  |  |  |  |  |  |
| 15-24 yrs | 62 | 17.6% | 25 | 24.3% | 10 | 13.0% | 27 | 15.7% | 22 | 17.1% | 40 | 17.9% |
| 25-34 yrs | 89 | 25.3% | 22 | 21.4% | 20 | 26.0% | 47 | 27.3% | 30 | 23.3% | 59 | 26.5% |
| 35-44 yrs | 101 | 28.7% | 21 | 20.4% | 22 | 28.6% | 55 | 32.0% | 48 | 37.2% | 53 | 23.8% |
| 45-54 yrs | 54 | 15.3% | 18 | 17.5% | 16 | 20.8% | 20 | 11.6% | 19 | 14.7% | 35 | 15.7% |
| 55+ yrs | 46 | 13.1% | 14 | 13.6% | 9 | 11.7% | 23 | 13.4% | 10 | 7.8% | 36 | 16.1% |
| **history TB treatment** |  |  |  |  |  |  |  |  |  |  |  |  |
| Previous TB | 90 | 25.6% | 30 | 29.1% | 18 | 23.4% | 42 | 24.4% | 21 | 16.3% | 69 | 30.9% |
| current TB | 21 | 6.0% | 6 | 5.8% | 8 | 10.4% | 7 | 4.1% | 7 | 5.4% | 14 | 6.3% |
| no previous TB | 241 | 68.5% | 67 | 65.0% | 51 | 66.2% | 123 | 71.5% | 101 | 78.3% | 140 | 62.8% |
| **HIV status (after TREATs)** |  |  |  |  |  |  |  |  |  |  |  |  |
| Negative | 236 | 67.0% | 70 | 68.0% | 51 | 66.2% | 115 | 66.9% | 83 | 64.3% | 153 | 68.6% |
| Positive | 82 | 23.3% | 24 | 23.3% | 16 | 20.8% | 42 | 24.4% | 40 | 31.0% | 42 | 18.8% |
| Unknown | 34 | 9.7% | 9 | 8.7% | 10 | 13.0% | 15 | 8.7% | 6 | 4.7% | 28 | 12.6% |
| **CAD score group** |  |  |  |  |  |  |  |  |  |  |  |  |
| <50 | 13 | 3.7% | 4 | 3.9% | 3 | 3.9% | 6 | 3.5% | 8 | 6.2% | 5 | 2.2% |
| >=50 | 337 | 95.7% | 98 | 95.1% | 74 | 96.1% | 165 | 95.9% | 121 | 93.8% | 216 | 96.9% |
| missing | 2 | 0.6% | 1 | 1.0% | 0 | 0.0% | 1 | 0.6% | 0 | 0.0% | 2 | 0.9% |
| **Symptom screen result** |  |  |  |  |  |  |  |  |  |  |  |  |
| Negative | 253 | 71.9% | 73 | 70.9% | 52 | 67.5% | 128 | 74.4% | 75 | 58.1% | 178 | 79.8% |
| Positive | 99 | 28.1% | 30 | 29.1% | 25 | 32.5% | 44 | 25.6% | 54 | 41.9% | 45 | 20.2% |
| **Reported symptoms** |  |  |  |  |  |  |  |  |  |  |  |  |
| *cough (any)* | 130 | 36.9% | 37 | 35.9% | 32 | 41.6% | 61 | 35.5% | 64 | 49.6% | 66 | 29.6% |
| *cough* >*2 weeks* | 81 | 23.0% | 26 | 25.2% | 22 | 28.6% | 33 | 19.2% | 44 | 34.1% | 37 | 16.6% |
| *fever* >*2 weeks* | 16 | 4.6% | 5 | 4.9% | 1 | 1.3% | 10 | 5.8% | 9 | 7.0% | 7 | 3.1% |
| *Chest pain* >*2 weeks* | 50 | 14.2% | 19 | 18.5% | 13 | 16.9% | 18 | 10.5% | 31 | 24.0% | 19 | 8.5% |
| *night sweats* >*2 weeks* | 40 | 15.1% | 13 | 12.6% | 6 | 7.8% | 21 | 12.2% | 13 | 10.1% | 27 | 12.1% |
| weight loss > 4 weeks | 39 | 11.1% | 13 | 12.6% | 6 | 7.8% | 20 | 11.6% | 17 | 13.2% | 22 | 9.9% |
| **Sputum eligibility reason** |  |  |  |  |  |  |  |  |  |  |  |  |
| *symptoms only* | 12 | 3.4% | 4 | 3.9% | 2 | 2.6% | 6 | 3.5% | 7 | 5.4% | 5 | 2.2% |
| *X-ray only* | 253 | 71.9% | 73 | 70.9% | 52 | 67.5% | 128 | 74.4% | 75 | 58.1% | 178 | 79.8% |
| *both* | 87 | 24.7% | 26 | 25.2% | 23 | 29.9% | 38 | 22.1% | 47 | 36.4% | 40 | 17.9% |
| **Bacteriological confirmation** |  |  |  |  |  |  |  |  |  |  |  |  |
| Xpert only | 269 | 76.4% | 71 | 68.9% | 63 | 81.8% | 135 | 78.5% | 93 | 72.1% | 176 | 78.9% |
| Xpert and culture | 83 | 23.6% | 32 | 31.1% | 14 | 18.2% | 37 | 21.5% | 36 | 27.9% | 47 | 21.1% |

| **Level of detection (Xpert)** |  |  |  |  |  |  |  |  |  |  |  |  |
| --- | --- | --- | --- | --- | --- | --- | --- | --- | --- | --- | --- | --- |
| no MTB / trace | 12 | 3.4% | 5 | 4.9% | 0 | 0.0% | 7 | 4.1% | 4 | 3.1% | 8 | 3.6% |
| trace/trace | 9 | 2.6% | 4 | 3.9% | 2 | 2.6% | 3 | 1.7% | 7 | 5.4% | 2 | 0.9% |
| trace/verylow | 13 | 3.7% | 8 | 7.8% | 4 | 5.2% | 1 | 0.6% | 7 | 5.4% | 6 | 2.7% |
| trace/low | 7 | 2.0% | 2 | 1.9% | 0 | 0.0% | 5 | 2.9% | 0 | 0.0% | 7 | 3.1% |
| no MTB/very low | 25 | 7.1% | 6 | 5.8% | 5 | 6.5% | 14 | 8.1% | 14 | 10.9% | 11 | 4.9% |
| no MTB/low | 10 | 2.8% | 3 | 2.9% | 2 | 2.6% | 5 | 2.9% | 3 | 2.3% | 7 | 3.1% |
| no MTB/high | 1 | 0.3% | 1 | 1.0% | 0 | 0.0% | 0 | 0.0% | 0 | 0.0% | 1 | 0.4% |
| very low/very low | 6 | 1.7% | 3 | 2.9% | 1 | 1.3% | 2 | 1.2% | 1 | 0.8% | 5 | 2.2% |
| very low/low | 45 | 12.8% | 16 | 15.5% | 10 | 13.0% | 19 | 11.0% | 18 | 14.0% | 27 | 12.1% |
| very low/med | 3 | 0.9% | 0 | 0.0% | 1 | 1.3% | 2 | 1.2% | 1 | 0.8% | 2 | 0.9% |
| low/low | 81 | 23.0% | 18 | 17.5% | 21 | 27.3% | 42 | 24.4% | 29 | 22.5% | 52 | 23.3% |
| low/medium | 35 | 9.9% | 4 | 3.9% | 8 | 10.4% | 23 | 13.4% | 8 | 6.2% | 27 | 12.1% |
| low/high | 9 | 2.6% | 1 | 1.0% | 2 | 2.6% | 6 | 3.5% | 5 | 3.9% | 4 | 1.8% |
| medium/med | 16 | 4.5% | 8 | 7.8% | 3 | 3.9% | 5 | 2.9% | 7 | 5.4% | 9 | 4.0% |
| medium/high | 34 | 9.7% | 9 | 8.7% | 11 | 14.3% | 14 | 8.1% | 10 | 7.8% | 24 | 10.8% |
| high/high | 46 | 13.1% | 15 | 14.6% | 7 | 9.1% | 24 | 14.0% | 15 | 11.6% | 31 | 13.9% |

*CAD= computer-aided-detection; CXR=chest X-ray; HIV=human immunodeficiency virus; MTB=mycobacterium tuberculosis; TB= tuberculosis; TREATS =Tuberculosis Reduction through Expanded Anti-retroviral Treatment and Screening*
