# Supplementary material for: Tuberculosis prevalence after 4 years of population-wide systematic TB symptom screening and universal testing and treatment for HIV in the HPTN 071 (PopART) community-randomised trial in Zambia and South Africa: A cross-sectional survey (TREATS)
Source: PLoS Med. 2023 Sep 8;20(9):e1004278. doi: 10.1371/journal.pmed.1004278 (PMC10490889; doi:10.1371/journal.pmed.1004278)
Supplement: S4 Table — (DOCX) [file pmed.1004278.s012.docx]

**S4 Table - TB prevalence by country, comparing individuals who self-reported they were HIV-positive and taking ART with HIV-negative individuals, overall and stratified by sex and age group**

|  | HIV-negative | | | HIV-positive, taking ART^1^ | | | Unadjusted odds ratio (OR), comparing HIV-positive individuals taking ART with HIV-negative individuals | Adjusted OR^2^, 95% CI | |
| --- | --- | --- | --- | --- | --- | --- | --- | --- | --- |
| **Zambia** | n^3^ | N^4^ | %^5^ | n^3^ | N^4^ | %^5^ | OR | OR | 95% CI |
| Male, age <30 | 28.0 | 5886 | **0.48** | 1.7 | 111 | **1.53** | 2.99 | 3.09 | [0.47,20.22] |
| Male, age 30-39 | 18.7 | 1590 | **1.17** | 9.6 | 268 | **3.58** | 3.12 | 3.26 | [1.43,7.44] |
| Male, age 40-49 | 20.0 | 877 | **2.28** | 11.4 | 381 | **3.00** | 1.32 | 1.35 | [0.64,2.87] |
| Male, age ≥50 | 9.4 | 1063 | **0.89** | 1.0 | 302 | **0.34** | 0.38 | 0.41 | [0.05,3.25] |
| Male, all ages | **76.1** | 9416 | **0.81** | **23.7** | 1062 | **2.24** | 2.80 | **1.76** | [1.04,2.97] |
|  |  |  |  |  |  |  |  |  | P=0.20^6^ |
| Female, age <30 | 9.6 | 8857 | **0.11** | 2.9 | 636 | **0.45** | 4.12 | 4.25 | [1.00,18.12] |
| Female, age 30-39 | 1.8 | 2579 | **0.07** | 9.6 | 1112 | **0.86** | 13.29 | 13.66 | [2.00,93.19] |
| Female, age 40-49 | 1.8 | 1163 | **0.16** | 5.2 | 922 | **0.57** | 3.78 | 3.98 | [0.58,27.24] |
| Female, age ≥50 | 6.8 | 1617 | **0.42** | 2.5 | 455 | **0.55** | 1.29 | 1.35 | [0.28,6.45] |
| Female, all ages | **20.0** | 14216 | **0.14** | **20.2** | 3125 | **0.65** | 4.62 | **4.13** | [2.00,8.54] |
|  |  |  |  |  |  |  |  |  | P=0.33^6^ |
| Males and females, all ages | **96.1** | **23632** | **0.41** | **43.9** | **4187** | **1.05** | 2.59 | **2.36** | **[1.55,3.57]** |
|  |  |  |  |  |  |  |  |  |  |
| **South Africa** |  |  |  |  |  |  |  |  |  |
| Male, age <30 | 44.8 | 3114 | **1.44** | 1.0 | 48 | **2.08** | 1.46 | 1.52 | [0.20,11.31] |
| Male, age 30-39 | 28.5 | 1181 | **2.41** | 2.4 | 158 | **1.53** | 0.58 | 0.63 | [0.12,3.36] |
| Male, age 40-49 | 31.1 | 675 | **4.60** | 4.6 | 154 | **2.97** | 0.63 | 0.71 | [0.24,2.05] |
| Male, age ≥50 | 31.1 | 944 | **3.29** | 5.8 | 115 | **5.06** | 1.55 | 1.83 | [0.69,4.84] |
| Male, all ages | **135.5** | 5914 | **2.29** | **13.8** | 475 | **2.91** | 1.27 | **1.01** | [0.54,1.89] |
|  |  |  |  |  |  |  |  |  | P=0.52^6^ |
| Female, age <30 | 28.8 | 3662 | **0.79** | 9.0 | 403 | **2.24** | 2.88 | 3.19 | [1.39,7.34] |
| Female, age 30-39 | 18.0 | 1468 | **1.22** | 10.9 | 786 | **1.39** | 1.13 | 1.36 | [0.58,3.17] |
| Female, age 40-49 | 3.6 | 867 | **0.42** | 7.5 | 518 | **1.44** | 3.60 | 4.46 | [1.03,19.38] |
| Female, age ≥50 | 12.6 | 1452 | **0.87** | 1.4 | 229 | **0.63** | 0.68 | 0.86 | [0.12,6.40] |
| Female, all ages | **63.0** | 7449 | **0.85** | **28.8** | 1936 | **1.49** | 1.77 | **2.12** | [1.23,3.66] |
|  |  |  |  |  |  |  |  |  | P=0.28^6^ |
| **Males and females, all ages** | **198.5** | **13363** | **1.49** | **42.6** | **2411** | **1.77** | 1.19 | **1.50** | **[1.01,2.23]** |

^1^ *Individuals who self-reported they were HIV-positive, and self-reported they were taking antiretroviral therapy (ART); ^2^ For sub-group analysis of males and females, adjusted for triplet and estimating age-group-specific odds ratios to compare HIV-positive with HIV-negative individuals (interaction between age group and HIV status, in the logistic regression model); for analysis of males and females combined, adjusted for triplet and allowing the association between age group and prevalent TB to be different for males and females (interaction between sex and age group, in the logistic regression model); ^3^ Number of individuals with prevalent TB; ^4^ Total participants; ^5^ Percentage with prevalent TB; ^6^ P-value for evidence for variation in ORs (comparing HIV-positive to HIV-negative individuals) among the 4 age groups*; *HIV=Human Immunodeficiency Virus; CI=confidence interval**; TB=tuberculosis.*
