# Supplementary material for: Tuberculosis prevalence after 4 years of population-wide systematic TB symptom screening and universal testing and treatment for HIV in the HPTN 071 (PopART) community-randomised trial in Zambia and South Africa: A cross-sectional survey (TREATS)
Source: PLoS Med. 2023 Sep 8;20(9):e1004278. doi: 10.1371/journal.pmed.1004278 (PMC10490889; doi:10.1371/journal.pmed.1004278)
Supplement: S5 Table — (DOCX) [file pmed.1004278.s013.docx]

**S5 Table TB prevalence by trial arm, sensitivity analyses**

|  | **Arm A** | | **Arm B** | | **Arm A+B** | | **Arm C** | |
| --- | --- | --- | --- | --- | --- | --- | --- | --- |
|  | n/N^1^ | %^2^ | n/N^1^ | %^2^ | n/N^1^ | %^2^ | n/N^1^ | %^2^ |
| **All individuals who were culture-eligible are assumed to have prevalent TB** |  |  |  |  |  |  |  |  |
| Overall (triplets 1-7 combined) | 179/13,906 | 1.29 | 156/11,787 | 1.33 | 335/25,693 | 1.30 | 315/23,863 | 1.32 |
| TB prevalence, geometric mean^3^ |  | **1.22** |  | **1.00** |  | **1.10** |  | **0.94** |
| Unadjusted Prevalence ratio (PR), [95% CI], p-value | 1.31 | [0.68,2.51]  p=0.38 | 1.05 | [0.55,2.01]  p=0.86 | 1.17 | [0.67,2.06]  p=0.54 | referent |  |
| Adjusted PR (aPR)^4^, [95% CI], p-value | **1.30** | **[0.69,2.45]**  **p=0.38** | **1.05** | **[0.56,1.97]**  **p=0.86** | **1.17** | **[0.68,2.02]**  **p=0.54** | referent |  |
|  |  |  |  |  |  |  |  |  |
| **All individuals who were sputum-eligible and who were given sputum containers but provided no sputum samples are assumed not to have prevalent TB** |  |  |  |  |  |  |  |  |
| Overall (triplets 1-7 combined) | 122/13,906 | 0.87 | 99/11,787 | 0.84 | 220/25,693 | 0.86 | 217/23,863 | 0.91 |
| TB prevalence, geometric mean^3^ |  | **0.86** |  | **0.68** |  | **0.76** |  | **0.67** |
| Unadjusted Prevalence ratio (PR), [95% CI], p-value | 1.28 | [0.69,2.38]  p=0.40 | 1.02 | [0.54,1.90]  p=0.96 | 1.14 | [0.67,1.95]  p=0.60 | referent |  |
| Adjusted PR (aPR)^4^, [95% CI], p-value | **1.26** | **[0.68,2.31]**  **p=0.42** | **1.01** | **[0.55,1.88]**  **p=0.96** | **1.13** | **[0.66,1.92]**  **p=0.62** | referent |  |
|  |  |  |  |  |  |  |  |  |
| **All individuals who were sputum-eligible but had missing Xpert-Ultra test results are assumed not to have prevalent TB** |  |  |  |  |  |  |  |  |
| Overall (triplets 1-7 combined) | 112/13,906 | 0.81 | 90/11,787 | 0.77 | 202/25,693 | 0.79 | 199/23,863 | 0.83 |
| TB prevalence, geometric mean^3^ |  | **0.79** |  | **0.62** |  | **0.70** |  | **0.62** |
| Unadjusted Prevalence ratio (PR), [95% CI], p-value | 1.28 | [0.69,2.36]  p=0.40 | 1.00 | [0.54,1.87]  p=0.99 | 1.13 | [0.66,1.93]  p=0.62 | referent |  |
| Adjusted PR (aPR)^4^, [95% CI], p-value | **1.24** | **[0.68,2.29]**  **p=0.44** | **1.00** | **[0.54,1.84]**  **p=0.99** | **1.11** | **[0.66,1.89]**  **p=0.66** | referent |  |

*^1^ Number of individuals with prevalent TB (rounded to nearest whole number) / total participants. Number of individuals with prevalent TB is calculated after multiple imputation of missing data on prevalent TB status, and is an average across 300 imputed datasets - hence not a whole number; ^2^ Percentage with prevalent TB; ^3^ Geometric mean of TB prevalence across 7 communities;^4^ adjusted triplet, sex, age group, HIV status*; *TB= Tuberculosis; HIV=Human Immunodeficiency Virus; CI=confidence interval*
