# Supplementary material for: Tuberculosis prevalence after 4 years of population-wide systematic TB symptom screening and universal testing and treatment for HIV in the HPTN 071 (PopART) community-randomised trial in Zambia and South Africa: A cross-sectional survey (TREATS)
Source: PLoS Med. 2023 Sep 8;20(9):e1004278. doi: 10.1371/journal.pmed.1004278 (PMC10490889; doi:10.1371/journal.pmed.1004278)
Supplement: S1 Consort Checklist — Extension of CONSORT for abstracts to reports of cluster-randomised trials. (PDF) [file pmed.1004278.s014.pdf]

S1 CONSORT 2010 checklist of information to include when reporting a cluster randomised trial

Section/Topic	Item No	Standard Checklist item	Extension for cluster designs	section/paragraph'*
**Title and abstract**				
	1a	Identification as a randomised trial in the title	Identification as a cluster randomised trial in the title	1 (community as cluster..)
	1b	Structured summary of trial design, methods, results, and conclusions (for specific guidance see CONSORT for abstracts)[i,ii]	See table 2	Abstract
**Introduction**				
**Background and objectives**	2a	Scientific background and explanation of rationale	Rationale for using a cluster design	Introduction section, 3rd paragraph
	2b	Specific objectives or hypotheses	Whether objectives pertain to the cluster level, the individual participant level or both	Introduction section, 4th paragraph (population-level)
**Methods**				
**Trial design**	3a	Description of trial design (such as parallel, factorial) including allocation ratio	Definition of cluster and description of how the design features apply to the clusters	Methods- section "HPTN 071 and TREATS trial design" first paragraph
	3b	Important changes to methods after trial commencement (such as eligibility criteria), with reasons		N.A.
**Participants**	4a	Eligibility criteria for participants	Eligibility criteria for clusters	Participants: Methods TREATS TB prevalence survey design – section Field procedures; Clusters: Methods HPTN 071 and TREATS trial design
	4b	Settings and locations where the data were collected		
**Interventions**	5	The interventions for each group with sufficient details to allow replication, including how and when they were actually administered	Whether interventions pertain to the cluster level, the individual participant level or both	Methods – section HPTN 071 and TREATS trial design
**Outcomes**	6a	Completely defined pre-specified primary and secondary outcome measures, including how and when they were assessed	Whether outcome measures pertain to the cluster level, the individual participant level or both	Methods- section HPTN 071 and TREATS trial design – 3rd paragraph
	6b	Any changes to trial outcomes after the trial commenced, with reasons		N.A.
**Sample size**	7a	How sample size was determined	Method of calculation, number of clusters(s) (and whether equal or unequal cluster sizes are assumed), cluster size, a coefficient of intracluster	Methods- section HPTN 071 and TREATS trial design – 1st paragraph

			correlation (ICC or *k*), and an indication of its uncertainty	
	7b	When applicable, explanation of any interim analyses and stopping guidelines		N.A.
**Randomisation:**				
**Sequence generation**	8a	Method used to generate the random allocation sequence		Methods HPTN 071 and TREATS trial design, and PopART intervention
	8b	Type of randomisation; details of any restriction (such as blocking and block size)	Details of stratification or matching if used	Methods- section HPTN 071 and TREATS trial design, and PopART intervention – 1st paragraph and TREATS TB prevalence survey design - Sample size and survey timing – section Field procedures
**Allocation concealment mechanism**	9	Mechanism used to implement the random allocation sequence (such as sequentially numbered containers), describing any steps taken to conceal the sequence until interventions were assigned	Specification that allocation was based on clusters rather than individuals and whether allocation concealment (if any) was at the cluster level, the individual participant level or both	Methods- section HPTN 071 and TREATS trial design, and PopART intervention – 1st paragraph
**Implementation**	10	Who generated the random allocation sequence, who enrolled participants, and who assigned participants to interventions	Replace by 10a, 10b and 10c	
	10a		Who generated the random allocation sequence, who enrolled clusters, and who assigned clusters to interventions	Methods- section HPTN 071 and TREATS trial design, and PopART intervention – 1st paragraph
	10b		Mechanism by which individual participants were included in clusters for the purposes of the trial (such as complete enumeration, random sampling)	Methods- section – Field procedures
	10c		From whom consent was sought (representatives of the cluster, or individual cluster members, or both), and whether consent was sought before or after randomisation	TREATS TB prevalence survey design - Sample size and survey timing – section Field procedures
**Blinding**	11a	If done, who was blinded after assignment to interventions (for example, participants, care providers, those assessing outcomes) and how		**N.A.**
	11b	If relevant, description of the similarity of interventions		Methods- section HPTN 071 and TREATS trial design, and PopART intervention – 1st paragraph

**Statistical methods**	12a	Statistical methods used to compare groups for primary and secondary outcomes	How clustering was taken into account	
	12b	Methods for additional analyses, such as subgroup analyses and adjusted analyses		TREATS TB prevalence survey design - Sample size and survey timing – section – section Data capture and statistical analysis- 3rd paragraph
**Results**				
**Participant flow (a diagram is strongly recommended)**	13a	For each group, the numbers of participants who were randomly assigned, received intended treatment, and were analysed for the primary outcome	For each group, the numbers of clusters that were randomly assigned, received intended treatment, and were analysed for the primary outcome	Figure 1
	13b	For each group, losses and exclusions after randomisation, together with reasons	For each group, losses and exclusions for both clusters and individual cluster members	Figure 1
**Recruitment**	14a	Dates defining the periods of recruitment and follow-up		Figure 1
	14b	Why the trial ended or was stopped		N.A.
**Baseline data**	15	A table showing baseline demographic and clinical characteristics for each group	Baseline characteristics for the individual and cluster levels as applicable for each group	Table 1
**Numbers analysed**	16	For each group, number of participants (denominator) included in each analysis and whether the analysis was by original assigned groups	For each group, number of clusters included in each analysis	Figure 1/Table 1
**Outcomes and estimation**	17a	For each primary and secondary outcome, results for each group, and the estimated effect size and its precision (such as 95% confidence interval)	Results at the individual or cluster level as applicable and a coefficient of intracluster correlation (ICC or k) for each primary outcome	Table 2
	17b	For binary outcomes, presentation of both absolute and relative effect sizes is recommended		Table 2
**Ancillary analyses**	18	Results of any other analyses performed, including subgroup analyses and adjusted analyses, distinguishing pre-specified from exploratory		Table 3, Supplement tables S3-10
**Harms**	19	All important harms or unintended effects in each group (for specific guidance see CONSORT for harms[iii])		N.A.

**Discussion**				
**Limitations**	20	Trial limitations, addressing sources of potential bias, imprecision, and, if relevant, multiplicity of analyses		Discussion – section Generalisability, strengths and limitations
**Generalisability**	21	Generalisability (external validity, applicability) of the trial findings	Generalisability to clusters and/or individual participants (as relevant)	Discussion – section Generalisability, strengths and limitations
**Interpretation**	22	Interpretation consistent with results, balancing benefits and harms, and considering other relevant evidence		Discussion – section Generalisability, strengths and limitations and Implications of study findings
**Other information**				
**Registration**	23	Registration number and name of trial registry		Methods section - Ethical considerations and trial registration
**Protocol**	24	Where the full trial protocol can be accessed, if available		ref 21
**Funding**	25	Sources of funding and other support (such as supply of drugs), role of funders		Funding statement

** Note: page numbers optional depending on journal requirements*

Extension of CONSORT for abstracts to reports of cluster randomised trials

Item	Standard Checklist item	Extension for cluster trials
**Title**	Identification of study as randomised	**Identification of study as cluster randomised**
**Trial design**	Description of the trial design (e.g. parallel, cluster, non-inferiority)	
**Methods**		
**Participants**	Eligibility criteria for participants and the settings where the data were collected	**Eligibility criteria for clusters**
**Interventions**	Interventions intended for each group	
**Objective**	Specific objective or hypothesis	**Whether objective or hypothesis pertains to the cluster level, the individual participant level or both**
**Outcome**	Clearly defined primary outcome for this report	**Whether the primary outcome pertains to the cluster level, the individual participant level or both**
**Randomization**	How participants were allocated to interventions	**How clusters were allocated to interventions**
**Blinding (masking)**	Whether or not participants, care givers, and those assessing the outcomes were blinded to group assignment	
**Results**		
**Numbers randomized**	Number of participants randomized to each group	**Number of clusters randomized to each group**
**Recruitment**	Trial status[1]	
**Numbers analysed**	Number of participants analysed in each group	**Number of clusters analysed in each group**
**Outcome**	For the primary outcome, a result for each group and the estimated effect size and its precision	**Results at the cluster or individual participant level as applicable for each primary outcome**
**Harms**	Important adverse events or side effects	
**Conclusions**	General interpretation of the results	
**Trial registration**	Registration number and name of trial register	
**Funding**	Source of funding	

REFERENCES

i Hopewell S, Clarke M, Moher D, Wager E, Middleton P, Altman DG, et al. CONSORT for reporting randomised trials in journal and conference abstracts. *Lancet* 2008, 371:281-283

ii Hopewell S, Clarke M, Moher D, Wager E, Middleton P, Altman DG at al. (2008) CONSORT for reporting randomized controlled trials in journal and conference abstracts: explanation and elaboration. *PLoS Med* 5(1): e20

iii Ioannidis JP, Evans SJ, Gotzsche PC, O'Neill RT, Altman DG, Schulz K, et al.. Better reporting of harms in randomized trials: an extension of the CONSORT statement. *Ann Intern Med* 2004; 141(10):781-788.

CONSERVE Checklists

Use CONSERVE-CONSORT for completed trial reports and CONSERVE-SPIRIT for trial protocols.

[1] Relevant to Conference Abstracts

CONSERVE-CONSORT Extension: [DATE]					
**Item**	**Item Title**	**Description**			**section/paragraph**
I.	Extenuating Circumstances	Describe the circumstances and how they constitute extenuating circumstances.			Figure 1
II.	Important Modifications	a. Describe how the modifications are important modifications.			Supplement – protocol amendment
		b. Describe the impacts and mitigating strategies, including their rationale and implications for the trial.			Supplement – protocol amendment
		c. Provide a modification timeline.			Supplement – protocol amendment
III.	Responsible Parties	State who planned, reviewed and approved the modifications.<br>funder approved plus relevant ethics bodies			Supplement – protocol amendment –
IV.	Interim data	If modifications were informed by trial data, describe how the interim data were used, including whether they were examined by study group, and whether the individuals reviewing the data were blinded to the treatment allocation.			N.A.
**CONSORT Number and Item**		For each row, if important modifications occurred check “direct impact” and/or “mitigating strategy” and describe the changes in the trial manuscript or supplement. Check “no change” for items that are unaffected in the extenuating circumstance.			**Page No.**
		**No Change**	**Impact***	**Mitigating Strategy****	
1	Title and abstract	X			
2	Introduction	X			
3	Methods: Trial Design	X			
4	Methods: Participants	X			
5	Methods: Interventions	X			
6	Methods: Outcomes	x			

7	Methods: Sample Size			X Reduction in sample size was modest (around 10-15%)in some communities to ensure study power was reduced by only a few percent compared to the original design.	Supplement – protocol amendment
8-10	Methods: Randomisation	X			
11	Methods: Blinding	X			
12	Methods: Statistical methods	X			
13	Results: Participant flow	X			
14	Results: Recruitment	X			
15	Results: Baseline data	X			
16	Results: Numbers analysed	X			
	Results: Outcomes and estimation	X			
18	Results: Ancillary analyses	X			
19	Results: Harms	X			
20	Discussion: Limitations		X		Discussion – section Interpretation of findings 4th paragraph
21	Discussion: Generalisability	X			
	Other information: Registration	X			
24	Other information: Protocol		X		Supplement – protocol amendment
25	Other information: Funding			X	No cost extension

*Aspects of the trial that are directly affected or changed by the extenuating circumstance and are not under the control of investigators, sponsor or funder.
**Aspects of the trial that are modified by the study investigators, sponsor or funder to respond to the extenuating circumstance or manage the direct impacts on the trial.
