# Supplementary material for: Tuberculosis prevalence after 4 years of population-wide systematic TB symptom screening and universal testing and treatment for HIV in the HPTN 071 (PopART) community-randomised trial in Zambia and South Africa: A cross-sectional survey (TREATS)
Source: PLoS Med. 2023 Sep 8;20(9):e1004278. doi: 10.1371/journal.pmed.1004278 (PMC10490889; doi:10.1371/journal.pmed.1004278)
Supplement: S1 Protocol — (PDF) [file pmed.1004278.s015.pdf]

Tuberculosis Reduction through Expanded Anti-Retroviral Treatment and Screening (TREATS) Project

Sponsored by:
London School of Hygiene and Tropical Medicine

Funded by:
European and Developing Countries Clinical Trials Partnership (EDCTP)

Overall PI:
Helen Ayles
London School of Hygiene & Tropical Medicine
London, United Kingdom

Version 4.2
11th June 2020

TREATS

TABLE OF CONTENTS

LIST OF TABLES AND FIGURES 4
LIST OF ABBREVIATIONS AND ACRONYMS 5
INVESTIGATORS 6
INVESTIGATOR SIGNATURE PAGE 9
SUMMARY 10
1.0 INTRODUCTION 14
1.1 Background and Prior Research 14
1.2 Rationale 14
2.0 STUDY OBJECTIVES AND DESIGN 15
2.1 Objectives 15
2.2 Study Design 17
2.3 Timing of Intervention and Research Components 17
3.0 STUDY INTERVENTION 20
3.1 Intervention 20
3.2 Standard of Care 20
4.0 STUDY POPULATION 23
4.1 Description/Selection of the 14 Study Communities 23
4.2 Community Engagement 25
5.0 RESEARCH PROCEDURES AND ACTIVITIES 27
5.1 Infection Cohort 27
5.1.1 Sampling/Recruitment of Infection Cohort 27
5.1.2 Procedures and Activities 28
5.1.3 Retention and Losses to Follow-up 30
5.2 Prevalence Survey 30
5.2.1 Sampling/Recruitment of Prevalence Survey Participants 30
5.2.2 Procedures and Activities 31
5.3 Implementation Science Studies 33
5.3.1 Secondary data Analysis 34
5.3.2 Qualitative Studies 35
6.0 SAFETY MONITORING AND SOCIAL HARM REPORTING 36
6.1 Safety Monitoring 36
7.0 STATISTICAL CONSIDERATIONS AND DATA ANALYSIS 37
7.1 Sample Size 37
7.1.1 Mathematical Modelling to inform Sample Size Calculations 37
7.1.2 Sample size for Primary Endpoints 38
7.2 Data management 40
7.3 Statistical Analysis 42
7.4 Matematical modelling 43
7.5 Cost-effectiveness Analysis (CEA). 45
7.5.1 CEA data. 45

8.0 HUMAN SUBJECTS CONSIDERATIONS 47
8.1 Collaborative Partnerships 47
8.2 Social Value 47
8.3 Scientific Validity 47
8.4 Compliance with ethical standards 48
8.5 Fair Subject Selection 48
8.6 Risk-Benefit Assessment 49
8.6.1 Procedures that may pose a risk to individuals and mitigation of risk 50
8.7 Informed Consent 52
8.8 Independent Ethical Review 53
8.9 Respect for Participants and Communities During and After the Study 54
8.9.1 Confidentiality 54
8.9.2 Study Monitoring 54
8.9.3 Dissemination of results 55
9.0 LABORATORY SPECIMENS AND BIOHAZARD CONTAINMENT 55
9.1 Local Laboratory Specimens 55
9.2 Quality Control and Quality Assurance Procedures 55
9.3 Specimen Storage and Possible Future Research Testing 55
10.0 ADMINISTRATIVE PROCEDURES 56
10.1 Project Management 56
10.2 Quality Management 56
10.3 Investigator's Records 56
10.4 Use of Information and Publications 56
11.0 REFERENCES 57
12.0 APPENDICES 58
APPENDIX I- SAMPLE INFORMED CONSENT FORM – INFECTION COHORT 59
APPENDIX II - SAMPLE INFORMED CONSENT FORM – PREVALENCE SURVEY 80
APPENDIX III - SAMPLE INFORMED CONSENT FORM – QUALITATIVE STUDIES PARTICIPANTS 101

LIST OF TABLES AND FIGURES

List of Tables and Figures

Figure 1: OVERVIEW OF STUDY DESIGN AND relationship to HPTN 071 13
Figure 2- Timing of Deployment of Intervention and Research Components of HPTN071 and TREATS 19
Table 1- Summary of Intervention Components 21
Table 2- Fourteen study clusters in Zambia and South Africa and relevant baseline data collected from a randomly selected cohort of 18-44-year old's (HPTN071 PC0 2014) 24
Figure 3- Location of 14 clusters in Zambia and South Africa 25
Figure 4 Screening and diagnostic algorithm for prevalence survey participants 32
Table 3: The modelled effect of the HPTN071 (PopART) interventions on TB disease prevalence and the incidence of TB infection 38
Table 4: Summary table of data sources 45

LIST OF ABBREVIATIONS AND ACRONYMS

AE	Adverse Event
AIDS	Acquired Immunodeficiency Syndrome
ART	Anti-Retroviral Therapy
ARV	Anti-Retroviral
CEA	Cost-effectiveness analysis
DALY	Disability-Adjusted Life-Year
DSMB	Data Safety Monitoring Board
EC	Ethics Committee
EQA	External Quality Assurance
GCLP	Good Clinical Laboratory Practice
GCP	Good Clinical Practice
HCF	Health Care Facility
HCT	HIV Counselling and Testing
HIV	Human Immunodeficiency Virus
HPTN	HIV Prevention Trials Network
HPTN071	HIV Prevention Trials Network study 071 (PopART)
ICF	Informed Consent Forms
IRB	Institutional Review Board
LDMS	Laboratory Data Management System
MTB	*Mycobacterium tuberculosis*
MFS	Mobile Field Site
MSM	Men who have Sex with Men
NB	Nota Bene
PopART	Population effects of antiretroviral therapy to reduce transmission of HIV
QA	Quality Assurance
QALY	Quality-Adjusted Life-Year
QC	Quality Control
RR	Rate Ratio
SAE	Serious Adverse Event
SANAC	South African National AIDS Council
TB	Tuberculosis
UK	United Kingdom
UNAIDS	United Nations Programme on HIV/AIDS
US	United States (of America)
UTT	Universal Testing and Treatment
VCT	Voluntary Counselling and Testing
WHO	World Health Organization
ZAMBART	Zambia AIDS Related Tuberculosis Project
ZAMSTAR	Zambia-South Africa TB and AIDS Reduction Program

INVESTIGATORS

Helen Ayles
Clinical Scientist/Overall Principal Investigator
Dept. of Clinical Research
London School of Hygiene and Tropical Medicine
Keppel Street
London WC1E 7HT, UK
Zambart,
University of Zambia,
School of Medicine, Ridgeway Campus
P.O. Box 50697, Lusaka, Zambia

Virginia Bond
Social Scientist
Dept. of Global Health and Development, London School of Hygiene & Tropical Medicine,
Tavistock Place, London WCIH 9SH, UK
Zambart, University of Zambia, School of Medicine
P.O. Box 50697
Lusaka, Zambia

Petra De Haas
Laboratory Consultant
KNCV Tuberculosis Foundation
Benoordenhoutseweg 46 - 2596 BC The Hague - The Netherlands

Pete Dodd
Mathematical modeller
School of Health and Related Research
University of Sheffield
30 Regent Street, Sheffield, S1 4DA, UK

Linda Mureithi
Clinical Scientist PI South Africa
Health Systems Trust
1st Floor, Block B,Aintree Park
Doncaster Road,Kenilworth
7700
Cape Town,8000
South Africa

Sarah Fidler
Clinical Scientist
Imperial College London
Professor in Communicable Diseases
St Mary's Campus

HIV Clinical Trials Unit
Winston Churchill Wing
London W2 1NY, UK

Sian Floyd
Lead Statistician
Department of Infectious Disease Epidemiology
London School of Hygiene & Tropical Medicine
Keppel Street
London, WC1E 7HT, UK

Richard Hayes
Statistical Epidemiologist
Department of Infectious Disease Epidemiology
London School of Hygiene & Tropical Medicine
Keppel Street
London WC1E 7HT, UK

Nico Kalisvaart
Surveillance and data management consultant
KNCV Tuberculosis Foundation
Benoordenhoutseweg 46 - 2596 BC The Hague - The Netherlands

Eveline Klinkenberg
Senior Epidemiologist
KNCV Tuberculosis Foundation
Benoordenhoutseweg 46 - 2596 BC The Hague - The Netherlands

Barry Kosloff
Laboratory Manager
London School of Hygiene & Tropical Medicine, Keppel Street
London WC1E 7HT, UK
Zambart,
University of Zambia,
School of Medicine, Ridgeway Campus
P.O. Box 50697, Lusaka, Zambia

Alwyn Mwinga
Clinical Scientist
Zambart,
University of Zambia,
School of Medicine, Ridgeway Campus
P.O. Box 50697, Lusaka, Zambia

Ab Schaap
Data-analyst/Statistician Zambia
London School of Hygiene & Tropical Medicine.
Keppel Street
London WC1E 7HT, UK
Zambart, University of Zambia,
School of Medicine, Ridgeway Campus
P.O. Box 50697, Lusaka, Zambia

Kwame Shanaube
PI - Zambia
Zambart,
University of Zambia,
School of Medicine, Ridgeway Campus
P.O. Box 50697, Lusaka, Zambia

Lily Telisinghe
Clinical Epidemiologist
Department of Clinical Research
London School of Hygiene and Tropical Medicine
Keppel Street
London WC1E 7HT, UK

Modupe Amofa- Sekyi
Clinical Scientist
Zambart,
University of Zambia,
School of Medicine, Ridgeway Campus
P.O. Box 50697, Lusaka, Zambia
Phone : +260-211-257215

Ranjeeta Thomas
Economist,
Department of Health Policy
London School of Economics and Political Science
Houghton Street
London WC2A 2AE, UK
UK

Musonda Simwinga
Community Engagement Lead
Zambart,
University of Zambia,
School of Medicine, Ridgeway Campus
P.O. Box 50697, Lusaka, Zambia

Tuberculosis Reduction through expanded Anti-Retroviral Treatment and Screening project TREATS

Final Version 4.2
11/06/2020

INVESTIGATOR SIGNATURE PAGE

Sponsored by:
London School of Hygiene and Tropical medicine

Funded by:
EDCTP

I, the Investigator of Record, agree to conduct this study in full accordance with the provisions of this protocol. I agree to maintain all study documentation for a minimum of three years after submission of the site's final Financial Status Report.

I have read and understand the information in this protocol and will ensure that all associates, colleagues, and employees assisting in the conduct of the study are informed about the obligations incurred by their contribution to the study.

____
Name of Investigator of Record

__________________________________ __________________________________
Signature of Investigator of Record Date

Tuberculosis Reduction through expanded Anti-Retroviral Treatment and Screening project TREATS

SUMMARY

Tuberculosis (TB) has overtaken HIV as the leading infectious cause of death worldwide and requires a major policy shift for it to be controlled in line with the WHO Stop-TB goal to "end TB" [1]. However, how to control TB at population level in the context of HIV, is unknown. Some of the best evidence to date comes from the Southern African ZAMSTAR trial, where a household-level TB /HIV intervention including TB symptom screening, HIV counselling and testing with linkage to care and isoniazid preventive therapy (IPT) as indicated, was offered to all household members of TB patients [2], Despite only reaching ~6% of households in the intervention communities, the data showed a nearly 20% reduction in TB disease prevalence and 50% reduction in TB infection incidence at the population-level, although the effects were of borderline statistical significance. Increasing the scope of the intervention to all households and thus all community members, may therefore significantly change the burden of TB.

The proposed TREATS project builds on the experience of ZAMSTAR and is nested within the ongoing HPTN 071 (PopART) trial in Zambia and South Africa. This project will evaluate the effect of a combination TB/HIV prevention intervention implemented in the PopART trial to address the question of whether population level screening for tuberculosis, combined with universal testing and treatment (UTT) for HIV can significantly reduce the prevalence of TB and "End TB"[1].

The overall aim of this project is to measure the impact of a combined TB/HIV intervention of population level screening for TB, combined with universal testing and treatment (UTT) for HIV, delivered over 4 years, on notified TB incidence, prevalence of TB disease and incidence of TB infection.

Objectives:

1. To compare the population impact of a combined TB/HIV intervention of population level screening for tuberculosis, combined with universal testing and treatment (UTT) for HIV on:
    a. The prevalence of TB disease in a randomly selected sample of individuals aged 15 years and above
    b. The incidence of infection with TB in a randomly selected cohort of adolescents and young adults (aged 15-24 years)
    c. The effect on notified bacteriologically confirmed pulmonary TB incidence in the parent HPTN071 trial Population Cohort participants aged 18-44 years over the last 24 months of follow-up (2017-2018) through linkage of cohort data to routine TB case notification data
    d. The effect on bacteriologically confirmed pulmonary TB case notification rates among adults (≥18 years) residing in the study communities, during the period 2017-2018, using routine TB case notification data
    e. The clinical characteristics and treatment outcomes of bacteriologically confirmed pulmonary TB cases in Population Cohort participants aged 18-44 years over 36

months through linkage of cohort data to routine TB case notification data

f. The clinical characteristics and treatment outcomes of bacteriologically confirmed pulmonary TB cases among adults (≥18 years) residing in the study communities over 60 months, using routine TB case notification data

2. To understand the pathway to impact of the PopART combined TB/HIV intervention on TB by measuring the yield of case finding by HIV status, change in TB notifications at health facility level and self-reported TB incidence.
3. To use qualitative methods to assess the delivery of the PopART interventions:
   a. describing TB stigma and popular understanding of TB among community members drawing on longitudinal data (2004-2018) from these selected communities
   b. describing the experience of diagnosed TB patients and their households across intervention and control sites, and household intervention and standard of care health facility services
4. To use mathematical modelling to assess:
   a. The effect of the different PopART intervention components (universal ART and screening for TB) on the impact on TB
   b. The projected impact of this intervention on TB in other settings and what factors influence this
5. Use economic analysis methods combined with mathematical modelling to measure the cost-effectiveness of the PopART intervention in terms of its impact on TB
6. To evaluate newer methods for measuring the impact of public health interventions on TB
   a. To evaluate the role of QuantiFERON Gold Plus in assessing epidemiological impact of TB interventions on infection with TB
   b. To evaluate new biomarkers for assessing TB infection and disease as well as predictors of progression from infection with *M. tuberculosis* to active disease ("incipient TB")
   c. To compare a novel method of measuring the prevalence of active TB disease, including Xpert MTB/RIF Ultra combined with computer aided digital chest x-ray (CAD4TB), with traditional methods in terms of costs and efficiency
7. To increase the capacity of African field sites to conduct large cluster randomised trials through south-south as well as north-south collaboration
8. To inform global policy:
   a. On the costs and effectiveness of the PopART intervention to control TB
   b. On strategies to measure the burden of TB at population level
9. To raise awareness amongst researchers, funders, programme managers and civil society of the impact of a combined TB/HIV intervention of population level screening for TB and universal HIV testing and treatment to inform future approaches to addressing TB effectively

Study Sites: The study is expected to be implemented in the communities identified below.

- The study communities in Zambia are spread across 4 provinces and 6 districts. Each community is the catchment population of one or two government health facilities.
    - Ndeke and Chimwemwe in Kitwe District (Copperbelt Province)
    - Chipulukusu and Chifubu in Ndola District (Copperbelt Province)
    - Makululu, Ngungu & Bwacha in Kabwe District (Central Province)

- Chawama, Chipata and Kanyama in Lusaka District (Lusaka Province)
    - Maramba and Dambwa in Livingstone District (Southern Province)
    - Shampande in Choma District (Southern Province)
- The study communities in South Africa are located in the Cape Metro District and Cape Winelands District of the Western Cape Province. As above, the communities are defined by the catchment population of one or two government health facilities.
    - Delft South (Metro District)
    - Kuyasa (Metro District)
    - Wellington (Cape Winelands District)
    - Luvuyo (Metro District)
    - Town II (Metro District)
    - Ikhwezi (Metro District)
    - Bloekombos (Metro District)
    - Dalevale (Cape Winelands District)
    - Cloetesville & Idas Valley(Cape Winelands District)

Figure 1: OVERVIEW OF STUDY DESIGN AND relationship to HPTN 071

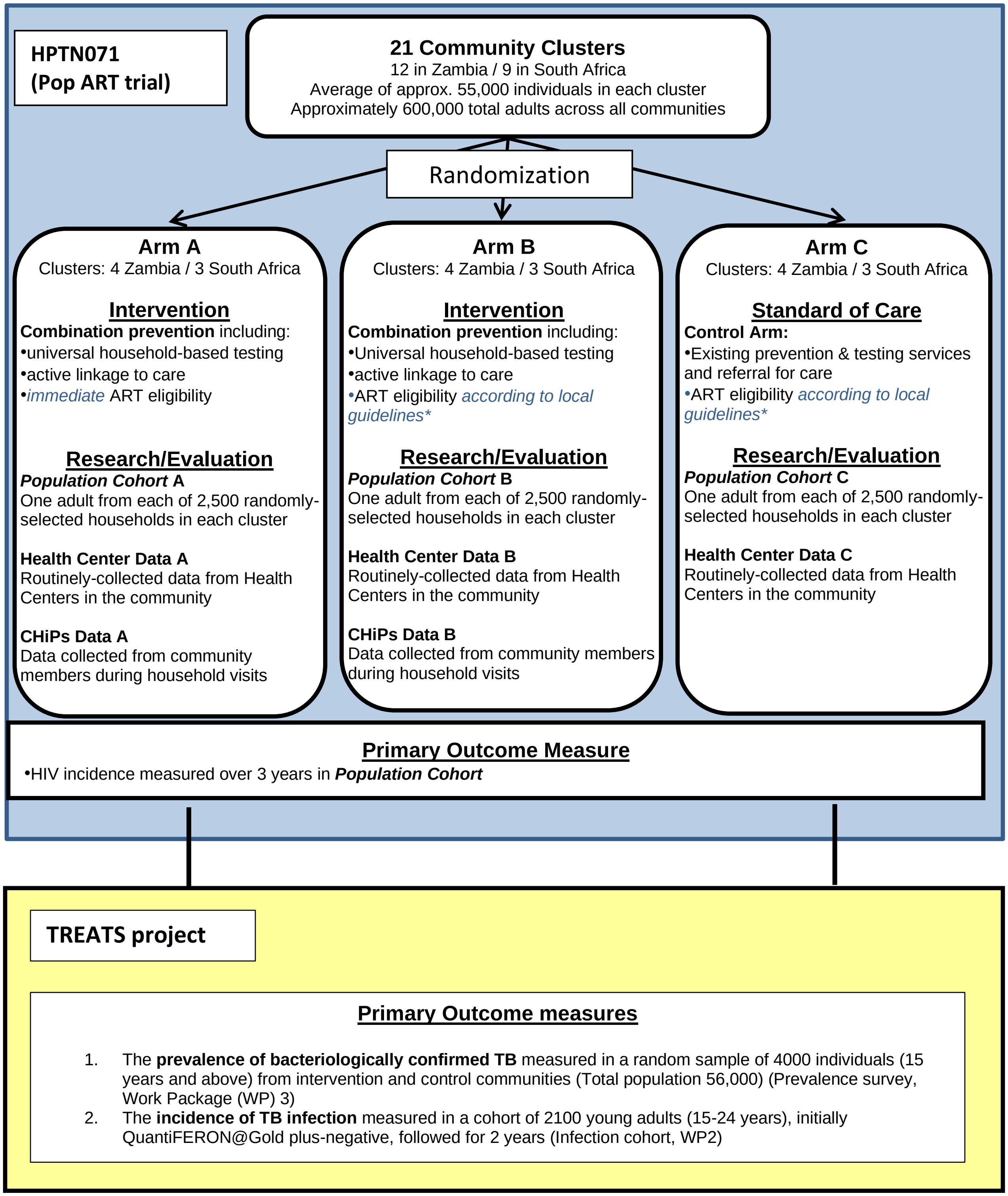

1.0 INTRODUCTION

1.1 Background and Prior Research

Tuberculosis (TB) and HIV are the leading infectious causes of death worldwide with 1.8 million people dying of TB in 2015[3]. For people living with HIV (PLWH) TB continues to be the most significant co-infection with 1.2 million PLWH developing TB in 2015 and 0.4 million dying of the disease. TB continues to disproportionately affect the poorest and most vulnerable individuals in society but despite ambitious targets to "End TB" [4] no significant progress in those regions most highly affected by the joint TB/HIV epidemic has yet been observed. The World Health Organization (WHO) recommends strategies to control TB/HIV co-infection but implementation of these activities needs to be accelerated to reach these targets. It is estimated that 4.3 million people with TB were missed (i.e. not diagnosed or treated) in 2015 largely due to health system failures to identify people with symptoms of TB and accurately diagnose them[3].

Several approaches to TB control have been studied and the concept of widespread screening for active disease, abandoned as a public health strategy in the 1970's, has now been re-visited.[5] WHO has recently published guidelines on screening for active TB but robust evidence is lacking about how best to screen (with what combination of TB symptoms and chest X-ray, for example) at population level and how best to identify at-risk groups.[6] Screening will only control TB if it identifies individuals early enough in the course of their disease such that onward transmission of infection is reduced. The concept of TB screening at population level was tested in the Zambia South Africa TB and AIDS reduction (ZAMSTAR) trial, a large community randomised trial of interventions to reduce tuberculosis at population level. ZAMSTAR failed to significantly reduce TB prevalence or transmission through enhancing screening and case finding for TB at the community level. However, in the same study, an approach based on integrated TB-HIV services delivered to the households of TB patients showed some evidence of effect.[2] The same household intervention did not significantly reduce TB stigma[7].

Treating HIV earlier could also significantly reduce TB in those regions most highly affected by the joint TB and HIV epidemics[8]. The increased availability of effective antiretroviral therapy (ART) and the promise that universal treatment, along with a combination HIV prevention approach, could reduce the transmission of HIV has led to renewed efforts to test and treat all HIV+ individuals and limit HIV transmission at population level. The US-funded HIV Prevention Trials Network (HPTN) 071 (PopART) trial (NCT01900977) is an ambitious cluster-randomised trial of a combination HIV prevention package including universal HIV testing and treatment (UTT) to reduce the transmission of HIV at population level. This trial is being conducted in 21 communities with an overall population of approximately 1 million people in Zambia and the Western Cape Province of South Africa[9]. The trial is the largest trial of a combination HIV prevention intervention and is unique in that it also includes scale up of recommended TB/HIV combination prevention as well as community-wide screening for TB through a TB symptom checklist with workup for those who screen positive in the survey.

1.2 Rationale

The hypothesis behind this project is that to control TB associated with HIV there are two main strategies available:

1. **Reduction in the transmission of TB by earlier detection and treatment of infectious cases.** Research to demonstrate that this strategy works has provided mixed results; the ZAMSTAR study showed no reduction in transmission when community level enhancement of case finding was implemented to the whole community (although a household intervention showed some evidence of effect)[2]. However, a study in Zimbabwe did show a reduction in the prevalence of TB when measured after two different TB case finding interventions[10].
2. **Reduction in TB incidence among HIV-infected individuals.** For HIV infected individuals, ART has been shown conclusively to reduce individual risk of development of TB[11]. However, longer survival, with a residual increased risk of TB compared with HIV-negative individuals, means that the population-level impact of ART provision on TB disease and transmission is less clear[12,13]. TB preventive therapy is also recognised to have an important effect at individual level[14], but challenges for wide-scale implementation mean that it has not been demonstrated to have a population-level effect[15,16].

The HPTN071(PopART) study is currently the only randomised trial where both strategies are being implemented together at population level and where this hypothesis can be tested.

A mathematical model developed for this study, using the best available epidemiological data, predicts that a combination HIV/TB prevention strategy combining TB and HIV screening, universal HIV treatment and treatment of TB cases identified could halve the prevalence of TB disease and also the incidence of infection (transmission)of TB after 4 years of implementation.

Measuring the impact of HIV/TB combination prevention interventions on TB at population level requires substantial investment to obtain the highest quality data, on which study validity depends. TB notification and other routinely available TB-related data are limited by health system weaknesses, and comparisons can be biased because of variation among clinics in diagnostic approaches and also because active case finding is expected to increase case notifications initially, only subsequently followed by a decline, and this can distort measurement. Traditional approaches for measuring the burden of TB disease, such as prevalence surveys based on TB culture, and transmission, such as tuberculin skin test surveys, have been logistically challenging and expensive. However, with improved diagnostic tools for measuring TB infection and disease the measurement of these outcomes has become more accurate and feasible.

2.0 STUDY OBJECTIVES AND DESIGN

The overall aim of this project is to measure the impact of a combined TB/HIV intervention of population level screening for TB, combined with universal testing and treatment (UTT) for HIV, delivered over 4 years, on TB incidence, prevalence and incidence of TB infection.

2.1 Objectives

1. To compare the population impact of a combined TB/HIV intervention of population level screening for tuberculosis, combined with universal testing and treatment (UTT) for HIV on:

a. The prevalence of TB disease in a randomly selected sample of individuals aged 15 years and above
b. The incidence of infection with TB in a randomly selected cohort of adolescents and young adults (aged 15-24 years)
c. The effect on notified bacteriologically confirmed pulmonary TB incidence in the parent HPTN071 trial Population Cohort participants aged 18-44 years over the last 24 months of follow-up (2017-2018) through linkage of cohort data to routine TB case notification data
d. The effect on bacteriologically confirmed pulmonary TB case notification rates among adults (≥18 years) residing in the study communities, during the period 2017-2018, using routine TB case notification data
e. The clinical characteristics and treatment outcomes of bacteriologically confirmed pulmonary TB cases in Population Cohort participants aged 18-44 years over 36 months through linkage of cohort data to routine TB case notification data
f. The clinical characteristics and treatment outcomes of bacteriologically confirmed pulmonary TB cases among adults (≥18 years) residing in the study communities over 60 months (2014-2018), using routine TB case notification data

2. To understand the pathway to impact of the PopART combined TB/HIV intervention on TB by measuring the yield of case finding by HIV status, change in TB notifications and reported TB incidence.
3. To use qualitative methods to assess the delivery of the PopART interventions:
    a. describing TB stigma and popular understanding of TB among community members drawing on longitudinal data (2004-18) from the selected communities
    b. describing the experience of diagnosed TB patients and their households across intervention and control sites, and household intervention and standard of care health facility services
4. To use mathematical modelling to assess:
    a. The effect of the different PopART intervention components (universal ART and screening for TB) on the impact on TB
    b. The projected impact of this intervention on TB in other settings and what factors influence this
5. Use economic analysis methods combined with mathematical modelling to measure the cost-effectiveness of the PopART intervention in terms of its impact on TB
6. To evaluate newer methods for measuring the impact of public health interventions on TB
    a. To evaluate the role of QuantiFERON Gold Plus in assessing epidemiological impact of TB interventions on infection with TB
    b. To evaluate new biomarkers for assessing the predictors of progression from infection with *M. tuberculosis* to active disease ("incipient TB")
    c. To compare a novel method of measuring the prevalence of active TB disease, including Xpert MTB/RIF Ultra combined with computer aided digital chest x-ray (CAD4TB), with traditional methods in terms of costs and efficiency
7. To increase the capacity of African field sites to conduct large cluster randomised trials through south-south as well as north-south collaboration
8. To inform global policy:
    a. On the costs and effectiveness of the PopART intervention to control TB

b. On strategies to measure the burden of TB at population level

9. To raise awareness amongst researchers, funders, programme managers and civil society of the impact of a combined TB/HIV intervention of population level screening for TB and universal HIV testing and treatment to inform future approaches to addressing TB effectively

2.2 Study Design

The **HPTN 071 (PopART) Trial** is a three-arm matched cluster-randomised trial of combination HIV prevention including universal testing and treatment for HIV being conducted in 21 communities in Zambia and South Africa. The primary outcome is HIV incidence at community level measured in a randomly selected cohort of approximately 40,000 adults (HPTN071 population cohort). The trial is sponsored by DAIDS and is managed via the HIV Prevention Trials Network (HPTN). Trial registration ClinicalTrials.gov NCT01900977.

The TREATS project is nested within HPTN 071(PopART) trial. Originally HPTN071 had two intervention arms (arm A and B) that differed in that both had the same community intervention (see below) but in arm A, HIV positive individuals received universal ART regardless of CD4 count whereas in arm B national guidelines on CD4 count were used. During the trial the thresholds for commencement of ART have changed such that universal ART regardless of CD4 count is provided in all arms of the trial. For this reason, the TREATS study will use a combined intervention arm of arm A plus Arm B.

Randomisation of the 21 communities (clusters) for the HPTN071 trial was done using matching of triplets. Within each triplet, one community was randomly allocated to each of the three study arms, using a process of restricted randomisation to ensure balance on key covariates. Communities were matched into triplets by geography and estimated baseline HIV prevalence. The randomisation was done in a public ceremony in 2013. For practical reasons there is no blinding of which cluster is in which intervention arm, however laboratory staff analysing biological samples for the primary outcomes are blinded to the arm of the study, and investigators are blinded to outcome data beyond baseline.

2.3 Timing of Intervention and Research Components

The **PopART intervention** started in January 2014 and was completed by the end of December 2017. The intervention is continuous involving three rounds of CHiPs visits to every household and follow-up visits to ensure linkage to care, adherence to treatment and retention in care for both HIV-positive and TB patients who have been diagnosed. Data collection is ongoing for TB notification at the clinic, and self-reported TB incidence within the population cohort of the HPTN071 trial. Data are being collected to measure the cost of the intervention.

The **TREATS Project** formally started in November 2017 and will continue until October 2021 (48 months).

Data for some of the secondary endpoints are already being collected as part of the main HPTN 071 (PopART) trial. These include the process indicators relating to TB being collected by the CHiPs and at the health facility level (TB notification data) as well as information on self-reported TB incidence in the population cohort. Additional data collection including qualitative assessments of

the TB component of the intervention will start as soon as possible (pending ethical and government clearance), as these need to happen while the intervention is still in place or has just been completed.

In the first part of 2018 we aim to recruit the “**Infection cohort”.** Three hundred adolescents and young adults (15-24 years of age) will be recruited in each community, from arm A and C (total 4200) over a period of 4-6 months. This cohort will be followed up for a total period of 24 months each with phone contact every 6 months and 2 annual visits. The last follow up visit will be completed in 2020.

The “**TB Prevalence survey”** will start later in 2018 with the aim of completing the survey within all 21 communities in the combined intervention arm (A + B) and control arm (C), by the end of 2019.

Data analyses and mathematical modelling will continue throughout the time of the project.

.

Figure 2- Timing of Deployment of Intervention and Research Components of HPTN071 and TREATS

	2014	2015	2016	2017	2018	2019	2020	2021
HPTN071/PopART intervention								
HPTN071/PopART data collection and analysis								
**TREATS Study**								
Incidence of TB infection cohorts								
Prevalence survey								
Implementation science (some data collection part of HPTN071)								
Analysis								
Modelling								
Policy interaction/dissemination								

3.0 STUDY INTERVENTION

3.1 Intervention

The HPTN071(PopART) intervention (arm A) consists of a package of combination TB/HIV prevention activities, including active case finding (ACF) for TB and universal test and treat (UTT) for HIV. The intervention is being delivered over a period of four years by community health workers called community HIV care providers (CHiPs) who are responsible for a zone of their community. They go door-to door within their zone ensuring that every member of the population has access to HIV and TB prevention messages, annual TB symptom screening, HIV testing and sexually transmitted infection screening, condoms, referral to voluntary male medical circumcision and, for HIV positive individuals, referral to care and ART regardless of CD4 count. During each annual visit, each member of the community is screened for symptoms of TB and sputum samples taken from any who are symptomatic for TB diagnosis, and tested using Xpert MTB/RIF (Cepheid, Sunnyvale, CA) in individuals who are HIV-positive and smear microscopy for those who are HIV-negative. All symptomatic individuals are followed up to ensure that results are available and appropriate referrals are made for further investigation if required. Diagnosed cases of TB are referred to care through routine government health care facilities (HCF) and are treated with standard 6-month TB regimens for drug-susceptible TB and referred for treatment according to national guidelines if they have drug-resistant disease. All TB treatment is provided by government HCF. All HIV positive individuals who present to the clinic for HIV care are screened for TB using a symptom screen, with symptomatic individuals being tested using Xpert MTB/RIF according to standard national policy, and are offered isoniazid preventive therapy (IPT) if they are non-symptomatic. The CHiPs collect data on the uptake of the intervention (CHiP data).

The HPTN071(PopART) intervention in arm B was the same as in arm A, consisting in a package of combination of TB/HIV prevention activities, including active case finding (ACF) for TB and UTT for HIV delivered in the community. Differently to arm A, in arm B national guidelines on CD4 count were used for ART initiation. During the trial the thresholds for commencement of ART changed from 500 CD count to 350 CD count and then, in 2016, to universal ART regardless of CD4 count.

3.2 Standard of Care

The standard of care arm C communities have access to HIV testing services (usually at the health facility), HIV care and ART provision according to national guidelines, which since mid-2016 includes ART initiation regardless of CD4 count. TB case finding is "passive" i.e. relies on individuals to present with symptoms to the health facility. TB diagnostics are as per national guidelines including Xpert TB/RIF for those who are HIV positive. All diagnosed cases of TB are treated as in the intervention arm. TB screening and prevention at HIV care clinics are as is in the intervention arm.

Table 1- Summary of Intervention Components

Study Arms	Activity
	Study Start
Arm A and B	• Enumeration of all houses in each community<br>• Division of houses into “zones” and assignment of a CHiP team to each zone<br>• CHiP Team will:<br>o Offer HIV testing with counselling to all household members (all individuals 16+ years old in Zambia and 12+ years old in South Africa and minors with the consent of their guardians) and will record HIV status with name in mobile device<br>o Provide linkage-to-care at local health centre for HIV-infected persons<br>o Screen all individuals for TB using a standard symptom checklist and collect sputum samples for testing at govt health facility or laboratory<br>o Refer/link men who are uncircumcised to circumcision, if interested, focusing on men who are HIV-uninfected<br>o Identify pregnant women and encourage them to attend for follow-up at an ANC; encourage HIV-infected pregnant women to initiate ART (Arm A) or PMTCT per national guidelines (Arm B) as part of their care<br>o Provide on-going psycho-social support for ART adherence to those on ART<br>o Screen for STI using symptom checklist and refer for treatment<br>o Provide prevention resources including condoms
	On-going Throughout the Study
	• CHiP team will:<br>o Promote community-based HIV prevention services in their zone<br>o Follow up with all persons in their zone who are identified as HIV-infected (by CHiP team or at other venues) to encourage and facilitate them to access HIV care<br>o Return to houses where residents were not available for testing during original or subsequent visits, to complete testing of all willing residents<br>o Follow up with all persons in their zone who were symptomatic for TB to ensure that testing has been completed and that treatment has been initiated if required<br>o Encourage return to care in case of defaulters from TB or ART treatment<br>o Encourage pregnant women to attend for follow-up at an ANC; encourage HIV-infected pregnant women to initiate

	ART (Arm A) or PMTCT per national guidelines (Arm B) as part of their care<br>o Provide on-going psycho-social support for ART adherence to those on ART<br>o Encourage STI treatment and provide prevention resources including condoms
	Follow Up Testing (Rounds 2 & 3)
	• CHiP teams will cycle back through their zone over a period of 4 years to repeat universal testing and screening for TB in each household for those not previously diagnosed as HIV-infected
	Procedures and Tests at the Health Centres
	• Community members who are identified as HIV-infected will receive clinical support and laboratory tests at local health centres, consistent with national guidelines for HIV treatment and care, with immediate eligibility for ART initiation (Arm A) or following national guidelines (Arm B)<br>• Community Members who are identified as having TB will be initiated on TB treatment according to national guidelines. Individuals who remain symptomatic for TB but have initial negative laboratory test results will be referred for further investigation following national guidelines
	By Study Start and Throughout the Study Period
	Endeavour to ensure that the following services are available within the community or health facility:<br>o Voluntary HIV counselling and testing<br>o Male circumcision<br>o PMTCT<br>o HIV treatment and care<br>o STI treatment and prevention resources including condom distribution<br>o TB diagnosis and treatment<br>o Provision of TB prophylaxis according to national guidelines (asymptomatic HIV + , children under five contacts)
Arm C	By Study Start and Throughout the Study Period
	Endeavour to ensure that the following services are available within the community or health facility:<br>o Voluntary HIV counselling and testing<br>o Male circumcision<br>o PMTCT<br>o HIV treatment and care<br>o STI treatment and prevention resources including condom distribution<br>o TB diagnosis and treatment

	○ Provision of TB prophylaxis according to national guidelines (asymptomatic HIV + children under 5 contacts)

4.0 STUDY POPULATION

4.1 Description/Selection of the 21 Study Communities

TREATS will work in the communities randomized to arms A, B and C of the HPTN 071 (PopART) trial. The communities selected for randomization in HPTN071 (PopART) trial were largely the communities that were selected for the ZAMSTAR[2] trial in Zambia with some overlap in South Africa with some nearby communities substituted where a community was not available for the HPTN071 trial. Selection criteria for communities included having a health facility that offered TB and HIV services, a high HIV prevalence, a TB notification rate of at least 400/100,000 per year and a total population of about 20,000 or more to minimize the effects of contamination (due to contact with other communities or residents of other communities). The communities were selected in conjunction with national and local health authorities. All communities were willing to be included in a randomized trial. Extensive work has been done in these communities where we have long standing relationships, longitudinal qualitative data and exceptional epidemiological data. We have well established community advisory boards and have invested in community representatives to ensure that they understand the fundamentals of research and they have all been very supportive during the trial.

Additional considerations that informed selection of these sites included:

- Geographically distinct
- No other major HIV prevention studies planned or ongoing
- Community willingness to be involved

For the TREATS infection cohort we will work in 8 communities in Zambia and 6 in the Western Cape province of South Africa, whereas for the prevalence surveys we will work in all 21 communities (see figure 2). The sites were members of triplets matched on geography and HIV prevalence. In Zambia the sites are communities in Kitwe, Ndola, Kabwe,Lusaka, Choma and Livingstone towns or cities. In South Africa two triplets of sites are in the Cape Town Metropolitan area and one triplet of sites are in the Cape Winelands district.

Table 2- Twenty one study clusters in Zambia and South Africa and relevant baseline data collected from a randomly selected cohort of 18-44-year old's (HPTN071 PC0 2014)

Triplet	Community	HIV prevalence (%)[1]	Told have TB in previous 12 months (%)[1]	Knows HIV-positive status, among HIV-positive individuals (%)[2]	On ART, among individuals who know their HIV-positive status (%)[3]	N[4]
1	101	15.3	0.67	70.8	88.2	984
1	102	15.2	0.97	62.2	69.6	1024
1	103	18.9	0.71	63.1	65.3	1776
2	104	16.7	1.94	41.7	60.2	1692
2	105	15.8	1.44	38.3	91.3	1966
2	106	20.8	0.75	39.5	57.8	2394
3	107	17.1	1.28	52.3	72.6	2100
3	108	16.8	0.82	45.1	60.0	1628
3	109	18.1	1.50	53.5	63.9	1387
4	110	23.1	2.05	52.8	65.2	1757
4	111	20.9	1.40	62.5	66.7	1555
4	112	25.1	2.61	69.9	64.7	1049
5	113	24.2	1.22	62.5	74.1	2325
5	114	25.9	2.31	32.8	55.0	2134
5	115	23.9	3.12	60.8	75.1	1840
6	116	21.4	2.18	45.7	38.8	2010
6	117	31.6	2.67	28.4	58.2	1961
6	118	17.6	1.22	53.1	70.8	2288
7	119	9.8	0.98	61.3	75.0	1667
7	120	9.4	0.46	32.0	79.5	1981
7	121	2.9	1.17	17.8	50.0	1488

1 weighted average, assuming 50% of population is male and 50% of population is female; 2 unweighted, calculated as number who self-reported HIV-positive / total who were HIV-positive based on laboratory testing of a venous blood sample; 3 unweighted, calculated as number who self-reported they were taking ART (in the previous 1 month) / total who self-reported they were HIV-positive; 4 N= total who participated in the cohort and have a test result (HIV-negative or HIV-positive) from laboratory testing of a venous blood sample. Estimates of knowledge of HIV-positive status, and ART uptake among those who self-report HIV-positive, are restricted (in intervention communities) to individuals who reported that the PopART intervention community-HIV care-providers (CHiPs) had not yet visited their household – so as to provide "pre-intervention" estimates

Figure 3- Location of 21clusters in Zambia and South Africa

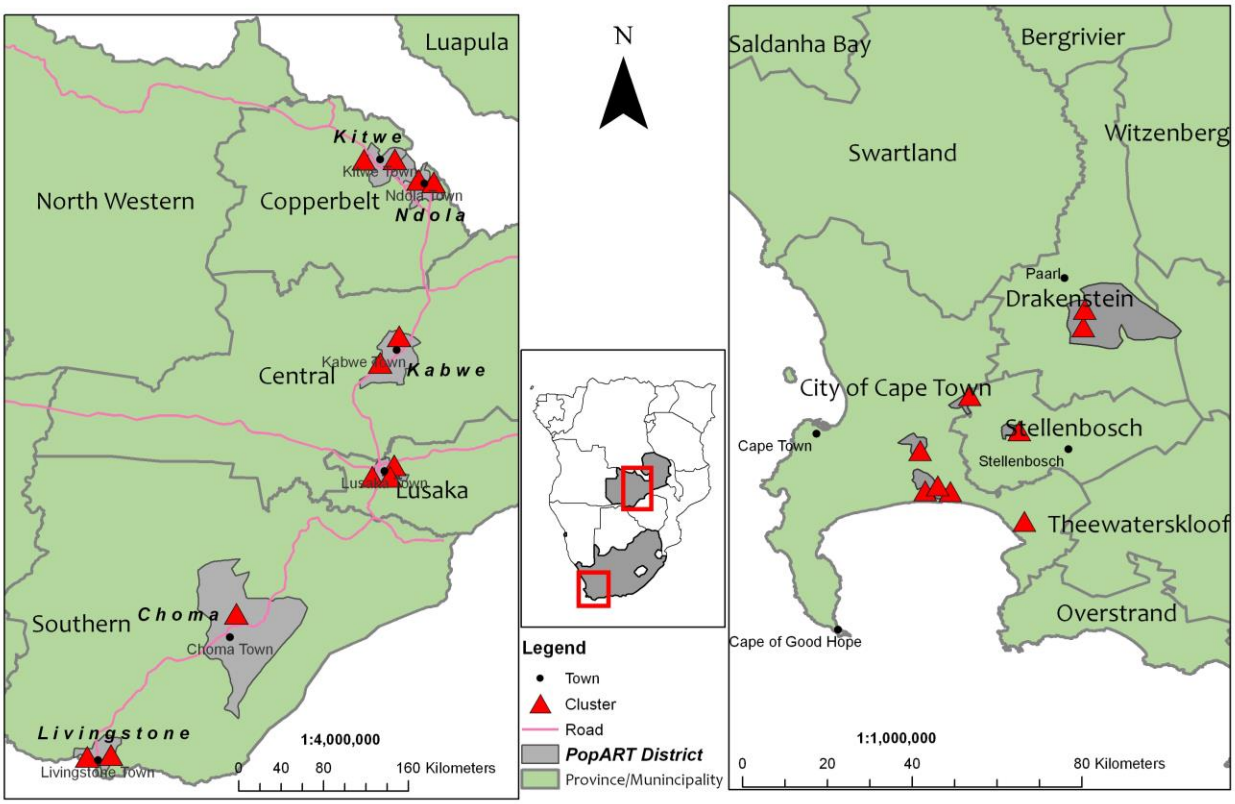

4.2 Community Engagement

This study will build on the community engagement and community capacity established during the ZAMSTAR and HPTN071/PopART trials. To work within a community successfully requires a trusting relationship which requires time to be built, and through the two trials the research team has spent 12 years engaging with these communities. In ZAMSTAR the research team worked with existing community representative structures as community advisory bodies while new ones were established in the HPTN071/PopART with input from existing advisory bodies and other community groups. Members of the advisory groups were trained in research ethics and conduct. These bodies have been invaluable during trial implementation and after to represent community views and to assist the research teams during their work in the communities. The TREATS project will build further on the bodies and experiences from the two trials , widening the constituency of these bodies where necessary.

Community engagement will be an ongoing process and will include regular consultations and interactions with community groups and other community based stakeholders, implementation of targeted mobilization activities depending on the stage or component of the study (work package) and documentation of lessons learned to inform the implementation process. Direct community engagement for TREATS will begin early, during protocol writing, when various community groups including Adolescent and Young People (AYP) Community Advisory Boards (AYP CABs) adults CABs and civil society organizations (such as the Cape Metro Health Forum, Treatment

Action Campaign and South African National AIDS Council (SANAC) and the Community Partners Platform (CPP) in Zambia will be consulted for their input. The CPP is a platform for Civil Society Organisations (CSOs) created to provide advice and oversight for the HPTN 071 trial. It comprises 10 CSOs some of which implement large TB programs while others are also strong TB/HIV advocates such as the Treatment Action and Literacy Campaign (TALC) and the Community Initiative for TB, HIV/AIDS related disease (CITAM). Consultations with CABs and CSOs will be ongoing following a schedule of meetings/ activities that will be created and reviewed at the beginning of every year.

In Zambia, TREATs will use the CABs established in the HPTN071/PopART trial (in all the eight communities) but membership will be reviewed to ensure broad representation from various community interests groups and stakeholders such as schools, Parents Teachers Associations (PTAs), churches, law enforcement, government structures at community level, TB- related community groups, health-related committees, and development-related committees. In South Africa, one CAB consisting of adults and AYPs will be created. Selection criteria will be arrived at through consultation with the stakeholders. Each study community will have a member of the study team responsible for community engagement activities. One of the main tasks of the staff will be to keep dialogue open and ongoing between researchers and community groups.

Key to the success of the TREATS community engagement strategy will be the design of key messages at the beginning of the study. The messages will relate to:

1. Work packages: Overarching messages will include the signs and symptoms of TB, study participants and selection criteria, recruitment and follow up procedures, laboratory tests and availability of results, and the benefits and risks to individuals and communities. Some messages will be specific to the different studies- for instance messages to create awareness about the Xpert and the OneStop TB Platform will be prominent during the prevalence survey.
2. Stage of the study: for the Infection cohort, sensitization/ messaging will be more intense at the beginning to support recruitment and less intense during follow up. Relatedly, the messages will/ might vary at recruitment, retention and follow up stages.
3. Implementation: Feedback from the community and other stakeholders will be used to generate messages to respond to emerging issues some of which will be as a result of rumours and misconceptions about the study. CAB members will be instrumental at identifying these issues as well as suggesting appropriate messages and channels for responding to them. The messages will be reviewed regularly to ensure that they include new developments in TB and HIV and that they remain relevant to the communities the study is being implemented in.

The messages will be communicated through various channels and mobilization mechanisms, such as flyers and leaflets to be distributed during mobilization activities. A combination of mobilization mechanisms will be utilized, such as community meetings, door to door visitations, PTA meetings, and meetings with the learners in school.

All community engagement activities will be documented. Community mobilisers (field staff) will collect data on the number of mobilization activities conducted and the number of people reached. This will enable to study to determine the depth and spread of community engagement in each study community. Case studies will also be conducted to document community members' perception of

the study at different stages of the study. Community engagement will also allow researchers to receive feedback from the community on social harms, individual and community level risks, perceptions about the study in the community, and implementation challenges. All study staff and stakeholders will receive training in Good Clinical Practice (GCP)/research ethics before commencement of research activities. Overall, strong community engagement will allow the establishment of a partnership between communities, participants and researchers to ensure the latter discharge their responsibilities ethically in the study communities. .

The TREATS study has provided the community engagement team opportunities to explore some research ideas around community motivation for participation in research studies and the ethical dilemmas of conducting research in AYP. By the end of 2021, we will have been condsucting research in these communities for 17 years. In addition, all the three studies (ZAMSTAR, PopART and TREATs) will have intensely engaged AYP. The community engagement team will explore community perspectives around research fatigue, motivation for participation and the ethical issues that arise with AYP participation in trials.

5.0 RESEARCH PROCEDURES AND ACTIVITIES

Descriptions of the Infection Cohort, Prevalence survey and Implementation Science procedures are provided below. Detailed instructions to guide and standardize all study procedures across sites will be provided in the study specific procedures manuals for each study.

5.1 Infection Cohort

The study will be conducted in the 7 arm A (intervention) communities and the 7 arm C (standard of care) communities of the HPTN071 (PopART) trial. A cohort of 300 adolescents and young adults (15-24 years of age) will be recruited in each community (total 4200).

5.1.1 Sampling/Recruitment of Infection Cohort

In 2013 we recorded the location of every household in the community. This provides a sampling frame for the selection of households. The study community area will be subdivided into blocks whereby every block consists of approximately 40 households in Zambia and approximately 55 households in South Africa, on the basis that this is expected to result in an average of ~15 individuals aged 15-24 years enrolled in each block in both countries. The blocks will be visited in a random order. An expected number of 20 blocks will be visited to reach the required sample size in each community with a minimum of 10 blocks and stratified sampling (having divided each community into “zones”) to ensure good geographical representation.

All households within a block are eligible and will be approached using a door-to-door approach. After explanation of the study, permission will be sought from the responsible adult to enumerate all household members. Household members of 15-24 years of age that have lived in the community for 2 years or more and are expecting/planning to be resident for at least 9 months per calendar year for the next 2 years, are given an invitation card and invited for participation in the study.

Recruitment

Eligible individuals responding to our invitation will be seen either in their home, at a central site for each community, this may be the local health facility or some other clinical research site or at a mutually agreed convenient place. At this site further information about the study will be provided, eligibility will be confirmed, and informed consent and assent can be provided.

Inclusion Criteria:
Usually resident in the community, (defined as spends at least 9 months of the year living there),
Residing in the community for at least 2 years previously
Intending ot stay in the community for next 2 years
Aged 15-24 years,
Able to give informed consent.
Exclusion Criteria:
Non-resident visitor,
Age <15 or ≥25 years,
Participation in TB vaccine or other TB prevention trial.
Being on TB treatment

Participants aged ≥18 years will provide written consent. Individuals <18 years will provide written assent and will need written consent from their parent or guardian before participating in the study.

5.1.2 Procedures and Activities

Baseline assessment

1. A structured questionnaire will be administered to the consenting young people by trained research assistants (RAs) which will include information on socio-demographic data, previous TB and HIV history, risk factors for TB and HIV and social networks. Locator information including contact numbers of the participant and their family will be taken to facilitate mid-year contact calls.
2. Physical measurement of height and weight will be taken.
3. A TB symptom screen will be conducted, and Xpert MTB/RIF will be done on sputum samples provided for all symptomatic participants.
4. Up to 15mls of blood will be drawn tests including QFT Plus test and HIV testing and for storage for additional testing for TB biomarkers or other respiratory pathogens.
5. Any participant who tests QFT Plus positive at baseline will be assessed by study staff for active TB disease, counselled regarding their need for isoniazid preventative therapy (currently offered to HIV positive individuals)
6. All participants will be encouraged to accept HIV testing using rapid tests as per national guidelines at baseline and throughout the follow up period. Any newly diagnosed HIV positive will be linked to ART care.
7. IPT will be offered according to national guidelines

Follow Up

All participants will be asked to come back for follow-up visits at month 12 and 24. Participants will be educated on the signs and symptoms of TB and asked to return to the study site if any

symptoms suggestive of TB are noted. Follow up contacts will be made for any participants identified as HIV-positive or diagnosed with TB to ensure linkage to care has occurred

<u>Procedures at follow up visits at 12 and 24 months</u>

1. All participants will be interviewed using a standardized questionnaire including activities and social networks as well as contacts with TB.
2. All participants will be asked additional questions about TB transmission/tracing and social contact patterns
3. All participants will have blood drawn to be retested using QFT-plus and for storage for other tests as at baseline.
4. Participants will also be screened for TB, using a standard symptom screen and asked to provide one sputum sample, so that we can test for TB disease using Xpert MTB/RIF.
.
5. All participants will be encouraged to accept HIV testing using rapid HIV testing.

.

Any participant found to have TB or HIV at any follow up visit will be referred to the clinic for further assessment and care. All HIV+ participants will have a CD4 count taken at the HCF and the participant will be asked if the research team can have access to the clinical records including the results of CD4 counts and HIV viral loads. Any participant identified as HIV+ attending the HCF will also be referred for IPT for 6 months as per national guidelines. This will be given at the ART clinic. During the study visit any other medical issue encountered by the research team will be referred for appropriate HCF services, e.g. VMMC for HIV uninfected men, contraception services, STI and pregnancy care as required.

Contact phone calls will be made between visits (e.g. at months 6 and 18) to check on the whereabouts of the participants and to check that they are well. We anticipate that this will reduce losses to follow-up.

Laboratory procedures

Up to 15 mls of blood will be taken at each visit for tests including Quantiferon Plus testing, HIV testing and for storage for additional tests to explore host and *Mycobacterium tuberculosis* (MTB) biomarkers that can predict progression to active TB disease, improve TB disease/infection diagnosis and monitor response to TB treatment, and to explore the role of different viral infections in the aetiology of TB.

Blood samples will be transported to a laboratory within 6 hours where they will be processed or stored for further testing:

- 4 mls of heparinized blood will be aliquoted into the 4 QFT-Plus tubes and incubated at 37°C for 24 hours, according to the manufacturer's instruction. After incubation the blood will be centrifuged, and the supernatant stored at -20°C before shipping to a centralized laboratory in each country for batch testing for IFN-γ release.
- Other heparinized blood will be centrifuged and separated into plasma and cells and stored at -80°C for transportation to a centralized laboratory in each country

In the central laboratory QFT-Plus testing testing will be done. In addition, testing for host and/or pathogen related TB biomarkers that are currently being identified and tests for respiratory/other viruses considered to possibly play a role in the progression to TB disease (such as CMV and influenza) may be evaluated contingent on obtaining additional funding for these tests.

5.1.3 Retention and Losses to Follow-up

To reduce losses to follow-up in the cohort the research team will collect locator information at enrolment and during the follow up phone calls. This will include asking permission to follow them at home with contact details for their own mobile phones plus those of their family and details about which school or workplace that they can be found at. Participants will be encouraged to attend their follow up appointments and will be reminded using phone call reminders or text messages. Participants who do not attend the appointments will be followed up at home (or school/workplace if permission is given) or using the phone numbers so that accurate information is obtained as to the reason for the loss to follow up. Participants who move out of their community will still be traced and encouraged to attend follow up.

Participants may voluntarily withdraw for the study for any reason at any time. The Investigator may also withdraw participants from the study to protect their safety and/or if they are unwilling or unable to comply with required study procedures.
Participants may also be withdrawn if the study sponsor, government or regulatory authorities or site Institutional Review Board (IRB) / Ethic Committee (EC) terminates the study prior to its planned end date.

5.2 Prevalence Survey

Data collection will be implemented in the two countries in all 21 communities in arms A, B and C commencing in 2019. In communities in arms A and B approximately2000 people will be enrolled and 4000 people in arm C .

5.2.1 Sampling/Recruitment of Prevalence Survey Participants

The 21 communities consist of census zones each with an estimated 300-500 households and an anticipated ~1300 adults (15 years and above) per zone. Census zones will be further subdivided into “blocks” that each include ~40-60 households, having 100-150 adults (15 years and above). The blocks will be grouped geographically to enable stratified sampling, which will ensure there is good geographical coverage of the whole community. The blocks will be randomly sampled, with stratified sampling, and then all households within a sampling block will be eligible for inclusion in the study until the targeted sample size of either 2000 for arms A and B or 4000 participants for arm C is reached for the different communities

Recruitment

Centrally in each census zone a mobile field site (MFS) will be set up where the OneStopTB Platform (a truck, containing a digital X-ray and Xpert instrument) will be stationed and tents will be erected to conduct the different survey procedures. Community mobilization activities will be conducted to sensitize community members and then each selected zone will be enumerated block by block. The census listing will be done by systematically moving house-to-house to cover all dwellings in the selected blocks. In each household all those eligible for the TB prevalence survey

(resident, and age ≥15 years) will be listed. Those eligible will be invited to the MFS. At this site further information about the study will be provided and eligibility will be confirmed.

Inclusion Criteria:
Usually resident in the community,
Aged ≥15 years,
Able to give informed consent.

Exclusion Criteria:
Non-resident visitor,
Participation in TB vaccine or other TB prevention trial.
Participation in TREATS Infection Cohort

Participants aged ≥18 years will provide written consent. Individuals <18 years will provide written assent and will need written consent from their parent or guardian before participating in the study.

5.2.2 Procedures and Activities

All enrolled participants, following consent/assent will be asked a set of questions to determine socio-demographic and other key characteristics of the survey population for analysis. Individual factors may include smoking, alcohol, education, marital status, history of working in the mines, being a health care worker. We will also include questions around TB stigma as well as questions about HIV testing, HIV status, ART use and exposure to the PopART intervention. Socio-economic status will be determined at household level using a list of assets and housing condition, although some assets like mobile phone will be assessed at individual level. Using an invitation card with barcode all enrolled participants will be invited for TB screening at the MFS.

Upon arrival at the MFS participants will be logged in at the reception desk using the barcode where their identity and eligibility will be checked after which TB screening will be conducted. TB screening will consist of symptom screening and chest X-ray for all enrolled participants. Detailed questions around health seeking behaviour will be asked to all those reporting symptoms. Digital chest X-ray (CXR) images will be read automatically using CAD4TB a software product that takes a single frontal chest radiograph as input, in the form of a DICOM image, and produces several outputs. The output includes a quality assessment of the input image, a heat map indicating possible areas of abnormalities, and an output score between 0 and 100 related to the likelihood that the X-ray is radiologically abnormal. The cut-off value will be decided based on retrospective analysis of the images from the Zambia national TB prevalence survey in 2014. This value will be evaluated after the pilot study and adjusted if needed.

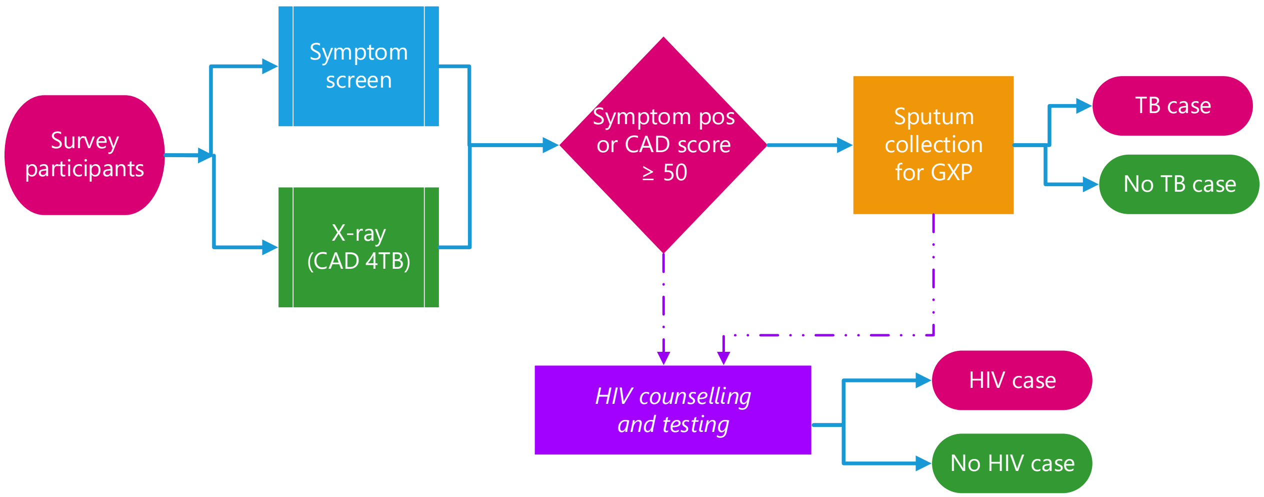

Figure 4 Screening and diagnostic algorithm for prevalence survey participants

All individuals who either have a positive symptom screen and/or an abnormal CXR (based on their CAD4TB score) are eligible for sputum examination. Participants refusing to undergo CXR or symptom screen will automatically be sputum eligible. All sputum eligible will be asked to provide sputum samples for TB diagnosis using Xpert MTB/RIF and/or sputum culture. Instruction on how to provide a good sputum sample will be provided as well as a barcode labelled container. Xpert testing will be conducted in the mobile truck by a trained technicians and results should be available within 2 hours. All those diagnosed with TB after confirmatory testing, will be referred to the nearest health facility for treatment initiation/ linking to care, following the country's national TB treatment guidelines. Confirmatory testing is needed in a TB prevalence survey setting to avoid false positive results given the lower level of TB prevalence then the routine clinic setting. In case of error results, the test will be repeated. Sputum culture and other molecular tests on sputum will also be used as a quality assurance method. Results of sputum culture are only available after 8 weeks and so for any participant who has a sputum culture done the results will be returned to them when they are available.

In addition, selected participants who have produced sputum samples, representing those likely to have TB and some with no evidence of TB will be asked for permission to draw up to 10ml of venous blood for evaluation of alternative tests, such as tests of inflammation like C-reactive protein, or new biomarkers which may be able to diagnose TB disease or predictors of progression from infection to active disease.

Blood samples will be transported to a laboratory within 6 hours where they will be processed or stored for further testing. Heparinized blood will be centrifuged and separated into plasma and cells and stored at -80°C for transportation to a centralized laboratory in each country

All participants will be offered HIV counselling testing using rapid tests as per standard protocol at the mobile field site. During pre-counselling participants will be asked if they know their HIV status and if so if they are willing to disclose (including when they last tested for HIV), they will also be asked whether they were tested in their community as part of the PopART (CHiP) intervention. Those with known HIV positive status will not be retested. Any newly diagnosed HIV positive will be linked to ART care.

Participants living with HIV and any newly diagnosed HIV positive will be asked permission for finger-stick blood sampling for viral load testing and HIV sequencing for phylogenetic and HIV drug resistance analysis. Other viruses such as hepatitis C virus will be tested for as well as HIV.

The research team will ensure the results of the CXR and Xpert MTB/RIF are given back to participants. This can be in real time or the following day. These results will be given back to the participant while attending a second visit, through a household visit by the research team or by text message. All individuals found or suspected to have TB will be referred to the local health facility for further assessment and treatment. Any culture results will be returned after approximately 8 weeks when they are available. At this point participants will be followed up to find out whether they were started on treatment and how they have responded to treatment. Further samples may be taken at this point if they are clinically needed.

To ensure other findings in the lungs besides TB are properly addressed, all images with a high CAD4TB score (to be confirmed after the pilot) will be read by an experienced clinician and assessed for referral. Referrals will be made to the nearest appropriate health care facility depending on the findings. Any participant not attending for clinical follow up will be checked by the research team within 2-4 weeks from the test.

The prevalence survey will be costed using a bottom up ingredients based approach, with data on time for participants for each stage of the procedure being recorded from the EDC. This will allow for relative costs and savings from alternative algorithms for prevalence surveys to be measured.

5.3 Implementation Science Studies

Objectives

1. To measure the effect of the PopART intervention on:
    a. Notified bacteriologically confirmed pulmonary TB incidence in the parent HPTN071 trial Population Cohort participants aged 18-44 years over the last 24 months of follow-up (2017-2018) through linkage of cohort data to routine TB case notification data
    b. Bacteriologically confirmed pulmonary TB case notification rates among adults (≥18 years) residing in the study communities, during the period 2017-2018, using routine TB case notification data
    c. The clinical characteristics and treatment outcomes of bacteriologically confirmed pulmonary TB cases in Population Cohort participants aged 18-44 years over 36 months through linkage of cohort data to routine TB case notification data
    d. The clinical characteristics and treatment outcomes of bacteriologically confirmed pulmonary TB cases among adults (≥18 years) residing in the study communities over 60 months (2014-2018), using routine TB case notification data
2. To understand the pathway to impact of the PopART combined TB/HIV intervention on TB by measuring the yield of case finding by HIV status, change in TB notifications and reported TB incidence.
3. To use qualitative methods to assess the delivery of the PopART interventions:
    a. describing TB stigma and popular understanding of TB among community members drawing on longitudinal data (2004-18) from the selected communities

b. describing the experience of diagnosed TB patients and their households across intervention and control sites, and household intervention and standard of care health facility services

5.3.1 Secondary data Analysis

Objective 1

Design: Secondary data analysis will use two data sources:

- Routine TB case notification data (held in TB registers and containing information on all patients starting TB treatment, including microbiology results, HIV-status and treatment outcomes), for all adults residing in the 21 HPTN 071 communities from 2014-2018. While this dataset has limited information on potential confounders, it is large and less vulnerable to losses to follow-up.
- Population cohort data linked to TB case notification data. Detailed individual-level data are collected annually on the cohort, over approximately 36 months of follow-up for each participant (between 2014-2018). At each visit, cohort members are asked if they have started TB treatment in the preceding year and, if so, unique TB case identifier numbers from patient cards are obtained. Population Cohort data will be linked to TB case notification data allowing all TB diagnoses in the cohort to be verified and microbiology results and TB treatment outcomes to be determined. Given the detailed individual-level data collected for all cohort participants, an adjusted analysis can be undertaken with this dataset.

Data collection and management: TB case notification data between 01/2011-12/2018, with treatment outcomes to 06/2019, will be collected. This allows historical trends and data during and immediately following the PopART intervention to be investigated. Population cohort data will be linked to TB case notification data, using either the unique TB number or an algorithm which includes variables such as name, age/date of birth, gender and year/date of TB diagnosis. Anonymised datasets (Population cohort data linked to TB case notification data and overall TB case notification data) will be generated and used to address the study objectives.

Primary outcome: Notified bacteriologically confirmed (smear, Xpert and/or culture positive) pulmonary TB incidence

Secondary outcome: All (bacteriologically confirmed and clinically diagnosed, pulmonary and extrapulmonary) notified TB incidence

Objective 2

As part of HPTN071, data on the intervention uptake in the intervention sites only are captured electronically by the CHiPs and summarised annually. These intervention process measures will be used to document how uptake of the intervention, especially TB screening and diagnosis, varies across communities and to explore the association between these measures and TB case notification rates at community-level. In addition, the intervention process measures will be analysed by age, gender and other socio-demographic characteristics to explore inequities in intervention uptake/distribution by different population groups.

5.3.2 Qualitative Studies

Qualitative studies will be conducted in both Zambia and South Africa (Objective 3)

The following activities were conducted during the third year of the PopART intervention:

- Structured direct observation of TB activities conducted by CHiPs (n~49 [7 per community]; arm A).
- Drop-in visits to households visited by CHiPs (n~28 [4 per community]; arm A), with a checklist administered to capture recall of TB activities.
- Structured observation of TB services and space at government health facilities, to understand the space used for TB services, patient flow and the influence of HPTN071 on the TB services at facilities (n=14; arm A and C).

The following activity will be conducted in 2018, as close as possible to the end of the PopART intervention period. This falls under a HPTN071 (PopART) social science component and ethical and government clearance for this component has already been obtained. The interviews will be carried out in February – March 2018.:

- Semi-structured interviews with individuals aged 18 and over and their households who are part of an existing qualitative cohort to explore the relationship between TB and HIV, popular understanding of TB, options for managing TB, TB stigma and the implications of missing TB cases. A cohort representing different HIV decisions and outcomes (to test/not test, HIV-negative/HIV-positive, PLHIV on treatment/PLHIV not on treatment) and different gender and age groups was established by the HPTN071 social science team from 2016-2018. Approximately 8 individuals in one arm A and C community per country (n=32) and their households will be revisited and asked TB-specific questions.

The following activities will be conducted in the first six months of 2018. The information and informed consent forms for these activities are in the appendices.

- Semi-structured interviews with stakeholders providing TB services, to determine TB services available (arm A and C), including a historical perspective of the history of TB services in each particular site. We will liaise closely with the community engagement team for this activity.
- Focus Group Discussions with 10-12 CHiPs in each Arm A site (n=84 approximately), to document restrospectively their experiences of delivering TB services within the wider PopART intervention and with particular communities. In Zambia, CHiPs would be recruited amongst the group retained for a post-intervention period. In South Africa, previous CHiPs would be approached.
- In-depth interviews with key TB staff to describe their experience with TB service delivery and the role of other stakeholders (n=14; arm A and C).
- In-depth interviews with TB patients and their households diagnosed through CHiPs in intervention sites (n=49 – 7 per Arm A Site across both countries). TB patients and their households will be identified with the assistance of the intervention data and CHiPs. In-depth interviews will be conducted using participatory techniques including body mapping

and network charts (for mobility and TB pathways) to determine the role of CHiPs, the patient's experience of falling ill with TB and the enablers and barriers to diagnosis and treatment from a household and patient perspective.

- In-depth interviews with TB patients and their households diagnosed through passive case finding (standard of care at health facility level) in health facilities in intervention sites (n=77 – 7 per Arm A Site across both countries and 7 per Arm C site in Zambia only). The research tool will be similar to the comparative interview of those diagnosed through CHiPs but explore, rather, the role of the health facility and other services. TB patients will be identified through the TB services at the health facility and in each site we would aim to recruit a range of age and gender groups (aged 18 and over). We would ask TB patients if we can accompany them home and interview them and their household at home.

6.0 SAFETY MONITORING AND SOCIAL HARM REPORTING

6.1 Safety Monitoring

All the communities participating in the intervention have already been exposed to the intervention for 4 years. Since this is a trial of a health systems intervention there are no anticipated adverse events in a traditional sense but social harms due to HIV testing and disclosure were anticipated. The trial has reported several of these which have been managed according to the specific situation that pertained.

In the measurement of additional TB outcomes there are also unlikely to be major risks to participant safety. Social harm due to participation in the studies will be minimized by ensuring good community level engagement and understanding of the procedures involved and by providing participants adequate time to make decisions regarding their participation, including allowing them to seek permission from spouses and heads of households. In addition, a standard way of identifying and documenting social harms exist in the HPTN 071 (PopART) study. Study staff will be trained in how to identify and determine if an incident is indeed a social harm; whether it is related or not related to the study; the type and amount of information to be collected as evidence of occurrence of a related social harm; how to classify the harms and depending on the classification when to report to the PIs.

The procedures involved in the TREATS project include blood sampling and CXR. Neither of these, properly conducted, is expected to cause any risk to participants and all staff will be fully trained on correct procedure and monitored on these procedures. Because of the TREATS studies some individuals will be diagnosed as having TB disease and commenced on treatment. While this is beneficial to them, as earlier treatment has demonstrated better outcomes, there is always a risk from adverse effects of anti-tuberculosis therapy including liver impairment. All treatment will be provided and monitored according to national standard guidelines and additional training will be provided to the clinical teams in the study sites to ensure compliance with these guidelines.

Chest radiography may identify other conditions apart from tuberculosis. Abnormal CXR over a certain threshold CAD score will be reviewed by a radiologist and appropriate referrals will be made according to a standardized SOP on unexpected findings. Participants will be informed of the

possible abnormalities and encouraged to attend referrals as arranged. Assistance will be provided where possible to facilitate attendance at the appropriate health facility.

Some of the communities where the study is based have risks due to violence especially gang violence. We work carefully with the community advisory boards, community leadership and bodies such as the police to ensure that any activities conducted in these communities is safe. Representation on community advisory boards will be sought from relevant organisations. For instance, in Zambia the Victim Support Unit (VSU), a department of the police that deals with gender based violence and child abuse is represented on most advisory boards. A review of the composition of all advisory boards will be done to ensure this representation. Both field research sites have standard operating procedures concerning field safety in place and these will be reviewed regularly. All staff are in communication using mobile phones. Staff always work in pairs and have transport support nearby. Should there be any situation considered by the leadership to be unsafe for staff working, they will be recalled from their duties within the communities.

7.0 STATISTICAL CONSIDERATIONS AND DATA ANALYSIS

7.1 Sample Size

The trial has been powered to detect intervention impact on the primary endpoint, and on key secondary endpoints, as detailed below. All sample size calculations have been carried out using methods for matched cluster-randomized trials.

7.1.1 Mathematical Modelling to inform Sample Size Calculations

To determine the likely effect of the HPTN071 (PopART) interventions on TB epidemiology, a deterministic, compartmental mathematical model was developed in R. Run over one-month time-steps, it captured the intervention and control arms and allowed comparisons across arms over time.

The control arm modelled routine ART initiation among people living with HIV (PLWH). HIV compartments captured 4 CD4 strata (<200, 200-350, 351-500 and >500) and 3 ART strata (naïve, recent [<1 year], established [1+ years]), with risks of TB disease incidence modelled for each combination of CD4 and ART strata. 40% of PLWH were assumed to be on ART at baseline from all CD4 strata (HPTN071 data). 10% of PLWH were assumed to annually start ART (recent ART). The mean duration of incident TB disease was assumed to be 1.2 years among those HIV-negative and ⅓ of a year among PLWH. During this time cases were infectious undiagnosed prevalent TB disease cases, contributing to the annual risk of TB infection (ARI).

In the intervention arm, the model captured the effects of 3 rounds of universal testing and treatment for HIV (UTT) and screening for TB over 4 years on TB epidemiology. UTT was modelled to identify a proportion of PLWH from all treatment naïve CD4 strata (HIV diagnosis/linkage). This proportion was moved into the recent ART strata. Therefore, UTT affected TB disease incidence among PLWH and hence, TB disease prevalence. TB screening was modelled as a series rounds over 4 years identifying a proportion of undiagnosed prevalent TB disease cases (screening efficiency). This proportion was assumed to apply equally to all undiagnosed prevalent disease cases, irrespective of HIV-status. Therefore, TB screening had a direct effect on TB disease prevalence. The 3 rounds were modelled at months 8, 24 and 40 of the HPTN071 (PopART) study.

Baseline data for the communities (e.g. TB disease prevalence and ARI) were available from the ZAMSTAR trial[2], conducted in the same study areas between 2006-2010. This allowed TB disease incidence at baseline to be determined (incidence=prevalence/duration). From this equilibrium at baseline, the model was run, capturing the dynamic monthly and cumulative changes in TB disease prevalence and the incidence of TB infection in the study arms and relative to one another.

Table 3: The modelled effect of the HPTN071 (PopART) interventions on TB disease prevalence and the incidence of TB infection

Screening efficiency	HIV diagnosis/linkage	Effect on disease prevalence (risk ratio)*	Effect on incidence of infection (risk ratio)§
		Combined Intervention Arm A+B/C	Arm A/C
10	40	0.68	0.71
20	40	0.59	0.60
30	40	0.51	0.51
40	40	0.44	0.44
10	50	0.66	0.69
20	50	0.57	0.59
30	50	0.49	0.50
40	50	0.43	0.43
10	60	0.64	0.68
20	60	0.55	0.58
30	60	0.48	0.49
40	60	0.42	0.42

*Changes in disease prevalence modelled in the combines intervention (A + B) and control (C) arms over 73 months from a baseline prevalence of 832/100,000 population (a minimum TB disease prevalence estimate for the communities from the ZAMSTAR trial), and prevalence at month 63 in the combined intervention (A + B) and control (C) arms are compared; the TB active case-finding rounds of the of the HPTN071 (PopART) intervention were modelled at 8, 24 and 40 months. §Comparison between the cumulative incidence of TB infection modelled in the intervention (A) and control (C) arms over the 24 months between month 48.5 and month 72.5, used to capture the duration of the incidence of TB infection cohort. screening efficiency=proportion of undiagnosed TB cases identified through the TB screening strategy; HIV diagnosis/linkage=proportion of PLWH not on ART who are identified and started on ART; ACF=active case finding; PLWH= people living with HIV; ART=antiretroviral therapy

Using this mathematical modelling to estimate the likely effect of the intervention on the co-primary outcomes of the study, which are the prevalence of active TB among individuals aged ≥15 years and the incidence of new infection with *M. tuberculosis* among young adults aged 15-24 years, there is estimated to be a reduction of between 30%-60% in both adult TB prevalence and the incidence of new infection with *M. tuberculosis* among young adults. The range in intervention effectiveness varies according to the efficiency of the interventions.

7.1.2 Sample size for Primary Endpoints

For our sample size calculations and estimates of study power, we reviewed the projections of intervention effectiveness that were derived from the mathematical modelling and on that basis we assumed that the reduction in adult TB prevalence and the incidence of new infection with *M.*

tuberculosis would plausibly be in the range 40-50%; study power will be higher if the effect of the intervention is >50%. We calculated study power using standard formulae for a triplet-matched cluster-randomised trial.

To estimate study power to detect a 40-50% reduction in adult TB prevalence in the combined intervention arm relative to the control arm, we assumed that:

(a) Adult TB prevalence will be in the range 0.8% to 1% in the control arm. This estimate is based on the TB prevalence survey that was conducted in 2010 to measure the primary endpoint of the Zamstar trial [2]. In this prevalence survey, TB prevalence was on average 0.55% in Zambian trial communities, and 2.34% in South African trial communities, based on taking a single sputum sample from all study participants and culturing each sputum sample for *M. tuberculosis*. A weighted average, with a weight of 4/7 for Zambia and 3/7 for South Africa to reflect the balance of communities between the two countries in the TREATS study, gives an average of 1.32%. Accounting for the fact that Xpert has lower sensitivity than culture, but assuming the sensitivity is at least 67% compared with culture, we estimate that TB prevalence will be measured as ~0.9% in the control arm communities in the TREATS study.

(b) The coefficient of between-community variation k will be in the range 0.2 to 0.25 [2]. In the Zamstar trial, within each of Zambia and South Africa the trial communities were grouped into two strata based on estimates of the prevalence of infection with *M. tuberculosis* among schoolchildren at the start of the trial. Thus, the trial had 4 strata (2 countries, and 2 strata within each country). In the TB prevalence survey conducted in 2010 to measure the primary endpoint of the Zamstar trial, the coefficient of between-community variation in TB prevalence – among communities in the same strata - was estimated to be k=0.29. Taking account of key covariates, however, the data were consistent with a lower value of k in the range 0.20-0. 25. In the TREATS study, we have assumed similar values of k in the range 0.20-0.25 because the communities have been matched into triplets on geographical area and adult HIV prevalence, and the primary analysis will adjust for known risk factors for TB thus reducing between-community variation.

With a sample size of 4000 adults in each Arm C community and 2000 adults in each Arm A and B community (56,000 in total across 21 communities), study power is high, in the range 82%-97%, if k=0.2. With k=0.25, the study is moderately powered at 72-76% if intervention effectiveness is 40%, well powered at 83-86% if intervention effectiveness is 45%, and very well powered at 90-93% if intervention effectiveness is 50%.

To estimate study power to detect a 45-50% reduction in the incidence of new infection with *M. tuberculosis* in the intervention arm relative to the control arm, we assumed that:

(a) The incidence of new infection with *M. tuberculosis* will be in the range 5-6 per 100 person-years in the control arm. This estimate is based on 3 information sources: (i) estimates of the prevalence of infection with *M. tuberculosis* among schoolchildren aged 6-11 years in Zamstar trial communities in 2005 [17](ii) direct measurement of the incidence of new infection with *M. tuberculosis* among schoolchildren who were followed up in 2009, 4 years after a baseline survey in 2005 [2], and (iii) estimates of the ratio of the incidence of new infection with *M. tuberculosis*, comparing young adults to children, based on data collected in 2010-11 on social contact patterns and mathematical modelling [18]. From (i), the incidence rate of new infection is in the range 0.8-1.3 per 100 person-years in Zambian study communities and in the range 2.5-4.2 per 100 person-years in South African study communities, among schoolchildren aged 6-11 years old. From (ii), the incidence of new infection with *M. tuberculosis* was 1.2 per 100 person-years in Zambia study

communities, and 4.5 per 100 person-years in South African study communities, using a very stringent definition of tuberculin-skin-test (TST) conversion that was measured among children with a TST response of 0mm at baseline and a TST response of ≥15mm at follow-up. Based on (i) and (iii), we estimate that the incidence of new infection with *M. tuberculosis* among 6-11-year olds is ~1.2 per 100 person-years in Zambian study communities and ~4.0 per 100 person-years in South African study communities. A weighted average, with a weight of 4/7 for Zambia and 3/7 for South Africa to reflect the balance of communities between the two countries in the TREATS study, gives an average of 2.4 per 100 person-years. Based on (iii), we estimate that the incidence of new infection with *M. tuberculosis* among young adults is approximately 2-3 times higher than among schoolchildren.

(b) the percentage of 15-24-year olds who will not be infected with *M. tuberculosis* at baseline in the TREATS cohort study in the control arm, will be ~75% in Zambia and ~45% in South African study communities, if the median age of cohort study participants is 20 years and that the incidence rate of new infection with *M. tuberculosis* up to the age of 20 has averaged ~1.2 per 100 person-years and ~4 per 100 person-years in Zambia and South Africa study communities respectively. With these assumptions, ~60%-65% of young adults who are enrolled into the cohort will not be infected with *M. tuberculosis* at baseline, and will be followed up to measure the incidence of new infection with *M. tuberculosis*.

(c) the coefficient of between-community variation k will be in the range 0.15 to 0.2. We do not have information from the Zamstar trial about the between-community variation in the incidence of new infection with *M. tuberculosis* among young adults. However, if we assume that (i) in Zambia the range in the true incidence of new infection with *M. tuberculosis* across study communities is 1.5-3.5 per 100 person-years, with a mean of 2.5 per 100 person-years and (ii) in South Africa, the range in the true incidence of new infection with *M. tuberculosis* across study communities is 6-14 per 100 person-years, with a mean of 10 per 100 person-years, then k would be 0.2 in both countries. Since the trial is pair-matched within each country, the overall value of k would also be k=0.2.

With a sample size of 300 young adults per community (4,200 in total across 14 communities), and if ~60% are not already infected with *M. tuberculosis* at the time of enrolment, ~180 individuals will be followed up to measure the incidence of new infection with *M. tuberculosis*. If ~90% and ~80% are followed up at 12 and 24 months respectively, there will be ~300 person-years of follow-up per community over 2 years. With k=0.15 study power is 79%-92%, and with k=0.2 study power is 72%-86%, to show intervention effectiveness of 45-50% if the incidence rate of new infection with *M. tuberculosis* is 5-6 per 100 person-years. With intervention effectiveness of 50%, study power is 82%-92% for k in the range 0.15-0.20 and the incidence rate of new infection with *M. tuberculosis* in the range 5-6 per 100 person-years.

7.2 Data management

A detailed data management plan will be written within 3 months of the start of the study to cover all data management issues. This plan will be updated on an annual basis as necessary.

For the **Infection Cohort**, questionnaire data and venous blood samples will be collected, at 3 time points. For individuals with TB symptoms, sputum samples will be taken. Questionnaire, blood sample information on HIV infection, blood sample information on infection with *M. tuberculosis*, and sputum sample information on TB disease, will be held in separate database tables that all

uniquely identify an individual with a barcode identifier and can be linked using this barcode identifier. Personal identifying information will be held separately, on a paper locator form that includes the unique barcode identifier. Data will be collected on ~4,200 individuals at baseline.

For **TB Prevalence surveys**, questionnaire data will be collected, and a digital chest X-ray and fingerprick blood sample for rapid HIV testing will be taken/collected for individuals who consent to this. For all participants who screen positive based on TB symptoms and chest X-ray findings, sputum samples will be requested. A blood sample may also be requested from participants who are eligible to provide sputum samples and an additional capillary blood sample from those HIV-infected. Questionnaire, blood sample information , HIV status, X-ray results, and sputum sample information on TB disease, will be held in separate database tables that all uniquely identify an individual with a barcode identifier and can be linked using this barcode identifier. Personal identifying information will be held separately on a database that includes the information recorded on invitation cards that are given to all who are eligible to participate in the survey, including the unique barcode identifier. Data will be collected on ~56,000 participants.

For **Implementation Science**, there will be a separate database for TB notification data for each of Zambia and South Africa that includes an extract of key information from routine TB registers. From these 2 separate databases, a standardised database that combines the data from the two countries will be created, without names or any other personal identifying information. The database on self-reported TB incidence, among participants in the HPTN071 (PopART) population cohort, will be created as an extract from this research cohort study database.

All data, except the locator forms with personal identifying information, will be captured using an electronic handheld device (tablet computer) with password protection on 2 levels (unlocking the device, and logging on to the data entry program). The tablet computers are set up so that they cannot be used for any purpose other than data capture. The data entry forms will ensure 4 levels of data integrity/validation: (1) field integrity by validating the entered value against a range of possible values, (2) record integrity by validating an entry against values entered elsewhere in the record, (3) table integrity by avoiding duplicate values and (4) referential integrity ensuring consistency between different entities (e.g. households and their members, or across multiple visits for the same person). Additionally, the quality of data collection and data entry will be maximised through comprehensive and work-package-specific training of study staff, and those responsible for electronic data capture will be required to demonstrate competence before starting work on the study.

All personal identifying information (including name, address, house number, telephone number, GPS-coordinates, and health care identifiers) will be encrypted. Data will be synchronized daily to a central server at ZAMBART using the cellular network and a virtual-private-network (VPN). The electronic data capture systems to be used will build on ones that have previously been used successfully for large-scale data collection in Zambia and/or South Africa, for example for the national TB prevalence survey in Zambia, the Zamstar community-randomised trial, and the HPTN071 (PopART) study.

All data will be stored in the relational database of Microsoft SQL-Server, on a secure server at Zambart, except for the database holding the South Africa electronic routine TB register data which will be stored at HST. Access control will be ensured by using password protection to the local area network, and using the standard access control facilities of Microsoft SQL-Server to tailor the specific access rights for different datasets to authorized staff. Databases will be backed up daily

and backups kept for ≥2 years. An audit trail of all entries and updates will be kept on the server including the time stamp and log-in name of the user responsible for the entry/change.

Data checking and cleaning will be ongoing throughout the study, and implemented in both Microsoft SQL-Server – Transact SQL and Stata, with rapid (at least weekly) flagging of missing data and discrepancies for quick resolution. During the prevalence survey, daily checking and flagging of missing data and discrepancies will be done by mobile data managers who work alongside the field teams, to target rapid resolution of data issues. At the end of each month, a copy of all databases will be securely transferred to LSHTM, for the LSHTM team to work together with the Zambart and HST teams to check and monitor the data while the study is ongoing.

Standard operating procedures for data checking and data management, including for ensuring data security and confidentiality, will be developed and finalised prior to the start of data collection. Data cleaning will be completed within 6 months of the last data being collected, and once it is complete "locked" databases will be created.

Qualitative data management: Data collected through interviews, Focus Group Discussions and semi-structured interviews which will both be audio recorded (if consent is given) and notes will be taken by social scientists and social science research assistants. Data summaries of the activities will be written up close to data collection. Recordings and notes will be kept in a locked place whilst in the field. Data tracking sheets both in the field and at head office will track the form and location of data. The recordings will be transcribed verbatim and recordings will be destroyed once transcriptions have been checked for quality. During the transcription process, the translation of the content from local languages into English will also done following a transcription guide. All field notes taken during interviews and observation will be written up in Microsoft word within a structured activity report form with prompts.

All the qualitative data that will be collected in this study will be stored on a password protected computer and backed up on an external hard drive at Head Offices in which the documents and recordings will be password protected. Hard copies of the data (e.g. researcher field note books) will be stored in a lockable drawer. After finalizing the transcription and writing up of observations, all data transcripts and observation notes will be loaded into Atlas.ti version 7, a qualitative data analysis software that will be used to help with further data management and analysis.

7.3 Statistical Analysis

The statistical analysis will use standard methods for a pair-matched cluster-randomised trial with <15 communities in each study arm in the infection cohort and triplet-matched cluster –randomised trial communities in the prevalence survey. We outline below the analytical approach for two co-primary outcomes, one that is measured as a prevalence and one that is measured as a rate. Other outcomes will also be measured as a prevalence or a rate.

For the TB prevalence outcome, we will present the geometric mean of the community-level TB prevalence values in the 14 communities randomised to receive the PopART intervention and the corresponding geometric mean from the 7 communities randomised to receive standard of care. For the community-level prevalence values, we will use multiple imputation of missing values for individuals who are eligible to provide a sputum sample (because they screen positive on symptoms or X-ray) but who either do not provide a sputum samples or there are missing data on laboratory test results, to limit bias in the prevalence estimates from each community. The ratio of the two

geometric means gives an unadjusted prevalence ratio for the comparison of the 2 study arms. For the primary analysis, we will use a two-stage approach to adjust for individual-level covariates that are known risk factors for prevalent TB disease, including age and gender, and at the second stage we will conduct an analysis of variance to obtain an adjusted prevalence ratio and corresponding 95% confidence interval and p-value.

For the Infection cohort outcome, we will present the geometric mean of the community-level values of the rate of new infection in the 7 communities randomised to receive the full PopART intervention and the geometric mean of the rate of new infection in the 7 communities randomised to receive standard of care. For the primary analysis, we will use a two-stage approach, to adjust for age and gender, and at the second stage we will conduct an analysis of variance to obtain an adjusted rate ratio and corresponding 95% confidence interval and p-value.

For case notification data analysis, all data will be analysed by year and overall. The overall analysis of notified bacteriologically confirmed pulmonary TB incidence will be restricted to the last 24 months of follow-up for both the Population cohort data linked to TB case notification data and the overall TB case notification data, to exclude the initial rise in TB cases anticipated with TB screening. Clinical characteristics and treatment outcomes of pulmonary TB patients will be analysed over the entire duration of follow-up (36 months for Population Cohort and 60 months [01/2014-12/2018] for the overall TB case notification data).

A **statistical analysis plan** (SAP) will be completed by study month 12 for the prevalence survey and 36 months for the incidence cohort. The SAP will set out the methods of analysis in detail, including exactly which individual covariates will be adjusted for in the primary analysis. The statistical analysis will be jointly led and implemented by the LSHTM and Zambart teams, and in the case of the TB prevalence survey also together with the KNCV team.

Qualitative analysis plans will be developed around key data, questions, findings and outputs. Debriefing after fieldwork will be a first step in analysis. For finer analysis, coding framework/s and definitions will be developed to code the data in Atlas.ti qualitative data management programme.

7.4 Matematical modelling

The model software will be developed through the project as an open source R package, with the core deterministic compartmental transmission model written in Fortran 90.

There are three main components to this model development work:

1. Adaptation of an existing underlying demographic and HIV/ART model that has been developed for the HPTN071 trial in discussion with the HPTN071 (PopART) HIV-modelling team
2. Adapting an existing TB transmission model in this framework to represent the TB interventions in TREATS, and contact the TB data collected by PopART and TREATS
3. Adapting this product to generate output data and perform analyses required for health economic evaluation of the TB intervention

Component 1. HIV transmission will be modelled phenomenologically, with HIV incidence and changes in ART coverage derived from the estimates from the PopART HIV-modelling to ensure a consistent account of HIV epidemiology. To facilitate this, the underlying

demographic/HIV/ART structure (currently 81 age categories, 2 sexes, HIV-uninfected + 7 CD4 categories for PLHIV, and 4 ART durations) and the HIV/ART natural history parameters will be modified to ensure the demographic/HIV/ART model substrate aligns with the approach taken in the PopART HIV modelling. The focus will be in reproducing in the TB model substrate the patterns of HIV infection by age and sex, and the estimated distributions of CD4 cell count and time-since-ART initiation, which strongly influence TB risk, to capture the influence of the PopART HIV interventions on TB incidence.

Component 2. The TB transmission model will follow established model structures (including latent TB infection, fast and slow routes of progression to disease, re-infection and progression, increased risks of relapse after treatment), and sit on top of the HIV/demographic layer, accounting for increased risks of TB progression due to HIV-related immune-compromisation (i.e. linking TB risk to CD4 count and time on ART). HIV/ART status will also affect the natural history of TB disease. The model will also be modified to include the TB active case-finding aspects of PopART, and to include a likelihood that contacts the routine data and actively collected data (disease prevalence, infection incidence).

Component 3. To be used for projections and health economic evaluation, additional outputs will be generated to, first, estimate incremental health benefits of future TB infections averted in terms of disability-adjusted life years (DALYs), second, estimate healthcare cost savings due to TB infections averted, and third, estimate incremental costs of active case-finding for TB in the context of UTT projected into the future. In addition, the model will need to be modified to project forward beyond the end of the intervention, and be made flexible enough to consider other settings.

Calibration. The approach to calibration will follow a fully Bayesian paradigm, with all parameters treated as random variables with uncertainty described by prior distributions. Sampling from the posterior will be undertaken using Markov chain Monte Carlo (MCMC) techniques to generate samples that represent the uncertainty in all parameters, conditional on the observed data. These samples (together with corresponding model outputs) will therefore serve as a basis for appropriate probabilistic sensitivity analysis (PSA) of uncertainty surrounding certain outputs, including incremental benefits and costs of health economic evaluations. Non-parametric techniques will be applied to the PSA datasets to obtain estimates of multi-way parameter uncertainty and value-of-information. Calibration will initially be undertaken before TREATS outcome results using baseline data and routine data. This will serve as preparation for a final calibration including TREATS outcome data, and allow an assessment of the improvement in precision generated by including the outcome data.

Data analysis & preparation. There will be continuous effort to collate and analyse process and outcome data from PopART and TREATS for use in modelling.

Literature review. Literature review work will be undertaken to generate data for modelling, and to inform the economic evaluation. This will focus on two areas: recent epidemiological literature informing on the natural history of TB for individuals on ART; and then later the epidemiological characteristics of other settings identified as relevant to generalizing findings beyond the PopART communities.

7.5 Cost-effectiveness Analysis (CEA).

The basic premise of the CEA of TREATS is to provide important information to decision-makers on the following two aspects:

- Is a combination of UTT for HIV along with active case finding for TB worth implementing as compared to standard care, in South Africa and Zambia, but also in other high endemic countries?
- Are there economies of scope from jointly providing UTT and active case finding for TB compared to only UTT for HIV?

The epidemiological and economic analyses will be closely integrated. The CEA will take a "Health systems" perspective and include all costs and benefits associated with provision of HIV and TB care. Outcomes will be valued in terms of TB infections and DALYs averted, and will be projected into the future.

7.5.1 CEA data.

Calculation of the cost and benefits will use primary data collected from health service facilities, data and results from other costing studies that have already been implemented and published literature sources. A summary table of data and sources is presented below:

Table 4: Summary table of data sources

Category	Specific measures	Data sources
**Benefits of ACF for TB and UTT**	Direct health benefit data (DALYs)	Secondary data sources
**Health Facility Level Cost of TB and HIV Services**	All costs associated with provision of TB and ART services in health facilities	Health Facility Costing Survey / Secondary Data
**Costs of implementing PopART and TREATS**	Intervention implementation costs	TREATS and PopART study team records

Economic costs will be collected via a bottom-up micro-costing approach from intervention expenditure data of Zambart and implementing partners, and compared against facility level costing of HIV and TB prevention under Standard of Care. Costs components considered are salary, test kits (Xpert), other equipment, and travel. Results of a time-and-motion study conducted under HPTN071(PopART) will estimate the time spent by CHiPs on the TB component.

The TREATS CEA will collect health facility costs of providing TB services from some of the health facilities in Zambia and South Africa, to ensure good representation. The objective of the facility level costing survey is to estimate per-patient-per-year unit costs of typical TB related tests and treatments. In both countries, two facilities from the control communities of PopART and 1 facility from the intervention communities will be chosen according to predefined criteria. For

example, important selection criteria are that the facility is broadly representative of the availability and characteristics of care provision, it serves the population in the study communities and that the facility has accounting records of a satisfactory standard. Health facility costs of HIV related care have already been collected under PopART from all trial facilities.

From the surveyed facilities, costs for prevention under standard of care will be collected (such as household contact tracing and IPT provision), and components considered are salary, test kits, and IPT. The costs of caring for TB patients at all stages of the disease will also be collected from healthcare facilities, to calculate the healthcare costs saved of TB infections averted. Shared expenditures on equipment, building, and general overheads will be apportioned to TB prevention and care according to activity data. Unit costs estimates of the cost per person tested and cost per TB case identified will be estimated. An economist in country will support the modelling team to collect and analyse the facility costs of TB prevention and treatment, and the costs of the intervention. All efforts will be made to contact the facility prior to arrival on-site, to schedule an appropriate visit day and time. When the economist arrives at the facility, s/he will first communicate with the facility administrator/manager. If the administrator/manager is unavailable, s/he will seek the next individual in charge. Once contact is made with the facility administrator/manager and permission has been given, the economist will proceed with the data collection exercise. Costs of care for drug-resistant TB, outside the surveyed health facilities, such as at tertiary health care units will be gathered from secondary data sources.

Specifically, the facility costing exercise will endeavour to capture the following data categories in an excel costing tool developed for PopART:

- Direct costs
  - Personnel (for clinical care staff, laboratory and pharmacy, including pensions)
  - Drugs
  - Laboratory supplies
  - One-time training costs
  - Data sources (including but not limited to): Stock lists /flows at different time periods, procurement records, receipts/income statements at the facility level or central level, tenders or contracts that show standardized pricing in country, facility laboratory records, laboratory budget, facility reports or M&E data that can provide number of tests performed by patient type. Facility payroll and staffing records, donor payroll and staffing records, MOH payroll and staffing records
- Indirect costs
  - Personnel (clerical and non-clinical staff times, managerial and supervisory roles, etc)
  - Utilities, travel, etc.
  - Data sources (including but not limited to): Accounting records for all expenses relating to utilities and running costs (maintained at facility, donor, MOH or centrally) Facility payroll and staffing records, donor payroll and staffing records, MOH payroll and staffing records, secondary data from other studies.
- Fixed and Investment costs
  - Buildings
  - Equipment and infrastructure
  - Training

  - Data sources: Accounting records and receipts for all purchased material, interviews with staff and facility management
- Overheads
  - Facility specific overheads relevant to TB care.
- Patient numbers
  - Patient population including (but not limited to) number of patients tested, diagnosed, on treatment, completed or defaulted treatment. This provides the denominator of the cost per person per year estimate.

8.0 HUMAN SUBJECTS CONSIDERATIONS

8.1 Collaborative Partnerships

At all stages of the development of this research protocol community representatives have been involved in the design and have engaged with the research team to finalize the intervention and study questions. The research teams will continue to actively engage with the study communities at various levels (for example with government structures, health care facilities at management and worker level, existing community forums and stakeholder groups) utilizing a range of communication and interaction strategies, as appropriate. In both countries, study committees will be formed with representation from trial staff and in-country stakeholders (from governmental to community representation) to provide guidance and feedback to the study team. Community advisory boards have already been established representing all communities as part of the HPTN071 (PopART) study and these will be maintained for the duration of the TREATS project.

8.2 Social Value

As described in Section 1, TB is now the leading cause of death due to an infectious disease worldwide. Interventions to reduce the burden of TB at community level have not been proven and so these studies will provide much needed evidence about the effectiveness of interventions to reduce the chances of an individual becoming infected with or developing TB.

8.3 Scientific Validity

This study will provide evidence to either support or refute the mathematical model discussed earlier, which has indicated that if a high proportion of the population can be screened for TB and treated as well as increasing access to HIV treatment and care then TB could be drastically reduced and potentially eliminated as a public health problem in the longer term. The study has been powered to determine the impact of the interventions on the primary and secondary endpoints. The multi-community cluster-randomized study design chosen for this study has, we believe, the best chance of providing an answer to the research question as the intervention is applied at community level and would have impact at this level.

The study will be conducted according to the most rigorous standards of research and is therefore expected to give definitive answers about the process of implementing the intervention as well as the impact of such an intervention at community level. The study results will be shared throughout

the study with national and international policy-makers to ensure that the findings are understood and that lessons from the study are implemented.

8.4 Compliance with ethical standards

The principal investigator will have overall responsibility for all ethical conduct of the studies and delegation of the activities involved in ensuring that all studies are conducted according to the highest ethical principles will be made to individual study PIs and site research leads according to a delegation log, held centrally and in each field site.
All research will be fully compliant with national and international standards including Good Clinical Practice standard, the declaration of Helsinki and the Oviedo bioethics convention as well as with national and EU law. All research protocols and forms will be reviewed by the appropriate ethics committees before any participants are enrolled. Any changes to the study protocol will be appropriately recorded as a protocol amendment and will be submitted for ethical review prior to implementation. All staff employed on the study will be trained in Good Clinical Practice with biannual refresher courses.

8.5 Fair Subject Selection

In the incidence of infection cohort, a total of 4,200 healthy participants aged 15-24 years will be recruited from the 14 sites. In the prevalence surveys a total of 56,000 community members aged 15 years and above will be recruited from the 21 sites. Some of the participants will be vulnerable because they are under the legal age of consent for research (18 years and above in Zambia and South Africa), because they may be illiterate and because they may be poor and so vulnerable to undue pressure provided by incentives to join studies. In the case of vulnerable young people, under the age of consent, parents or guardians will be asked to give informed consent in accordance with requirement, and the Participant will give assent to take part in the study. In situations where potential participants are illiterate a thumb print will be accepted as consent, in accordance with the expected method of obtaining consent. To avoid coercion, we will ensure that any compensation or gifts given to the participants are commensurate with the costs incurred and will ensure that all are fully approved by local ethics authorities.

Reasons for inclusion of Minors

For the TB incidence cohort, inclusion of young people under the age of 18 years has a critical scientific value as outlined in the work package justification of this cohort. Briefly, young people are the most likely to have negative TB immune responses which can subsequently become detectable in the case of new or recent TB infection and hence inform the population rates of onward TB transmissions within the communities. Adults living within these communities who over their lifetime may have been exposed to TB (even if they remain asymptomatic) could have detectable immune responses to TB and hence it would be much more difficult to determine recent infection. In addition, there is some biological evidence that this age group are at increased risk of TB infection and disease. For this study dependent on the age of the participant guardian or parental consent with young person's assent will be sought.

Volunteers for social or human sciences research

For qualitative studies, a total of 126 purposively selected TB patients and their households will be interviewed; 98 in Arm A sites across both countries and another 28 in Arm C sites in Zambia only

only to understand their comparative experience of TB. In addition, a total of 14 health care workers who deliver TB care will be interviewed (one health care worker in each of the 14 health facilities). TB stakeholders are anticipated to number between 10 to 20 in each site since an earlier PopART social science HIV stakeholder survey in 2013 had a range of nine to 33 stakeholders in each site (stakeholder participants could therefore number between 140 to 280 approximately). Approximately 84 CHiP respondents will participate in focus group discussions. Some participants may be vulnerable due to them being illiterate or poor. In addition, TB patients may be vulnerable due to feeling that they have limited choices over participation or that non-participation may affect their care. Health care workers can also be vulnerable as they may feel that their participation and what they say may affect their employment. CHiP respondents may interpret their participation as linked to future employment. TB stakeholder respondents may also link their participation to relationships with government and employment.

Selection of study participants

For the Infection cohort and the TB prevalence survey zones of the community will be randomly selected and participants who live in these zones, who meet the inclusion criteria for the study will be invited to participate. For the qualitative research activities yet to be carried out and linked to this protocol, recruitment will vary according to the participant type and activity, and according to willingness to participate. The stakeholders will be recruited according to their actual presence as TB stakeholders in the selected communities during the period of the PopART study. Health workers specific to TB at health facilities will be identified according to their key role in TB service delivery, for example, the TB corner nurse in Zambia would be approached. The CHiPs will be recruited in Zambia from the group of CHiPs retained for the post-intervention period, and in South Africa, previous CHiPs will be invited to attend. The TB patients diagnosed through CHiPs will be identified through the intervention data and through introductions from CHiPs. TB patients diagnosed through standard care at health facilities would be approached at the health facility through liaising with TB health facility staff.

8.6 Risk-Benefit Assessment

This community-based, cluster-randomized study can potentially incur risk of harm at both a community and an individual level. Likewise, study-related benefits may accrue at both an individual and a community level.

Any community-based research project may present risks to a community. Communities may feel disempowered by having a research agenda imposed on them or they may be placed at risk of stigmatization by the publication or dissemination of research results. Large community research projects may disrupt intra-community social structures and networks that are not always easily understood by an external research team.

In some of the communities' violence is commonplace and so all measures possible will be put in place to safeguard participants and researchers in these situations. Personal information will be safeguarded as mentioned above and staff will be trained how to deal with any situations on the ground. The communities will be informed about the research activities prior to beginning the study through the engagement of Community Advisory Boards and other community sensitization activities. This will help to clear any misconceptions with the presence of the study teams in the communities. Particular care will be exercised during times such as national elections or other situations when there is heightened risk of violence. Safety SOPs will be in place and police will

be informed about any community events such as the TB prevalence surveys. Staff will work in pairs and will be in contact with mobile phones.

Trial insurance will be in place via the lead member of the consortium, LSHTM.

8.6.1 Procedures that may pose a risk to individuals and mitigation of risk

Blood sampling

The main invasive procedure is that of venous blood sampling. We will collect up to 15ml of blood from individuals in the infection cohort on 3 occasions, once per year, and up to 10 ml from selected participants in TB prevalence survey. All blood sampling will be done by trained phlebotomists and, so we anticipate few adverse effects. The staff will be trained in how to handle possible vasovagal episodes.

We will collect up to 1ml of capillary blood with a fingerprick, using specially designed lancets and collection techniques, in those HIV-infected participants in the prevalence survey. Procedures will be performed by trained study staff, so few adverse effects are anticipated. The staff will be trained in how to handle possible vasovagal episodes.

Chest X ray

All participants in the prevalence survey will be offered a digital chest X-ray. The new digital x-ray machine used in this study uses a low dose of radiation and has a concentrated beam mimimizing radiation risk. All participants will be offered protective lead shielding before having their Chest X-ray taken. Any participants that refuses to undergo X-ray will be requested to submit a sputum sample instead

Sputum Collection

All sputum collection will be done in well ventilated spaces, preferably outside, away from others to avoid possible risks of infection. Individuals will be trained on how to produce the best sputum sample.

HIV testing

All HIV testing in this study will involve fingerprick rapid testing following nationally approved test algorithms. HIV testing has been one of the main interventions in these study communities and following WHO and national guidelines we are trying to normalise HIV testing. All testing will be provided by a trained counsellor who will be available to provide whatever support an individual needs following testing. The results of the point of care HIV tests will be reported back to the consenting study participant in a confidential manner in accordance with national guidance on counselling and testing for HIV. A discussion of the results of the HIV Rapid test will be given by the researcher making clear that there is a small possibility that the test result is not 100% accurate as this might depend on the recency of exposure and repeat HIV testing is recommended for all every 3 months.

HIV and phylogenetic analysis

Finger prick samples will be collected from people living with HIV in communities included in the TB prevalence survey. The purpose of doing HIV sequencing and phylogenetic analysis is to examine patterns of HIV transmission and to identify factors associated with HIV transmission in these communities.Other viruses such as hepatitis C virus will be tested for as well as HIV.

Strict measures will be in place to safeguard confidentiality of data. A key feature of this analysis is that it will not require contact tracing, invading the privacy of study participants, or otherwise compromising the anonymity of study participants. The key goal of this testing is to determine patterns of viral transmission at a population level, not to determine individual level viral transmissions.

The sample will be labeled with a unique participant ID and all laboratory specimens, reports, study data collection, process, and administrative forms related to this analysis will be identified by this coded number to maintain participant confidentiality.

Personal identifiers will only be collected for (1) informed consent and (2) operational and logistical purposes. Personal identifiers will appear on paper or electronically on consent forms and other listings. These listings will NOT include any (sensitive) information (including laboratory data). A unique study number will be used to link personal identifiers to this analysis information. Personal identifiers and link logs which may be on paper will be stored in a locked cabinet. Electronically kept personal identifiers and link logs will be stored in separate datasets with password protection only accessible for designated staff (for computers and servers).

The names of the individual communities will not be used in public presentations or publications of the study results. In the study database, however, community-level data will be discernable. These community links/identifiers will be maintained to preserve the possibility that community-specific results might be used by public health authorities to plan and target valuable prevention and treatment efforts.

Diagnosis of illness

During the study we will diagnose some individuals with HIV and some with TB. We will ensure rapid referral to the health facilities that exist in all study sites for further treatment. These clinics have been part of the main HPTN 071 (PopART) trial and so have had HIV and TB services enhanced. TB treatment and antiretroviral therapy are available for all who are diagnosed, irrespective of CD4 count, in accordance with current National ART treatment guidelines and are free of charge. The Xpert not only diagnoses TB but also diagnoses rifampicin resistance, identifying likely multi-drug resistant TB (MDR TB). All individuals diagnosed with TB through the study will be started on standard TB therapy in accordance with national TB treatment guidelines. TB and ART will be provided by the HCF in accordance with national guidelines and monitoring. All study participants who are found to have rifampicin resistant TB on Xpert testing will be referred for further sputum testing, at Zambart in Zambia or via the NHLS in South Africa according to the national guidelines in place. MDR treatment is available in both countries and individuals will be referred to the nearest MDR treatment centre as per national guideluines. The study will facilitate this referral with transport support.

During CXR screening it is likely we will identify individuals who have other lung pathologies. Any individual with a very abnormal CXR, determind by a high CAD score (see above) will have the CXr read by an experienced radiologist with a report transmitted to the filed team so that they can make appropriate referrals either to the local health facility for simple situations or to a secondary referral centre if a more complex situation is uncovered. A standard operating procedure for dealing with unexpected findings will be developed by the study team before the commencement of the prevalence survey laying out specific procedures for any unexpected findings. The partcipant information sheet will detail this as a possible finding.

Collection of Personal Data

Personal data will be collected in both the infection cohort and the prevalence survey. We will also be collecting sensitive data about HIV status on all participants.

All data collection and management procedures are outlined in the data management section of the protocol. In short, all individuals will be assigned a bar coded unique identifier which will be used to link their different data elements. All personal identifying information (including name, address, house number, telephone number, GPS-coordinates, and health care identifiers) will be encrypted. Data will be synchronized daily to a central server at Zambart using the cellular network and a virtual-private-network (VPN). The electronic data capture systems to be used will build on ones that have previously been used successfully for large-scale data collection in Zambia and/or South Africa, for example for the national TB prevalence survey in Zambia, the ZAMSTAR community-randomised trial, and the HPTN071 (PopART) study.

Tracking of participants

Participants in the infection cohort and qualitative cohort will be followed up over time. Participants will be asked to provide addresses and phone numbers to enable follow up but will be free to refuse to provide this information. All locator information will be stored in locked filing cabinets in secure offices and will not be stored with any other data on the participant.

International standards on data processing

All study staff will be trained in GCP and their responsibilities towards data security principles. The data collection, data management and data sharing procedures of this study will follow international standards for GCP (ICH-GCP), the European directives for processing of personal data (EU Data Protection Directive 95/46/EC and the UK 1998 Data Protection Act) and the LSHTM Information Management and Security Policy. Informed consent forms will include information on how personal data will be collected, stored and used. Data transferred from one site to another will be anonymized as far as possible and will be subject to a data transfer agreement.

8.7 Informed Consent

In a community-based, cluster-randomized trial such as this one, informed consent needs to take place at several levels ranging from **consent from the government authorities**, to so-called "community consent", and finally to individual consent.

Information sheets

All participants will be provided with full information in the form of an information sheet translated into appropriate languages (English, Bemba Tonga, Nyanja, Lozi, Afrikaans and Xhosa). All information sheets will be approved by all relevant ethics bodies prior to use and will be written in language that is commonly understood in the communities concerned. The information sheets will also have contact details of one local community advisory board member. Community advisory boards will review all information sheets prior to their finalisation to ensure that the content is appropriate.

Trained research assistants will assist the participant to read through the information sheet and ensure that all the contents are fully understood. Participants will be made aware that their participation is voluntary and that they may terminate their participation at any time and that termination of their consent will not in any way affect how they will be treated by any health care professional. In addition, all the study procedures will be explained to participants and the risks and benefits of these procedures will be clearly explained. Participants will be made aware of their

rights in case of any adverse reaction or social harm due to the study procedure and whom they may contact to receive additional information on the study, including the PI or country PI and the relevant ethics committees. Participants will be encouraged to ask any questions relating to the study and to ensure that they have received satisfactory answers to their questions. Any reports of social harm will be reported to the study PI and leadership and to the ethics committee if deemed appropriate. As obtaining informed consent is a process and not a one-time event, a refresher discussion of the study will be provided to the participants at each contact with the study staff.

For illiterate participants an impartial witness who is literate will be asked to be present with the participant through the entire informed consent process including the reading of the information sheet. The witness will be asked to follow the information sheet as the research assistant reads it out loud to the participant to witness that what has been explained is the full information.

Written consent

All participants will be asked to provide written consent before any study procedures are undertaken. In the case of minors (<18 years) they will be asked to provide written assent to be part of the study and then written consent will be obtained from their parent or guardian.

In the case of illiterate participants, they will be asked to mark the consent form with a thumbprint and the witness who has been present throughout the procedure will be asked to sign that they witnessed the procedure and are happy that the participant consented freely with an understanding of all relevant information.

Incentives

Participants in the infection cohort are healthy volunteers and as such will be reimbursed for their time and any costs such as travel that they incur. The reimbursement will be modest so that it does not unduly influence participation and will therefore not be considered coercive. The proposed reimbursements will be reviewed by the relevant ethics committees and only approved amounts will be given out to participants.

Participants in the prevalence surveys will be provided with a token of appreciation for taking part in the survey such as a T shirt, cap or a calendar,except where national standards mandate a financial reimbursement. Any reimbursement or token of appreciation will be given out at the end of the recruitment process and not before. Community advisory board members will be asked for their opinion on the appropriate types of the tokens so as not to cause undue distress in the community.

Participants in the qualitative research will be provided with refreshments equivalent to a snack (for example, a soft drink and biscuits) during the qualitative research activities. As far as possible particpants will be interviewed in a place convenient to them and close to or at their home or workplace that doesn't involve transport costs. However, if travelling to a venue on public transport is required to attend a research activity (for example ex CHiPs travelling to attend a FGD), transport costs will be reimbursed at an approved amount reflective of actual costs. Community advisory boards and local ethics committees will be consulted (similar to the infection cohort and prevalence surveys) about appropriate compensation for participation in qualitative research activities.

8.8 Independent Ethical Review

Ethical clearance for the trial will be sought from Institutional Review Boards (IRBs) in the United Kingdom (UK), Zambia and South Africa. Adverse events will be reported on a regular basis

according to the individual requirements of these IRBs. In instances where there is disagreement or discordant IRB requirements the condition providing the highest level of human subject protection will be implemented. Approval must be obtained from the local, and national (where relevant) IRBs before the study can be initiated.

8.9 Respect for Participants and Communities During and After the Study

8.9.1 Confidentiality

Strict measures will be in place to safeguard confidentiality of data. All laboratory specimens, reports, study data collection, process, and administrative forms will be identified by coded numbers only to maintain participant confidentiality.

Personal identifiers (name, address, global positioning system coordinates) will only be collected for (1) informed consent and (2) operational and logistical purposes (i.e. to ensure tracing of participants by intervention staff and to locate cohort participants for follow-up visits). Personal identifiers will appear on paper or electronically on appointment books, consent forms, log books, follow up lists and other listings. These listings will NOT include any (sensitive) study information (including laboratory data). A unique study number will be used to link personal identifiers to study information.

Personal identifiers on paper will be stored in a locked cabinet. Electronically kept personal identifiers will be stored in separate datasets with password protection only accessible for designated staff (for computers and servers). Hand-held devices will also be password protected and personal identifiers will be stored in an encrypted format.

All electronic data will be stored in password protected database systems. Read and write authorization of data will depend on the designation of the staff member. A second layer of protection is hardware password protection on computers, servers and networks. Thirdly data transfer over wireless or mobile networks will use Virtual Private Networks or router protected dedicated internet protocol addresses.

All collected study data on central computers and servers, remote computers and hand-held devices, will be backed up daily. Backup tapes/discs will be stored separately from the primary electronic storage.

No individuals or communities will ever be identified in the reporting of the study findings. All communities will be represented by a number only, except when community level dissemination to that specific community is being done.

8.9.2 Study Monitoring

A Study Advisory Group will be formed to advise the research team and oversee the progress of the project. This group will be headed by Dr Waafa El Sadr PI of the HPTN network and ICAP director and membership will include representatives from international agencies such as WHO and UNAIDS as well as a representative of the regional networks for people affected by TB/HIV; an independent ethics advisor will also be appointed to the study advisory group to advise on ethical issues, plus representatives from the Ministry of Health/Department of Health of both Zambia and South Africa. This Group will meet at least annually.

At community level the study will be monitored by the community advisory boards who will ensure that the study complies with all requirements and respects the communities where it is being undertaken.

The parent trial, HPTN071(PopART) trial has been monitored and audited via the HPTN and DAIDS systems. The proposed TREATS project is measuring endpoints of this trial and so will need quality control and monitoring activities that may be slightly different to those of monitoring a trial.

In the field sites regulatory affairs officers will work with the study teams to ensure all standard operating procedures are in place and that the same procedures are followed in both countries and all sites. Internal monitoring will be done on a regular basis by the study leadership in both countries and by the overall PI in all sites as they are enrolling and recruiting participants into the studies. For the prevalence survey KNCV team will conduct regular monitoring of the process and the data, which since it is electronic can be reviewed in real time.

LSHTM will act as the sponsor for these studies and will conduct at least 1 audit visit during the period of field work for each of the studies.

8.9.3 Dissemination of results

All study findings will be disseminated to the communities involved as well as to local and national health leadership before any international dissemination.

9.0 LABORATORY SPECIMENS AND BIOHAZARD CONTAINMENT

9.1 Local Laboratory Specimens

Each study site and Local Laboratory will adhere to standards of Good Clinical Laboratory Practice (GCLP), the laboratory SSP manual, and all activities related to processing, labelling, testing, storage, transport and shipping if required.

9.2 Quality Control and Quality Assurance Procedures

Study staff will conduct periodic visits to each field site to assess the implementation of on-site laboratory quality control (QC) procedures, including proper processing, labelling, storage, proper maintenance of laboratory testing equipment and use of appropriate reagents. Study staff will follow up directly with site staff to resolve any QC or QA problems identified through proficiency testing and/or on-site assessments.

9.3 Specimen Storage and Possible Future Research Testing

Left over specimens will be stored locally for possible additional testing with new diagnostics currently being developed to test for biomarkers for TB infection and progression to active TB (incipient TB), or for associated viruses that may precipitate TB infection or disease. Any transfer of specimens will necessitate a materials transfer agreement to be in place.

10.0 ADMINISTRATIVE PROCEDURES

10.1 Project Management

For the TREATS Project Professor Helen Ayles will be the overall PI of the project, and will chair the project management group (PMG) representing all research consortium partners. This PMG will lead the project and will meet by means of monthly teleconference. Annual consortium meetings will be held meeting in a rotating manner in UK, Zambia, South Africa the Netherlands and France.

10.2 Quality Management

Quality management plans will be put in place in each country that will cover the quality control and quality assurance processes. These will include setting standards for protocol and GCP training and certification, internal review of consent forms and internal data quality monitoring. Within the laboratory system quality management plans will be implemented that will cover the monitoring of blood sample collection, temperature monitoring of the reagents and samples, times to processing and the results of assays. For the prevalence surveys a random sample of CXR as read by the CAD will be reviewed by the study clinicians to ensure that there are no issues with the CAD. All positive Xpert results will be checked by doing a second sample to ensure that false positives are excluded as far as possible.

10.3 Investigator's Records

The study site investigator will maintain, and store in a secure manner, complete, accurate and current study records throughout the study. The investigator will retain all study records for at least three years after submission of the final Financial Status Report. Study records include administrative documentation — including protocol registration documents and all reports and correspondence relating to the study — as well as documentation related to each participant screened for and/or enrolled in the study — including informed consent forms, locator forms, case report forms, notations of all contacts with the participant, and all other source documents.

10.4 Use of Information and Publications

Publication of the results of this study will be governed by the PMG. Any presentation, abstract, or manuscript will be submitted to the PMG for review prior to presentation or publication.

11.0 REFERENCES

1. Stop TB Partnership. The Global Plan to End TB 2016-2020: The Paradigm Shift. Geneva: Stop TB Partnership, 2015.
2. Ayles H, Muyoyeta M, Du Toit E, et al. Effect of household and community interventions on the burden of tuberculosis in southern Africa: the ZAMSTAR community-randomised trial. *Lancet* 2013; **382**(9899): 1183-94.
3. World Health Organisation. Global Tuberculosis Report 2016. Geneva: WHO, 2016.
4. World Health Organisation. The End TB Strategy: , 2014.
5. Lonnroth K, Corbett E, Golub J, et al. Systematic screening for active tuberculosis: rationale, definitions and key considerations. *The international journal of tuberculosis and lung disease : the official journal of the International Union against Tuberculosis and Lung Disease* 2013; **17**(3): 289-98.
6. World Health Organisation. Systematic Screening for active tuberculosis: priciples and recommendations. In: Tuberculosis, editor. Geneva; 2013.
7. Bond V, Floyd S, Fenty J, et al. Secondary analysis of tuberculosis stigma data from a cluster randomised trial in Zambia and South Africa (ZAMSTAR). *Int J Tuberc Lung Dis* 2017; **21**(11): 49-59.
8. Granich R, Lo YR, Suthar AB, et al. Harnessing the prevention benefits of antiretroviral therapy to address HIV and tuberculosis. *Current HIV research* 2011; **9**(6): 355-66.
9. Hayes R, Ayles H, Beyers N, et al. HPTN 071 (PopART): rationale and design of a cluster-randomised trial of the population impact of an HIV combination prevention intervention including universal testing and treatment - a study protocol for a cluster randomised trial. *Trials* 2014; **15**: 57.
10. Corbett EL, Bandason T, Duong T, et al. Comparison of two active case-finding strategies for community-based diagnosis of symptomatic smear-positive tuberculosis and control of infectious tuberculosis in Harare, Zimbabwe (DETECTB): a cluster-randomised trial. *Lancet* 2010; **376**(9748): 1244-53.
11. Suthar AB, Lawn SD, del Amo J, et al. Antiretroviral therapy for prevention of tuberculosis in adults with HIV: a systematic review and meta-analysis. *PLoS medicine* 2012; **9**(7): e1001270.
12. Dodd PJ, Garnett GP, Hallett TB. Examining the promise of HIV elimination by 'test and treat' in hyperendemic settings. *AIDS* 2010; **24**(5): 729-35.
13. Dodd PJ, Knight GM, Lawn SD, Corbett EL, White RG. Predicting the long-term impact of antiretroviral therapy scale-up on population incidence of tuberculosis. *PloS one* 2013; **8**(9): e75466.
14. Woldehanna S, Volmink J. Treatment of latent tuberculosis infection in HIV infected persons. *Cochrane Database Syst Rev* 2004; (1): CD000171.
15. Churchyard GJ, Fielding KL, Grant AD. A trial of mass isoniazid preventive therapy for tuberculosis control. *The New England journal of medicine* 2014; **370**(17): 1662-3.
16. Adams LV, Talbot EA, Odato K, Blunt H, Steingart KR. Interventions to improve delivery of isoniazid preventive therapy: an overview of systematic reviews. *BMC Infect Dis* 2014; **14**: 281.
17. Shanaube K, Sismanidis C, Ayles H, et al. Annual risk of tuberculous infection using different methods in communities with a high prevalence of TB and HIV in Zambia and South Africa. *PLoS One* 2009; **4**(11): e7749.
18. Dodd PJ, Looker C, Plumb ID, et al. Age- and Sex-Specific Social Contact Patterns and Incidence of Mycobacterium tuberculosis Infection. *Am J Epidemiol* 2016; **183**(2): 156-66.

12.0 APPENDICES

APPENDIX I
INFORMED Consent Forms
INCIDENCE OF TB infection cohort

Tuberculosis Reduction through expanded antiretroviral therapy and TB screening (TREATS)

Incidence of TB Infection Cohort-(18-24 years old)-Information and Consent Form

Participant Information Sheet
Please ask the study investigator or the study staff to explain any words or procedures that you do not clearly understand.

This form gives you information about the research study you are being asked to join. The form describes the purpose, procedures, benefits, and risks of the research study. Please read this Information and Consent Form and ask as many questions as needed. You should not sign this form if you have any questions that have not been answered to your satisfaction. If you choose to sign this form you are giving permission to be included in this research study.

This study is being funded by the European and Developing Countries Clinical Trials Partnership (EDCTP)

Your participation is voluntary
You do not have to take part in this study if do not want to. Access to health care from the health centres in your community will not be affected if you choose not to participate in the study. You are also free to withdraw from the study at any stage, without consequences to you or your family.

What is tuberculosis (TB)?
TB is an infectious airborne disease caused by bacteria (germs) which are spread through droplets from coughing or sneezing which are inhaled in into the lungs. The TB germ mainly affects the lungs but it can also affect other parts of the body. When the TB germ enters your lungs, we say you have **TB infection.** When you have TB infection, you are usually healthy and do not feel sick at all. You may have TB infection for some time which can be for some weeks up to years. Some people may have TB infection and may never feel unwell. If you start to feel unwell because of the TB germs in your lungs, we say you have **TB disease.**

Purpose of this study
The TREATS study is made up of 4 studies that will look whether the PopART intervention that has been carried out in some communities, reduced the chance of getting TB infection or developing TB disease. The PopART intervention involved HIV testing and treatment as well as screening for the symptoms that suggest you might have TB. In total fourteen (14) communities are included in this research, 8 in Zambia and 6 in South Africa.

This particular study is called the "Incidence of TB Infection Cohort". In ***[insert the name of the country]*** this study aims to recruit a total of ***[insert number of participants recruited in***

each site] young people (300 from each community) aged 15-24 years. You have been selected to be one of the people from your community who we are asking to take part in this study.

What will happen during this study?
If you agree to take part in this study, you will need to be seen either at home, at a central site for each community, this may be the local health facility or some other clinical research site or at a mutually agreed convenient place three times: today, in one year (12 months), and in 2 years (24 months). We will contact you to remind you about your visits by either calling or sending a short text message (sms).

Today and at future visits we will:

- Ask you questions about your risk of infection with TB including what you do, where you go and whether you have been in contact with someone who has had TB.
- Take physical measurement of height and weight
- Collect up to 15mL of blood (about 3 teaspoons) for tests including Quantiferon Plus test (to assess evidence of TB infection), HIV antibody testing and for storage for additional tests. Blood samples will be stored until all study-related testing is complete and will then be destroyed. The stored blood may be used to try to understand TB better and discover new tests for TB, by testing both your genes and looking for TB genes and other markers to see if lung and other infections are associated with becoming sick with TB**.**The blood samples will be securely stored both inside and outside the country. Blood collection will be performed by qualified and fully trained personnel.
- Any participant who tests QFT Plus positive at baseline will be assessed by study staff for active TB disease and counselled by qualified and trained personnel regarding their need for isoniazid preventative therapy according to national guidelines
- Ask you to provide ***[insert number of samples needed depending on site requirements]*** sputum sample (s) if you have any signs or symptoms, so we can test for TB disease.
- Offer to perform an on-the-spot HIV test at each visit. ***[Insert whether a separate informed consent needs to be signed for HIV testing]***. Counselling before and after being tested will be provided by qualified and fully trained personnel.
- Any participant found to have TB or HIV at any follow up visit will receive counselling by qualified and trained personnel and be referred to the clinic for further assessment and care.

What are the possible risks or discomforts?
You may become embarrassed, worried or anxious when learning your HIV or TB infection status. A trained staff member will help you deal with any feelings or questions you have.

It is very rare to have any problems from having a blood test, but you may feel discomfort, dizzy, or even faint when your blood is drawn. Redness, pain, swelling, bruising may occur where the needle goes into your arm, but this is rare. The amount of blood we are taking for this study is up to 3 teaspoons and your body can very quickly replace the amount.

What are the potential benefits?

During the study you will learn whether you have been infected with TB and if so linked to appropriate care to prevent TB disease. Also you will learn more about what this means and the signs and symptoms of TB disease. You can also decide if you would like to learn your HIV status and be provided with information on where to receive treatment and care services if needed. We will be able to offer you a finger prick blood test for HIV which we can tell you the results today in about 15-20 minutes. You will also be able to ask questions about your health.

In addition, knowledge gained from this study may help reduce the spread of TB at community level and in the country. The results will help design better programs to control TB and HIV and promote better health for you and your family as well as helping with acceptance of TB as a community-wide health problem.

Are there any alternatives to participation?

If you decide not to take part in this study, we will refer you to other places where you can be screened for TB disease or receive an HIV test.

How will my confidentiality and privacy be protected?

We will do everything possible to protect your confidentiality if you join this study. We do this by giving you a study number (for example, 1234782) and any information will be labelled with this number only, so people working in the health centres and laboratories will only see a number not your name; only the research staff will be able to link this number to your name. All records identifying you will be kept confidential, and to the extent permitted by applicable laws and regulations, will not be made publicly available. Your personal information (name, address, phone number) will be protected by the research staff. No personal information will be included in the study, or in any of the data that will be forwarded to the sponsor or sponsor representatives. This information will not be used in any publication of information about this study. You will be identified by a coded number in any reports of publications produced from this study (study data). Your personal information will only be used to contact you if any of the results of the tests necessitates that.

People who may review your records include ***[insert any site-specific institutions, national regulatory authorities and local research committees].*** Ethics Committees (ECs) are committees that watch over the safety and rights of research participants. All personnel accessing your records are required to respect your confidentiality at all times. By signing this document, you are authorizing such access.

To protect your privacy, you will meet with the researcher in a private area.

Data Protection

What kind of information will be collected from you?

During this study we will collect general information such as your gender, age, home address and employment status. You will also be asked to provide information about the type of house you live in, tobacco and alcohol intake & how you spend your social time. You will also be asked questions about TB and HIV. No one will be able be to recognise you in all of the data that will be collected. A barcode ID with your study number will be allocated to you and will be used instead of your name.

[Insert any other site-specific regulations that need to be included in this section, i.e. refer to national Protection of Personal Information Act].

How will data be recorded?
Some of the information that you give us will be recorded on paper for example the consent form that you will sign, and test results. Other information like the questionnaire will be recorded electronically and will be recorded on a hand-held device. The hand-held device is securely protected by a password only known by the Research Assistant. All this information will be assigned a barcode ID so that your confidentiality is maintained.

How will it be stored?
All paper copies that will have your information will be kept securely in a locked cabinet in a locked room that will only be accessed by assigned study staff. All information that is recorded hand-held devices is accessed only by the Research Assistant. All electronic data will stored on a server and will be encrypted and password protected and will only be accessible by the data manager.

All the information collected will be stored for approximately 7 years after the study has ended after which, data will be destroyed.

Who will the information be shared with?
The data that we collect, but not your name or anything else that can identify you will be shared with other researchers working on the TREATS study. These include researchers working at Zambart in Zambia, health Systems Trust in South Africa, the London School of Hygiene and Tropical Medicine, London School of Economics, Imperial College and the University of Sheffield in the UK and KNCV in the Netherlands. We will publish study results in medical journals, for meetings and on the internet for other researchers to use. Your name or personal information will not appear in any publication.

After the study is complete copies of the data, without any details that could identify you, will be made publicly available via the internet for other researchers to use. To make sure you can never be identified we will remove information such as your name, where you live, your date of birth, the name of your community and any other data that may lead to someone being able to identify you.

What happens if I am injured by participating in this study?
It is very unlikely that you could be injured because of taking part in this study. However, if you are injured while taking part in this study, you will be given immediate treatment for your injuries and referred to the health facility. You will be compensated if an injury occurs during any of the study procedures. You will not be giving up any of your legal rights by signing this Information and Consent Form.

All principal investigators and sites are covered by the LSHTM sponsorship insurance, and have specific Medical Malpractice Insurance to cover claims.

Will I receive any payment?
If you take part in this study, you will be refunded your transport costs ***[insert amount for each site]*** for each regular study visit.

What are some reasons why I may be withdrawn from this study without my consent?
You may be withdrawn from the study without your consent for the following reasons:

- The research study, or this part of the study is stopped or canceled
- The study staff feels that completing the study or this part of the study would be harmful to you or others

Persons to Contact for Problems or Questions
If you have any questions about taking part in this research study, your rights as a research participant, or if you feel that you have experienced a research-related injury, contact:

Principle investigator's Name: ***[Insert name PI]***

Research Site Address (es): ***[Insert PI's address]***

Daytime telephone number (s): ***[Insert PI's telephone number]***

If you have any other questions or concerns about your rights as a research participant or want to discuss a problem, get information or offer input, you may contact:

Independent Review Board/Ethics Committee: ***[Insert IRB and/or Ethics Committee]***

Address of Independent Review Board: ***[Insert IRB and/or Ethics Committee's address]***

Daytime Telephone Number: ***[Insert IRB and/or Ethics Committee's telephone number]***

[Insert any other required authorities to be contacted for each site]

Tuberculosis Reduction through expanded antiretroviral therapy and TB screening (TREATS)

Incidence of Infection Cohort (18-24 years old)-Consent Form

STATEMENT OF CONSENT

I have been given sufficient time to consider whether to take part in this study.

My taking part in this research study is voluntary. I understand that I may decide not to take part or can withdraw at any time from the study without penalty or loss of benefits or treatment to which I am entitled.

I understand the research study may be stopped at any time without my consent.

I have been told how long I may be in the research study and have been informed of the procedures and tests that may be performed during the research study, as well as of the possible risks and benefits. I have had an opportunity to ask questions about this research study and my questions have been answered to my satisfaction.

I understand that the information I have given will be published in reports and papers, but that confidentiality will be maintained and it will not be possible to identify me from any publications.

I have been informed that my data will be shared with the partners and organisations that are working with ***[insert name of the site***] on this study.

I understand that confidentiality will be maintained and it will not be possible to identify me.

I understand that I do not give up my legal rights by signing this form.

I understand that I will receive a signed and dated copy of this Participant Information and Consent Form.

If you have either read or have heard the information in this Participant Information and Consent Form, if all your questions have been answered, and if you agree to take part in the study, please print and sign your name and write the date on the line below.

I voluntarily agree to take part in this research study.

________________________________ ________________________________

Participant's Name (print) **Participant's Signature/Thumbprint**

Date: ________________________________

I certify that the information provided was given in a language that was understandable to the participant.

________________________________ ________________________________

Name of Study Staff **Study Staff Signature**
Conducting Consent Discussion (print)

Date: ________________________________

________________________________ ________________________________

Witness' Name (print) **Witness' Signature/Thumbprint**
(As appropriate) Date

Date: ________________________________

FIX BARCODE HERE

STUDY COMMUNITY: ________________________

Tuberculosis Reduction through expanded antiretroviral therapy and TB screening (TREATS)

Incidence of TB Infection Cohort (15-17 years old)-Parent/Guardian Information and Consent Form

Participant Information Sheet
Please ask the study investigator or the study staff to explain any words or procedures that you do not clearly understand.

This form gives you information about the research study that the adolescent in your care is being asked to join. If you sign this form, you will be giving your permission for the adolescent in your care to take part in the study. The form describes the purpose, procedures, benefits, and risks of the research study. Please read this Information and Consent Form and ask as many questions as needed. You should not sign this form if you have any questions that have not been answered to your satisfaction. If you choose to sign this form you are giving permission for the adolescent in your care to be included in this research study.

This study is being funded by the European and Developing Countries Clinical Trials Partnership (EDCTP)

Participation is voluntary
Taking part in this study is completely voluntary, your adolescent does not have to take part if you do not want to. Access to health care from the health centres in your community will not be affected if your adolescent chooses not to take part in the study. You are also free to withdraw your adolescent from the study at any stage, without consequences to you or your family.

What is tuberculosis (TB)?
TB is an infectious airborne disease caused by bacteria (germs) which are spread through droplets from coughing or sneezing which are inhaled in into the lungs. The TB germ mainly affects the lungs but it can also affect other parts of the body. When the TB germ enters your lungs, we say you have **TB infection.** When you have TB infection, you are usually healthy and do not feel sick at all. You may have TB infection for some time which can be for some weeks up to years. Some people may have TB infection and may never feel unwell. If you start to feel unwell because of the TB germs in your lungs, we say you have **TB disease.**

Purpose of this study
The TREATS study is made up of 4 studies that will look whether the PopART intervention that has been carried out in some communities, reduced the chance of getting TB infection or developing TB disease. The PopART intervention involved HIV testing and treatment as well as screening for the symptoms that suggest your adolescent might have TB. In total fourteen (14) communities are included in this research, 8 in Zambia and 6 in South Africa.

This particular study is called the "Incidence of TB Infection Cohort". In ***[insert the name of the country]*** this study aims to recruit a total of ***[insert number of participants recruited in each site]*** young people (300 from each community) aged 15-24 years. Your adolescent has

been selected to be one of the people from your community who we are asking to take part in this study.

What will happen during this study?
If you agree to allow your adolescent take part in this study, they will need to be seen either at home, at a central site for each community, this may be the local health facility or some other clinical research site or at a mutually agreed convenient place three times: today, in one year (12 months), and in 2 years (24 months). We will contact your adolescent to remind them about their visits by either calling or sending a short text message (sms).

Today and at future visits we will:

- Ask your adolescent questions about their risk of infection with TB including about what they do, where they go and whether they have been in contact with someone who has had TB.
- Take physical measurement of height and weight
- Collect up to 15mL of blood (about 3 teaspoons) for tests including Quantiferon Plus test (to assess evidence of TB infection), HIV antibody testing and for storage for additional tests. Blood samples will be stored until all study-related testing is complete and will then be destroyed. The stored blood may be used to try to understand TB better and discover new tests for TB, by testing both your genes and looking for TB genes and other markers to see if lung and other infections are associated with becoming sick with TB.The blood samples will be securely stored both inside and outside the country. Blood collection will be performed by qualified and fully trained personnel.
- Any participant who tests QFT Plus positive at baseline will be assessed by study staff for active TB disease and counselled by qualified and trained personnel regarding their need for isoniazid preventative therapy according to national guidelines
- Ask your adolescent to provide ***[insert number of samples needed depending on site requirements]*** sputum sample if he/she has any signs or symptoms, so we can test for TB disease.
- Offer to perform an on-the-spot HIV test at each visit. ***[Insert whether a separate informed consent needs to be signed for HIV testing]***. Counselling before and after being tested will be provided by qualified and fully trained personnel.
- Any participant found to have TB or HIV at any follow up visit will receive counselling by qualified and trained personnel and be referred to the clinic for further assessment and care.

What are the possible risks or discomforts?
Your adolescent may become embarrassed, worried or anxious when learning their HIV or TB infection status. A trained staff member will help them to deal with any feelings or questions they have.

It is very rare to have any problems from having a blood test, but your adolescent may feel discomfort, dizzy, or even faint when their blood is drawn. Redness, pain, swelling, bruising may occur where the needle goes into their arm, but this is rare. The amount of blood we are taking for this study is up to 3 teaspoons and their body can very quickly replace the amount.

What are the potential benefits?
During the study your adolescent will learn whether they have been infected with TB and they will learn more about what this means and the signs and symptoms of TB disease. Your adolescent can also decide if they would like to learn their HIV status and be provided with information on where to receive treatment and care services if needed. We will be able to offer them a finger prick blood test for HIV which we can tell them the results today in about 15-20 minutes. They will also be able to ask questions about their health.

In addition, knowledge gained from this study may help reduce the spread of TB at community level and in the country. The results will help design better programs to control TB and HIV and promote better health for your adolescent and your family as well as helping with acceptance of TB as a community-wide health problem.

Are there any alternatives to participation?
If your adolescent decides not to take part in this study, we will refer them to other places where they can be screened for TB disease or receive an HIV test.

How will my confidentiality and privacy be protected?
We will do everything possible to protect your adolescent's confidentiality if they join this study. We do this by giving them a study number and any information will be labelled with this number only, so people working in the health centres and laboratories will only see a number not your adolescent's name; only the research staff will be able to link this number to their name. All records identifying your adolescent will be kept confidential, and to the extent permitted by applicable laws and regulations, will not be made publicly available. Your adolescent's personal information (name, address, phone number) will be protected by the research staff. No personal information will be included in the study or in data that will be forwarded to the sponsor or sponsor representatives. This information will not be used in any publication of information about this study. Your adolescent will be identified by a coded number in any reports of publications produced from this study (study data). Your adolescent's personal information will only be used to contact him/her if any of the results of the tests necessitates that.

People who may review your adolescent's records include ***[insert any site-specific institutions, national regulatory authorities and local research committees]***. Ethics Committees (ECs) are committees that watch over the safety and rights of research participants. All personnel accessing your adolescent's records are required to respect their confidentiality at all times. By signing this document, you are authorizing such access.

To protect their privacy, your adolescent will meet with the researcher in a private area.

Data Protection
What kind of information will be collected from you?
During this study we will collect general information such as your adolescent's gender, age, home address and employment status. Your adolescent will also be asked to provide information about the type of house they live in, tobacco and alcohol intake and how they spend their social time. Your adolescent will also be asked questions about TB and HIV. No one will be able be to recognise your adolescent in the data that will be collected. A barcode ID with their study number will be allocated to your adolescent and will be used instead of their name.

[Insert any other site-specific regulations that need to be included in this section, i.e. refer to national Protection of Personal Information Act].

How will data be recorded?

Some of the information that your adolescent gives us will be recorded on paper for example the assent form that you and your adolescent will sign, and test results. Other information like the questionnaire will be recorded electronically and will be recorded on a hand-held device. The hand-held device is securely protected by a password only known by the Research Assistant. All this information will be assigned a barcode ID so that your adolescent's confidentiality is maintained.

How will it be stored?

All paper copies that will have your adolescent's information will be kept securely in a locked cabinet in a locked room that will only be accessed by assigned study staff. All information that is recorded hand-held devices is accessed only by the Research Assistant. All electronic data will be stored on a server and will be encrypted and password protected and will only be accessible by the data manager.

All the information collected will be stored for approximately 7 years after the study has ended after which, data will be destroyed.

Who will the information be shared with?

We may share it with people who check that the study is done properly (like the independent ethics committee or review boards). The data that we collect, but not your adolescent's name or anything else that can identify them will be shared with other researchers working on the TREATS study. These include researchers working at Zambart in Zambia, Health Systems Trust in South Africa, the London School of Hygiene and Tropical Medicine, London School of Economics, Imperial College and the University of Sheffield in the UK and KNCV in the Netherlands. We will publish study results in medical journals, for meetings and on the internet for other researchers to use. Your adolescent's name or personal information will not appear in any publication.

After the study is complete copies of the data, without any details that could identify your adolescent, will be made publicly available via the internet for other researchers to use. To make sure your adolescent can never be identified we will remove information such as their name, where they live, their date of birth, the name of their community and any other data that may lead to someone being able to identify them.

What happens if your adolescent is injured by participating in this study?

It is very unlikely that anyone could be injured because of taking part in this study. However, if your adolescent is injured while taking part in this study, they will be given immediate treatment for their injuries and referred to the health facility. Your adolescent will be compensated if an injury occurs during any of the study procedures. You and your adolescent will not be giving up any of your legal rights by signing this Information and Consent Form.

All principal investigators and sites are covered by the LSHTM sponsorship insurance, and have specific Medical Malpractice Insurance to cover claims.

Will your adolescent receive any payment?

If your adolescent takes in the study, they will receive a transport refund of ***[insert amount for each site]*** for each regular study visit.

What are some reasons why your adolescent may be withdrawn from this study without your consent?

Your adolescent may be withdrawn from the study without your consent for the following reasons:

- The research study, or this part of the study, is stopped or cancelled
- The study staff feels that completing the study or this part of the study would be harmful to your adolescent or others

Persons to Contact for Problems or Questions

If you have any questions about your adolescent taking part in this research study, their rights as a research participant, or if you feel that they have experienced a research-related injury, contact:

Principle investigator's Name: *[Insert name PI]*
Research Site Address (es): *[Insert PI's address]*

Daytime telephone number (s): *[Insert PI's telephone number]*

If you have any other questions or concerns about your adolescent's rights as a research participant or want to discuss a problem, get information or offer input, you may contact:

Independent Review Board/Ethics Committee: *[Insert IRB and/or Ethics Committee]*

Address of Independent Review Board: *[Insert IRB and/or Ethics Committee's address]*

Daytime Telephone Number: *[Insert IRB and/or Ethics Committee's telephone number]*

[Insert any other required authorities to be contacted for each site]

Tuberculosis Reduction through expanded antiretroviral therapy and TB screening (TREATS)

Incidence of Infection Cohort (15-17 years old) - Parent/Guardian Information and Consent Form

STATEMENT OF CONSENT

I confirm that I am the parent or legal guardian of this adolescent

I have been given sufficient time to consider whether to allow my adolescent to take part in this study.

I understand that my adolescent taking part in the study is voluntary. I understand that they may decide not to take part or can withdraw at any time from the study without penalty or loss of benefits or treatment to which they are entitled.

I understand the research study may be stopped at any time without my or my adolescent's consent.

I have been told how long my adolescent may be in the research study and have been informed of the procedures and tests that may be performed during the research study, as well as of the possible risks and benefits. I have had an opportunity to ask questions about this research study and my questions have been answered to my satisfaction.

I understand that the information my adolescent has given will be published in reports and papers, but that my adolescent's confidentiality will be maintained and it will not be possible to identify them from any publications.

I have been informed that my adolescent's data will be shared with the partners and organisations that are working with ***[insert the name of the site]*** on this study.

I understand that confidentiality will be maintained and it will not be possible to identify me.

I understand that I do not give up my legal rights, or those of my adolescent by signing this form.

I understand that I will receive a signed and dated copy of this Participant Information and Consent Form.

If you have either read or have heard the information in this Participant Information and Consent Form, if all your questions have been answered, and if you agree that your adolescent takes part in the study, please print and sign your name and write the date on the line below.

____

Adolescent's Name (print)

________________________________ ______________________________

Parent/Guardian Name (print) **Parent/Guardian Signature/ Thumbprint**

Date: __________________________________

I certify that the information provided was given in a language that was understandable to the person giving consent.

__________________________________ _________________________________

Name of Study Staff Conducting Consent Discussion (print) **Study Staff Signature**

Date: __________________________________

__________________________________ __________________________________

Witness' Name (print) (As appropriate) Date **Witness' Signature/thumbprint**

Date: __________________________________

FIX BARCODE HERE

STUDY COMMUNITY: _________________________

Tuberculosis Reduction through expanded antiretroviral therapy and TB screening (TREATS)

Incidence of TB Infection Cohort (15-17 years old)-Information and Assent Form

Participant Information Sheet

Please ask the study investigator or the study staff to explain any words or procedures that you do not clearly understand.

This form gives you information about the research study you are being asked to join. The form describes the purpose, procedures, benefits, and risks of the research study. Please read this Information and Assent Form and ask as many questions as needed. You should not sign this form if you have any questions that have not been answered to your satisfaction. If you choose to sign this form you are giving permission to be included in this research study.

This study is being funded by the European and Developing Countries Clinical Trials Partnership (EDCTP)

Your participation is voluntary

You do not have to take part in this study if do not want to. Access to health care from the health centres in your community will not be affected if you choose not to participate in the study. You are also free to withdraw from the study at any stage, without consequences to you or your family.

What is tuberculosis (TB)?

TB is an infectious airborne disease caused by bacteria (germs) which are spread through droplets from coughing or sneezing which are inhaled in into the lungs. The TB germ mainly affects the lungs but it can also affect other parts of the body. When the TB germ enters your lungs, we say you have **TB infection.** When you have TB infection, you are usually healthy and do not feel sick at all. You may have TB infection for some time which can be for some weeks up to years. Some people may have TB infection and may never feel unwell. If you start to feel unwell because of the TB germs in your lungs, we say you have **TB disease.**

Purpose of this study

The TREATS study is made up of 4 studies that will look whether the PopART intervention that has been carried out in some communities, reduced the chance of getting TB infection or developing TB disease. The PopART intervention involved HIV testing and treatment as well as screening for the symptoms that suggest you might have TB. In total fourteen (14) communities are included in this research, 8 in Zambia and 6 in South Africa.

This particular study is called the "Incidence of TB Infection Cohort". In ***[insert the name of the country]*** this study aims to recruit a total of ***[insert number of participants recruited in each site]*** young people (300 from each community) aged 15-24 years. You have been selected to be one of the people from your community who we are asking to take part in this study.

What will happen during this study?

If you agree to take part in this study, you will need to be seen either at home, at a central site for each community, this may be the local health facility or some other clinical research site or at a mutually agreed convenient place three times: today, in one year (12 months), and in 2

years (24 months). We will contact you to remind you about your visits by either calling or sending a short text message (sms).

Today and at future visits we will:

- Ask you questions about your risk of infection with TB including what you do, where you go and whether you have been in contact with someone who has had TB.
- Take physical measurement of height and weight
- Collect up to 15mL of blood (about 3 teaspoons) for tests including Quantiferon Plus test (to assess evidence of TB infection), HIV antibody testing and for storage for additional tests. Blood samples will be stored until all study-related testing is complete and will then be destroyed. The stored blood may be used to try to understand TB better and discover new tests for TB, by testing both your genes and looking for TB genes and other markers to see if lung and other infections are associated with becoming sick with TB.The blood samples will be securely stored both inside and outside the country. Blood collection will be performed by qualified and fully trained personnel.
- Any participant who tests QFT Plus positive at baseline will be assessed by study staff for active TB disease and counselled by qualified and trained personnel regarding their need for isoniazid preventative therapy according to national guidelines
- Ask you to provide ***[insert number of samples needed depending on site requirements]*** sputum sample (s) if you have any signs or symptoms, so we can test for TB disease.
- Offer to perform an on-the-spot HIV test at each visit. ***[Insert whether a separate informed consent needs to be signed for HIV testing]***. Counselling before and after being tested will be provided by qualified and fully trained personnel.
- Any participant found to have TB or HIV at any follow up visit will receive counselling by qualified and trained personnel and be referred to the clinic for further assessment and care.

What are the possible risks or discomforts?
You may become embarrassed, worried or anxious when learning your HIV or TB infection status. A trained staff member will help you deal with any feelings or questions you have.

It is very rare to have any problems from having a blood test, but you may feel discomfort, dizzy, or even faint when your blood is drawn. Redness, pain, swelling, bruising may occur where the needle goes into your arm, but this is rare. The amount of blood we are taking for this study is up to 3 teaspoons and your body can very quickly replace the amount.

What are the potential benefits?
During the study you will learn whether you have been infected with TB and if so linked to appropriate care to prevent TB disease. Also you will learn more about what this means and the signs and symptoms of TB disease. You can also decide if you would like to learn your HIV status and be provided with information on where to receive treatment and care services if needed. We will be able to offer you a finger prick blood test for HIV which we can tell you the results today in about 15-20 minutes. You will also be able to ask questions about your health.

In addition, knowledge gained from this study may help reduce the spread of TB at community level and in the country. The results will help design better programs to control TB and HIV

and promote better health for you and your family as well as helping with acceptance of TB as a community-wide health problem.

Are there any alternatives to participation?
If you decide not to take part in this study, we will refer you to other places where you can be screened for TB disease or receive an HIV test.

How will my confidentiality and privacy be protected?
We will do everything possible to protect your confidentiality if you join this study. We do this by giving you a study number (for example, 1234782) and any information will be labelled with this number only, so people working in the health centres and laboratories will only see a number not your name; only the research staff will be able to link this number to your name. All records identifying you will be kept confidential, and to the extent permitted by applicable laws and regulations, will not be made publicly available. Your personal information (name, address, phone number) will be protected by the research staff. No personal information will be included in the study or in the data that will be forwarded to the sponsor or sponsor representatives. This information will not be used in any publication of information about this study. You will be identified by a coded number in any reports of publications produced from this study (study data). Your personal information will only be used to contact you if any of the results of the tests necessitates that.

People who may review your records include ***[insert any site-specific institutions, national regulatory authorities and local research committees]***. Ethics Committees (ECs) are committees that watch over the safety and rights of research participants. All personnel accessing your records are required to respect your confidentiality at all times. By signing this document, you are authorizing such access.

To protect your privacy, you will meet with the researcher in a private area.

Data Protection
What kind of information will be collected from you?
During this study we will collect general information such as your gender, age, home address and employment status. You will also be asked to provide information about the type of house you live in, tobacco and alcohol intake & how you spend your social time. You will also be asked questions about TB and HIV. No one will be able be to recognise you in all of the data that will be collected. A barcode ID with your study number will be allocated to you and will be used instead of your name.

[Insert any other site-specific regulations that need to be included in this section, i.e. refer to national Protection of Personal Information Act].

How will data be recorded?
Some of the information that you give us will be recorded on paper for example the assent form that you will sign, and test results. Other information like the questionnaire will be recorded electronically and will be recorded on a hand-held device. The hand-held device is securely protected by a password only known by the Research Assistant. All this information will be assigned a barcode ID so that your confidentiality is maintained.

How will it be stored?

All paper copies that will have your information will be kept securely in a locked cabinet in a locked room that will only be accessed by assigned study staff. All information that is recorded hand-held devices is accessed only by the Research Assistant. All electronic data will stored on a server and will be encrypted and password protected and will only be accessible by the data manager.

All the information collected will be stored for approximately 7 years after the study has ended after which, data will be destroyed.

Who will the information be shared with?
The data that we collect, but not your name or anything else that can identify you will be shared with other researchers working on the TREATS study. These include researchers working at Zambart in Zambia, health Systems Trust in South Africa, the London School of Hygiene and Tropical Medicine, London School of Economics, Imperial College and the University of Sheffield in the UK and KNCV in the Netherlands. We will publish study results in medical journals, for meetings and on the internet for other researchers to use. Your name or personal information will not appear in any publication.

After the study is complete copies of the data, without any details that could identify you, will be made publicly available via the internet for other researchers to use. To make sure you can never be identified we will remove information such as your name, where you live, your date of birth, the name of your community and any other data that may lead to someone being able to identify you

What happens if I am injured by participating in this study?
It is very unlikely that you could be injured because of taking part in this study. However, if you are injured while taking part in this study, you will be given immediate treatment for your injuries and referred to the health facility. You will be compensated if an injury occurs during any of the study procedures. You will not be giving up any of your legal rights by signing this Information and Consent Form.

All principal investigators and sites are covered by the LSHTM sponsorship insurance, and have specific Medical Malpractice Insurance to cover claims.

Will I receive any payment?
If you take part in this study, you will be refunded your transport ***costs [insert amount for each site]*** for each regular study visit.

What are some reasons why I may be withdrawn from this study without my consent?
You may be withdrawn from the study without your consent for the following reasons:

- The research study, or this part of the study is stopped or canceled
- The study staff feels that completing the study or this part of the study would be harmful to you or others

Persons to Contact for Problems or Questions
If you have any questions about taking part in this research study, your rights as a research participant, or if you feel that you have experienced a research-related injury, contact:

Principle investigator's Name: ***[Insert name PI]***

Research Site Address (es): ***[Insert PI's address]***

Daytime telephone number (s): ***[Insert PI's telephone number]***

If you have any other questions or concerns about your rights as a research participant or want to discuss a problem, get information or offer input, you may contact:

Independent Review Board/Ethics Committee: ***[Insert IRB and/or Ethics Committee]***

Address of Independent Review Board: ***[Insert IRB and/or Ethics Committee's address]***

Daytime Telephone Number: ***[Insert IRB and/or Ethics Committee's telephone number]***

[Insert any other required authorities to be contacted for each site]

Tuberculosis Reduction through expanded antiretroviral therapy and TB screening (TREATS)

Incidence of Infection Cohort (15-17 years old)-Assent Form

STATEMENT OF CONSENT

I have been given sufficient time to consider whether to take part in this study.

My taking part in this research study is voluntary. I understand that I may decide not to take part or can withdraw at any time from the study without penalty or loss of benefits or treatment to which I am entitled.

I understand the research study may be stopped at any time without my consent.

I have been told how long I may be in the research study and have been informed of the procedures and tests that may be performed during the research study, as well as of the possible risks and benefits. I have had an opportunity to ask questions about this research study and my questions have been answered to my satisfaction.

I understand that the information I have given will be published in reports and papers, but that confidentiality will be maintained and it will not be possible to identify me from any publications.

I have been informed that my data will be shared with the partners and organisations that are working with ***[insert name of the site***] on this study.

I understand that confidentiality will be maintained and it will not be possible to identify me.

I understand that I do not give up my legal rights by signing this form.

I understand that I will receive a signed and dated copy of this Participant Information and Assent Form.

If you have either read or have heard the information in this Participant Information and Assent Form, if all your questions have been answered, and if you agree to take part in the study, please print and sign your name and write the date on the line below.

I voluntarily agree to take part in this research study.

________________________________	________________________________
**Adolescent's name (print)**	**Adolescent's signature/Thumbprint**

Adolescent's age (years): __________

Date: ________________________________

Remember to obtain signed consent from parent/guardian
I certify that the information provided was given in a language that was understandable to the participant.

________________________________	________________________________
**Name of Study Staff Conducting Consent Discussion (print)**	**Study Staff Signature**

Date: ________________________________

________________________________	________________________________
**Witness' Name (print) (As appropriate) Date**	**Witness' Signature /Thumbprint**

Date: ________________________________

FIX BARCODE HERE

STUDY COMMUNITY: _______________________

APPENDIX II - SAMPLE INFORMED CONSENT FORM – PREVALENCE SURVEY

Tuberculosis Reduction through expanded antiretroviral therapy and TB screening (TREATS)

Prevalence Survey (≥18years old)-Information and Consent Form

Participant Information Sheet
Please ask the study staff to explain any words or procedures that you do not clearly understand.

This form gives you information about the research study you are being asked to join. The form describes the purpose, procedures, benefits, and risks of the research study. Please read this Information and Consent Form and ask as many questions as needed. You should not sign this form if you have any questions that have not been answered to your satisfaction. If you choose to sign this form you are giving permission to be included in this research study.

This study is being funded by the European and Developing Countries Clinical Trials Partnership (EDCTP)

Your participation is voluntary
You do not have to take part in this study if you do not want to. Access to health care from the health centres in your community will not be affected if you choose not to participate in the study. You are also free to withdraw from the study at any stage, without consequences to you or your family.

What is tuberculosis (TB)?
TB is an infectious airborne disease caused by bacteria (germs) which are spread through droplets from coughing or sneezing which are inhaled in into the lungs. The TB germ mainly affects the lungs but it can also affect other parts of the body. When the TB germ enters your lungs, we say you have **TB infection.** When you have TB infection, you are usually healthy and do not feel sick at all. You may have TB infection for some time which can be for some weeks up to years. Some people may have TB infection and may never feel unwell. If you start to feel unwell because of the TB germs in your lungs, we say you have **TB disease.**

Purpose of this study
The TREATS study is made up of 4 studies that will look whether the PopART study that has been carried out in 21 communities, 12 in Zambia and 9 in South Africa, reduced the chance of getting TB infection or developing TB disease. The PopART intervention involved HIV testing and treatment as well as screening for the symptoms that suggest you might have TB.

This particular study is called the "Prevalence Survey". In ***[insert the name of the country]*** this study aims to recruit a total of ***[insert number of participants recruited in each site]*** people 15 years and older from this community to measure how much TB disease there is in this community. You have been selected to be one of the people from your community who we are asking to take part in this study.

What will happen during this study?

If you take part in this study, we will ask you some questions about you, your family and your health, as well as about risk factors for TB disease. We will then ask you to have a chest X-ray taken at our mobile X-ray machine. This is a very simple procedure and it is very quick, it takes around 5-10 minutes and you will not be charged. The chest X-ray picture of your lungs will be seen on a computer and this can tell us if there might be a chance you have TB disease. If you have signs and symptoms of TB or your chest X-ray does not look normal we will ask you to produce ***[insert number of samples needed depending on site requirements]*** sputum sample (s) to test for TB. Your sputum sample (s) will be tested on the spot for TB and the result will be available in 1 or 2 days. In addition we may ask for additional sputum samples to be sent to the laboratory for additional testing for TB. This test can take up to 8 weeks and so if this is the case we will tell you and will come to find you or contact you over the telephone to give you the results when they are ready.

If you are found to have TB, we will contact you and notify your local health facility and refer you there for treatment. If you do not have TB but your chest X-ray shows that you could have other disease we will ask a clinician to discuss this with you and provide you with a referral to a health facility for further investigation or treatment.

We will also request a blood sample of up to 10mls of blood (2 teaspoons) from some individuals to use for new tests to detect TB.

If you agree, we will perform an on-the-spot HIV test. **[*Insert whether a separate informed consent needs to be signed for HIV testing*].** We will provide counselling before and after being tested by qualified counsellors .

If you know that you have HIV, or we test you and find that you have HIV, we will ask for a small sample of blood to be taken using a finger-prick so that we can look at the HIV virus to see if any treatment you are taking has reduced the amount of virus in your blood.We will look at the different types of virus found in blood samples of different people in the community who are living with HIV. In science we call this Phylogenetics. This kind of research will help the PopART research team to understand better how the trial affected the spread of HIV and other viruses in your community

If you are found to have TB or HIV we will provide counselling by qualified counsellors and medical staff and refer you to the clinic for further assessment and care.

What are the possible risks or discomforts?
You may become embarrassed, worried or anxious when learning your HIV or TB infection status. A trained staff member will help you deal with any feelings or questions you have.

Risks associated with chest X-ray are minimal as the radiation exposure from these new machines is very low, and the x-rays are directed at the chest. However, if you are pregnant or have any concerns about the effects that the X-ray may have on your health or on that of your unborn baby please discuss it in detail with the radiographer and make sure all your questions are answered. You can still continue to take part in the study even if you choose not to have the chest X-ray.

Risks associated with blood sampling may be that you will have a small bruise on the site of the blood draw. Occasionally some people may feel a bit faint when blood is drawn but we will try to avoid this by drawing blood when you are sitting comfortably.

What are the potential benefits?

During the study you will learn whether you have TB disease and if so will be linked to care to cure the TB disease. Also you will learn more about the signs and symptoms of TB disease and have an X-ray of your lungs taken free of charge. You will have the opportunity to learn your HIV status and be provided with information on where to receive treatment and care services if needed. You will also be able to ask questions about your health.

In addition, the results will help design better programs to control TB and HIV and promote better health for you and your family as well as helping with acceptance of TB as a community-wide health problem.

Are there any alternatives to participation?

If you decide not to take part in this study, we will refer you to other places where you can be screened for TB disease or receive an HIV test.

How will my confidentiality and privacy be protected?

We will do everything possible to protect your confidentiality if you join this study.
To protect your privacy, you will meet with the researcher in a private area.

What kind of information will be collected from you?

During this study we will collect general information such as your gender, age, home address and employment status. You will also be asked to provide information about the type of house you live in, tobacco and alcohol intake. You will also be asked questions about TB and HIV. No one will be able to recognise you in all of the data that will be collected. A barcode ID with your study number will be allocated to you and will be used instead of your name.

[Insert any other site-specific regulations that need to be included in this section, i.e. refer to national Protection of Personal Information Act].

How will data be recorded?

Some of the information that you give us will be recorded on paper for example the consent form that you will sign, and test results. Other information like the questionnaire will be recorded electronically and will be recorded on a hand-held device. The hand-held device is securely protected by a password only known by the Research Assistant. All this information will be assigned a barcode ID so that your confidentiality is maintained.

How will it be stored?

All paper copies that will have your information will be kept securely in a locked cabinet in a locked room that will only be accessed by assigned study staff. All information that is recorded in hand-held devices is accessed only by the Research Assistant. All electronic data will be stored on a server and will be encrypted and password protected and will only be accessible by the data manager.

All the information collected will be stored for approximately 7 years after the study has ended after which, data will be destroyed.

Who will the information be shared with?
We may share it with people who check that the study is done properly (like the independent ethics committee or review boards). The data that we collect, but not your name or anything else that can identify you will be shared with other researchers working on the TREATS study. These include researchers working at Zambart in Zambia, health Systems Trust in South Africa, the London School of Hygiene and Tropical Medicine, London School of Economics, University of Oxford, Imperial College and the University of Sheffield in the UK and KNCV in the Netherlands. We will publish study results in medical journals, for meetings and on the internet for other researchers to use. Your name or personal information will not appear in any publication.

After the study is complete copies of the data, without any details that could identify you, will be made publicly available via the internet for other researchers to use. To make sure you can never be identified we will remove information such as your name, where you live, your date of birth, the name of your community and any other data that may lead to someone being able to identify you.Some members of the study team may revisit you in the future to ask some follow up questions about the results of the tests you had, the treatment that you received or about other information provided to us in the course of this study.

What happens if I am injured by participating in this study?
It is very unlikely that you could be injured because of taking part in this study. However, if you are injured while taking part in this study, you will be given immediate treatment for your injuries and referred to the health facility. You will be compensated if an injury occurs during any of the study procedures. You will not be giving up any of your legal rights by signing this Information and Consent Form.

All principal investigators and sites are covered by the LSHTM sponsorship insurance and have Medical Malpractice Insurance to cover claims.

What are some reasons why I may be withdrawn from this study without my consent?
You may be withdrawn from the study without your consent for the following reasons:

- The research study, or this part of the study is stopped or cancelled
- The study staff feels that completing the study or this part of the study would be harmful to you or others

Persons to Contact for Problems or Questions
If you have any questions about taking part in this research study, your rights as a research participant, or if you feel that you have experienced a research-related injury, contact:

Principle investigator's Name: ***[Insert name PI]***

Research Site Address (es): ***[Insert PI's address]***

Daytime telephone number (s): ***[Insert PI's telephone number]***

If you have any other questions or concerns about your rights as a research participant or want to discuss a problem, get information or offer input, you may contact:

Independent Review Board/Ethics Committee: ***[Insert IRB and/or Ethics Committee]***

Address of Independent Review Board: ***[Insert IRB and/or Ethics Committee's address]***

Daytime Telephone Number: ***[Insert IRB and/or Ethics Committee's telephone number]***

[Insert any other required authorities to be contacted for each site]

Tuberculosis Reduction through expanded antiretroviral therapy and TB screening (TREATS)

Prevalence Survey (≥18years old)-

STATEMENT OF CONSENT

I have been given sufficient time to consider whether to take part in this study.

My taking part in this research study is voluntary. I understand that I may decide not to take part or can withdraw at any time from the study without penalty or loss of benefits or treatment to which I am entitled.
I understand the research study may be stopped at any time without my consent.

I have been informed of the procedures and tests that may be performed during the research study, as well as of the possible risks and benefits. I have had an opportunity to ask questions about this research study and my questions have been answered to my satisfaction.

I understand that the information I have given will be published in reports and papers, but that confidentiality will be maintained and it will not be possible to identify me from any publications.

I have been informed that my data will be shared with the partners and organisations that are working ***[insert name of the site]*** on this study.

I understand that I do not give up my legal rights by signing this form.

I understand that I will receive a signed and dated copy of this Participant Information and Consent Form.

If you have either read or have heard the information in this Participant Information and Consent Form, if all your questions have been answered, and if you agree to take part in the study, please print and sign your name and write the date on the line below.

I voluntarily agree to take part in this research study

________________________________ ________________________________

Participant's Name (print) **Participant's Signature/Thumbprint**

Date: ________________________________

I certify that the information provided was given in a language that was understandable to the participant.

________________________________ ________________________________

Name of Study Staff **Study Staff Signature**
Conducting Consent Discussion (print)

Date: ________________________________

________________________________ ________________________________

Witness' Name (print) **Witness' Signature**
(As appropriate) Date

Date: ________________________________

FIX BARCODE HERE

STUDY COMMUNITY: ________________________

Tuberculosis Reduction through expanded antiretroviral therapy and TB screening (TREATS)

Prevalence Survey (15-17years old)-Information and Assent Form

Participant Information Sheet

Please ask the study staff to explain any words or procedures that you do not clearly understand.

This form gives you information about the research study you are being asked to join. The form describes the purpose, procedures, benefits, and risks of the research study. Please read this Information and Consent Form and ask as many questions as needed. You should not sign this form if you have any questions that have not been answered to your satisfaction. If you choose to sign this form you are giving permission to be included in this research study.

This study is being funded by the European and Developing Countries Clinical Trials Partnership (EDCTP)

Your participation is voluntary

You do not have to take part in this study if you do not want to. Access to health care from the health centres in your community will not be affected if you choose not to participate in the study. You are also free to withdraw from the study at any stage, without consequences to you or your family.

What is tuberculosis (TB)?

TB is an infectious airborne disease caused by bacteria (germs) which are spread through droplets from coughing or sneezing which are inhaled in into the lungs. The TB germ mainly affects the lungs but it can also affect other parts of the body. When the TB germ enters your lungs, we say you have **TB infection.** When you have TB infection, you are usually healthy and do not feel sick at all. You may have TB infection for some time which can be for some weeks up to years. Some people may have TB infection and may never feel unwell. If you start to feel unwell because of the TB germs in your lungs, we say you have **TB disease.**

Purpose of this study

The TREATS study is made up of 4 studies that will look whether the PopART study that has been carried out in 21 communities, 12 in Zambia and 9 in South Africa, reduced the chance of getting TB infection or developing TB disease. The PopART intervention involved HIV testing and treatment as well as screening for the symptoms that suggest you might have TB.

This particular study is called the "Prevalence Survey". In ***[insert the name of the country]*** this study aims to recruit a total of ***[insert number of participants recruited in each site]*** people 15 years and older from this community to measure how much TB disease there is in this community. You have been selected to be one of the people from your community who we are asking to take part in this study.

What will happen during this study?

If you take part in this study, we will ask you some questions about you, your family and your health, as well as about risk factors for TB disease. We will then ask you to have a chest X-ray taken at our mobile X-ray machine. This is a very simple procedure and it is very quick, it takes

around 5-10 minutes and you will not be charged. The chest X-ray picture of your lungs will be seen on a computer and this can tell us if there might be a chance you have TB disease. If you have signs and symptoms of TB or your chest X-ray does not look normal we will ask you to produce ***[insert number of samples needed depending on site requirements]*** sputum sample (s) to test for TB. Your sputum sample (s) will be tested on the spot for TB and the result will be available in 1 or 2 days. In addition we may ask for additional sputum samples to be sent to the laboratory for additional testing for TB. This test can take up to 8 weeks and so if this is the case we will tell you and will come to find you or contact you over the telephone to give you the results when they are ready.

If you are found to have TB, we will contact you and notify your local health facility and refer you there for treatment. If you do not have TB but your chest X-ray shows that you could have other disease we will ask a clinician to discuss this with you and provide you with a referral to a health facility for further investigation or treatment.

We will also request a blood sample of up to 10mls of blood (2 teaspoons) from some individuals to use for new tests to detect TB.

If you agree, we will perform an on-the-spot HIV test. ***[Insert whether a separate informed consent needs to be signed for HIV testing]***. We will provide counselling before and after being tested by qualified counsellors.

If you know that you have HIV, or we test you and find that you have HIV, we will ask for a small sample of blood to be taken using a finger-prick so that we can look at the HIV virus to see if any treatment you are taking has reduced the amount of virus in your blood.We will look at the different types of virus found in blood samples of different people in the community who are living with HIV. In science we call this Phylogenetics. This kind of research will help the PopART research team to understand better how the trial affected the spread of HIV and other viruses in your community.

If you are found to have TB or HIV we will provide counselling by qualified counsellors and medical staff and refer you to the clinic for further assessment and care.

What are the possible risks or discomforts?
You may become embarrassed, worried or anxious when learning your HIV or TB infection status. A trained staff member will help you deal with any feelings or questions you have.

Risks associated to the chest X-ray are minimal as the radiation exposure from these new machines is very low, and the x-rays are directed at the chest. However, if you are pregnant or have any concerns about the effects that the X-ray may have on your health or on that of your unborn baby please discuss it in detail with the radiographer and make sure all your questions are answered. You can still continue to take part in the study even if you choose not to have the chest X-ray.

Risks associated with blood sampling may be that you will have a small bruise on the site of the blood draw. Occasionally some people may feel a bit faint when blood is drawn but we will try to avoid this by drawing blood when you are sitting comfortably.

What are the potential benefits?
During the study you will learn whether you have TB disease and if so you will be linked to care to cure the TB disease. Also you will learn more about the signs and symptoms of TB disease and have an X-rayof your lungs taken free of charge. You will have the opportunity to learn your HIV status and be provided with information on where to receive treatment and care services if needed.. You will also be able to ask questions about your health.

In addition, the results will help design better programs to control TB and HIV and promote better health for you and your family as well as helping with acceptance of TB as a community-wide health problem.

Are there any alternatives to participation?
If you decide not to take part in this study, we will refer you to other places where you can be screened for TB disease or receive an HIV test.

How will my confidentiality and privacy be protected?
We will do everything possible to protect your confidentiality if you join this study.
To protect your privacy, you will meet with the researcher in a private area.

<u>What kind of information will be collected from you?</u>
During this study we will collect general information such as your gender, age, home address and employment status. You will also be asked to provide information about the type of house you live in, tobacco and alcohol intake. You will also be asked questions about TB and HIV. No one will be able to recognise you in all of the data that will be collected. A barcode ID with your study number will be allocated to you and will be used instead of your name.

[Insert any other site-specific regulations that need to be included in this section, i.e. refer to national Protection of Personal Information Act].

<u>How will data be recorded?</u>
Some of the information that you give us will be recorded on paper for example the consent form that you will sign, and test results. Other information like the questionnaire will be recorded electronically and will be recorded on a hand-held device. The hand-held device is securely protected by a password only known by the Research Assistant. All this information will be assigned a barcode ID so that your confidentiality is maintained.

<u>How will it be stored?</u>
All paper copies that will have your information will be kept securely in a locked cabinet in a locked room that will only be accessed by assigned study staff. All information that is recorded hand-held devices is accessed only by the Research Assistant. All electronic data will stored on a server and will be encrypted and password protected and will only be accessible by the data manager.

All the information collected will be stored for approximately 7 years after the study has ended after which, data will be destroyed.

<u>Who will the information be shared with?</u>
We may share it with people who check that the study is done properly (like the independent ethics committee or review boards). The data that we collect, but not your name or anything

else that can identify you will be shared with other researchers working on the TREATS study. These include researchers working at Zambart in Zambia, health Systems Trust in South Africa, the London School of Hygiene and Tropical Medicine, London School of Economics, University of Oxford, Imperial College and the University of Sheffield in the UK and KNCV in the Netherlands. We will publish study results in medical journals, for meetings and on the internet for other researchers to use. Your name or personal information will not appear in any publication.

After the study is complete copies of the data, without any details that could identify you, will be made publicly available via the internet for other researchers to use. To make sure you can never be identified we will remove information such as your name, where you live, your date of birth, the name of your community and any other data that may lead to someone being able to identify you.

Some members of the study team may revisit you in the future to ask some follow up questions about the results of the tests you had, the treatment that you received or about other information provided to us in the course of this study

What happens if I am injured by participating in this study?
It is very unlikely that you could be injured because of taking part in this study. However, if you are injured while taking part in this study, you will be given immediate treatment for your injuries and referred to the health facility. You will be compensated if an injury occurs during any of the study procedures. You will not be giving up any of your legal rights by signing this Information and Consent Form.

All principal investigators and sites are covered by the LSHTM sponsorship insurance and have specific Medical Malpractice Insurance to cover claims.

What are some reasons why I may be withdrawn from this study without my consent?
You may be withdrawn from the study without your consent for the following reasons:

- The research study, or this part of the study is stopped or cancelled
- The study staff feels that completing the study or this part of the study would be harmful to you or others

Persons to Contact for Problems or Questions
If you have any questions about taking part in this research study, your rights as a research participant, or if you feel that you have experienced a research-related injury, contact:

Principle investigator's Name: ***[Insert name PI]***

Research Site Address (es): ***[Insert PI's address]***

Daytime telephone number (s): ***[Insert PI's telephone number]***

If you have any other questions or concerns about your rights as a research participant or want to discuss a problem, get information or offer input, you may contact:

Independent Review Board/Ethics Committee: ***[Insert IRB and/or Ethics Committee]***

Address of Independent Review Board: ***[Insert IRB and/or Ethics Committee's address*]**

Daytime Telephone Number: ***[Insert IRB and/or Ethics Committee's telephone number]***

[Insert any other required authorities to be contacted for each site]

Tuberculosis Reduction through expanded antiretroviral therapy and TB screening (TREATS)

Prevalence Survey (15-17years old)- Information and Assent Form

STATEMENT OF CONSENT

I have been given sufficient time to consider whether to take part in this study.

My taking part in this research study is voluntary. I understand that I may decide not to take part or can withdraw at any time from the study without penalty or loss of benefits or treatment to which I am entitled.
I understand the research study may be stopped at any time without my consent.

I have been informed of the procedures and tests that may be performed during the research study, as well as of the possible risks and benefits. I have had an opportunity to ask questions about this research study and my questions have been answered to my satisfaction.

I understand that the information I have given will be published in reports and papers, but that confidentiality will be maintained and it will not be possible to identify me from any publications.

I have been informed that my data will be shared with the partners and organisations that are working ***[insert name of the site]*** on this study.

I understand that I do not give up my legal rights by signing this form.

I understand that I will receive a signed and dated copy of this Participant Information and Consent Form.

If you have either read or have heard the information in this Participant Information and Consent Form, if all your questions have been answered, and if you agree to take part in the study, please print and sign your name and write the date on the line below.

I voluntarily agree to take part in this research study

_________________________________	_________________________________
**Adolescent's Name (print)**	**Adolescent's Signature/Thumbprint**

Date: _________________________________

Remember to obtain signed consent from parent/guardian

I certify that the information provided was given in a language that was understandable to the participant.

_________________________________	_________________________________
**Name of Study Staff**<br>**Conducting Consent Discussion (print)**	**Study Staff Signature**

Date: _________________________________

_________________________________	_________________________________
**Witness' Name (print)**<br>**(As appropriate) Date**	**Witness' Signature/Thumbprint**

Date: _________________________________

FIX BARCODE HERE

STUDY COMMUNITY: ________________________

Tuberculosis Reduction through expanded antiretroviral therapy and TB screening (TREATS)

Prevalence Survey (15-17years old)-Parent/Guardian Information and Consent Form

Participant Information Sheet

Please ask the study staff to explain any words or procedures that you do not clearly understand.

This form gives you information about the research study that the adolescent in your care is being asked to join. If you sign this form, you will be giving your permission for the adolescent in your care to take part in the study. The form describes the purpose, procedures, benefits, and risks of the research study. Please read this Information and Consent Form and ask as many questions as needed. You should not sign this form if you have any questions that have not been answered to your satisfaction. If you choose to sign this form you are giving permission for the adolescent in your care to be included in this research study.

This study is being funded by the European and Developing Countries Clinical Trials Partnership (EDCTP)

Participation is voluntary

Taking part in this study is completely voluntary. Your adolescent does not have to take part in this study if you do not want to. Access to health care from the health centres in your community will not be affected if your adolescent chooses not to participate in the study. You are also free to withdraw your adolescent from the study at any stage, without consequences to you or your family.

What is tuberculosis (TB)?

TB is an infectious airborne disease caused by bacteria (germs) which are spread through droplets from coughing or sneezing which are inhaled in into the lungs. The TB germ mainly affects the lungs but it can also affect other parts of the body. When the TB germ enters your lungs, we say you have **TB infection.** When you have TB infection, you are usually healthy and do not feel sick at all. You may have TB infection for some time which can be for some weeks up to years. Some people may have TB infection and may never feel unwell. If you start to feel unwell because of the TB germs in your lungs, we say you have **TB disease.**

Purpose of this study

The TREATS study is made up of 4 studies that will look whether the PopART studythat has been carried out in 21 communities, 12 in Zambia and 9 in South Africa, reduced the chance of getting TB infection or developing TB disease. The PopART intervention involved HIV testing and treatment as well as screening for the symptoms that suggest your adolescent might have TB.

This particular study is called the "Prevalence Survey". In ***[insert the name of the country] this study aims to recruit a total of [insert number of participants recruited in each site]*** people 15 years and older from this community to measure how much TB disease there is in this community. Your adolescent has been selected to be one of the people from your community who we are asking to take part in this study.

What will happen during this study?
If your adolescent takes part in this study, we will ask them some questions about them, your family and their health, as well as about risk factors for TB disease. We will then ask your adolescent to have a chest X-ray taken at our mobile X-ray machine. This is a very simple procedure and it is very quick, it takes around 5-10 minutes and they will not be charged. The chest X-ray picture of your adolescent's lungs will be seen on a computer and this can tell us if there might be a chance they have TB disease. If your adolescent has signs and symptoms of TB or their chest X-ray does not look normal we will ask your adolescent to produce ***[insert number of samples needed depending on site requirements]*** sputum sample (s) to test for TB. Your adolescent's sputum sample (s) will be tested on the spot for TB and the result will be available in 1 or 2 days. In addition we may ask your adolescent for additional sputum samples to be sent to the laboratory for additional testing for TB. This test can take up to 8 weeks and so if this is the case we will tell your adolescent and will come to find them or contact them over the telephone to give them the results when they are ready.

If your adolescent is found to have TB, we will contact them and notify your local health facility and refer them there for treatment. If your adolescent does not have TB but their chest X-ray shows that they could have other disease we will ask a clinician to discuss this with you and your adolescent and provide your adolescent with a referral to a health facility for further investigation or treatment.

We will also request a blood sample of up to 10mls of blood (2 teaspoons) from some individuals to use for new tests to detect TB.

If you agree your adolescent to take part in the study, we will perform an on-the-spot HIV test. ***[Insert whether a separate informed consent needs to be signed for HIV testing]***. We will provide counselling before and after being tested by qualified counsellors.

If your adolescent knows that they have HIV, or we test them and find that they have HIV, we will ask them for a small sample of blood to be taken using a finger-prick so that we can look at the HIV virus to see if any treatment they are taking has reduced the amount of virus in their blood.We will look at the different types of virus found in blood samples of different people in the community who are living with HIV. In science we call this Phylogenetics. This kind of research will help the PopART research team to understand better how the trial affected the spread of HIV and other viruses in the community.

If your adolescent is found to have TB or HIV we will provide counselling by qualified and medical staff and refer your adolescentto the clinic for further assessment and care.

What are the possible risks or discomforts?
Your adolescent may become embarrassed, worried or anxious when learning their HIV or TB infection status. A trained staff member will help them deal with any feelings or questions they have.

Risks associated with chest X-ray are minimal as the radiation exposure from these new machines is very low, and the x-rays are directed at the chest. However, if your adolescent is pregnant or have any concerns about the effects that the X-ray may have on their health or on that of their unborn baby please discuss it in detail with the radiographer and make sure all your

questions are answered. Your adolescent can still continue to take part in the study even if they choose not to have the chest X-ray.

Risks associated with blood sampling may be that your adolescent will have a small bruise on the site of the blood draw. Occasionally some people may feel a bit faint when blood is drawn but we will try to avoid this by drawing blood when someone is sitting comfortably.

What are the potential benefits?
During the study your adolescent will learn whether they have TB disease and if so they will be linked to care to cure the TB disease. Also your adolescent will learn more about the signs and symptoms of TB disease and have an X-ray of their lungs taken free of charge. Your adolescent will have the opportunity to learn their HIV status and be provided with information on where to receive treatment and care services if needed. They will also be able to ask questions about their health.

In addition, the results will help design better programs to control TB and HIV and promote better health for your adolescent and your family as well as helping with acceptance of TB as a community-wide health problem.

Are there any alternatives to participation?
If your adolescent decides not to take part in this study, we will refer them to other places where they can be screened for TB disease or receive an HIV test.

How will my confidentiality and privacy be protected?
We will do everything possible to protect your adolescent's confidentiality if they join this study.
To protect their privacy, your adolescent will meet with the researcher in a private area.

<u>What kind of information will be collected from your adolescent?</u>
During this study we will collect general information such as your adolescent's gender, age, home address and employment status. Your adolescent will also be asked to provide information about the type of house they live in, tobacco and alcohol intake. Your adolescent will also be asked questions about TB and HIV. No one will be able to recognise your adolescent in all of the data that will be collected. A barcode ID with your study number will be allocated to your adolescent and will be used instead of their name.

[Insert any other site-specific regulations that need to be included in this section, i.e. refer to national Protection of Personal Information Act].

<u>How will data be recorded?</u>
Some of the information that your adolescent give us will be recorded on paper for example the assent form that you and your adolescent will sign, and test results. Other information like the questionnaire will be recorded electronically and will be recorded on a hand-held device. The hand-held device is securely protected by a password only known by the Research Assistant. All this information will be assigned a barcode ID so that your adolescent's confidentiality is maintained.

<u>How will it be stored?</u>

All paper copies that will have your adolescent's information will be kept securely in a locked cabinet in a locked room that will only be accessed by assigned study staff. All information that is recorded in hand-held devices is accessed only by the Research Assistant. All electronic data will be stored on a server and will be encrypted and password protected and will only be accessible by the data manager.

All the information collected will be stored for approximately 7 years after the study has ended after which, data will be destroyed.

Who will the information be shared with?
We may share it with people who check that the study is done properly (like the independent ethics committee or review boards). The data that we collect, but not your adolescent's name or anything else that can identify them will be shared with other researchers working on the TREATS study. These include researchers working at Zambart in Zambia, health Systems Trust in South Africa, the London School of Hygiene and Tropical Medicine, London School of Economics, University of Oxford, Imperial College and the University of Sheffield in the UK and KNCV in the Netherlands. We will publish study results in medical journals, for meetings and on the internet for other researchers to use. Your adolescent's name or personal information will not appear in any publication.

After the study is complete copies of the data, without any details that could identify your adolescent, will be made publicly available via the internet for other researchers to use. To make sure your adolescent can never be identified we will remove information such as their name, where they live, their date of birth, the name of their community and any other data that may lead to someone being able to identify them.

Some members of the study team may revisit your adolescent in the future to ask some follow up questions about the results of the tests they had, the treatment that they received or about other information provided to us in the course of this study

What happens if your adolescent is injured by participating in this study?
It is very unlikely that anyone could be injured because of taking part in this study. However, if your adolescent is injured while taking part in this study, they will be given immediate treatment for your injuries and referred to the health facility. Your adolescent will be compensated if an injury occurs during any of the study procedures. You and your adolescent will not be giving up any of your legal rights by signing this Information and Consent Form.

All principal investigators and sites are covered by LSHTM sponsorship insurance and have Medical Malpractice Insurance to cover claims.

What are some reasons why I may be withdrawn from this study without my consent?
Your adolescent may be withdrawn from the study without your consent for the following reasons:

- The research study, or this part of the study is stopped or cancelled
- The study staff feels that completing the study or this part of the study would be harmful to your adolescent or others

Persons to Contact for Problems or Questions

If you have any questions about your adolescent taking part in this research study, their rights as a research participant, or if you feel that they have experienced a research-related injury, contact:

Principle investigator's Name: ***[Insert name PI]***

Research Site Address (es): ***[Insert PI's address]***

Daytime telephone number (s): ***[Insert PI's telephone number]***

If you have any other questions or concerns about your adolescent's rights as a research participant or want to discuss a problem, get information or offer input, you may contact:

Independent Review Board/Ethics Committee: ***[Insert IRB and/or Ethics Committee]***

Address of Independent Review Board: ***[Insert IRB and/or Ethics Committee's address]***

Daytime Telephone Number: ***[Insert IRB and/or Ethics Committee's telephone number]***

[Insert any other required authorities to be contacted for each site]

Tuberculosis Reduction through expanded antiretroviral therapy and TB screening (TREATS)

Prevalence Survey (15-17yrs) - Parent/Guardian Information and Consent Form

STATEMENT OF CONSENT

I confirm that I am the parent or legal guardian of this adolescent

I have been given sufficient time to consider whether to allow my adolescent to take part in this study.

I understand that my adolescent taking part in this research study is voluntary. I understand that they may decide not to take part or can withdraw at any time from the study without penalty or loss of benefits or treatment to which they are entitled.
I understand the research study may be stopped at any time without my or my adolescent's consent.

I d have been informed of the procedures and tests that may be performed during the research study, as well as of the possible risks and benefits. I have had an opportunity to ask questions about this research study and my questions have been answered to my satisfaction.

I understand that the information my adolescent has given will be published in reports and papers, but that confidentiality will be maintained and it will not be possible to identify them from any publications.

I have been informed that my adolescent's data will be shared with the partners and organisations that are working with ***[insert name of the site]*** on this study.

I understand that I do not give up my legal rights or those of my adolescent by signing this form.

I understand that I will receive a signed and dated copy of this Participant Information and Consent Form.

If you have either read or have heard the information in this Participant Information and Consent Form, if all your questions have been answered, and if you agree that your adolescent takes part in the study, please print and sign your name and write the date on the line below.

____

Adolescent's Name (print)

______________________________ ______________________________

Parent/Guardian Name (print) **Parent/Guardian signature/Thumbprint**

Date: ______________________________

I certify that the information provided was given in a language that was understandable to the participant.

______________________________ ______________________________

Name of Study Staff
Conducting Consent Discussion (print) **Study Staff Signature**

Date: ______________________________

______________________________ ______________________________

Witness' Name (print)
(As appropriate) Date **Witness' Signature/Thumbprint**

Date: ______________________________

FIX BARCODE HERE

STUDY COMMUNITY: ______________________

APPENDIX III - SAMPLE INFORMED CONSENT FORM – QUALITATIVE STUDIES PARTICIPANTS

Tuberculosis Reduction through expanded antiretroviral therapy and TB screening (TREATS)

In-depth Interview (IDI) Information and Consent Form

Participant Information Sheet
Please ask the study investigator or the study staff to explain any words or procedures that you do not clearly understand.

This form gives you information about the research study you are being asked to join. The form describes the purpose, procedures, benefits, and risks of the research study. Please read this Information and Consent Form and ask as many questions as needed. You should not sign this form if you have any questions that have not been answered to your satisfaction. If you choose to sign this form you are giving permission to be included in this research study.

This study is being funded by the European and Developing Countries Clinical Trials Partnership (EDCTP)

Purpose of the Study
The TREATS study is made up of 4 studies that will look whether the PopART intervention that has been carried out in some communities, reduced the chance of getting TB infection or developing TB disease. The PopART intervention involved HIV testing and treatment as well as screening for the symptoms that suggest you might have TB. In total fourteen (14) communities are included in this research, 8 in Zambia and 6 in South Africa.

You are being asked to participate in the **qualitative research activities which aim to understand how TB is experienced and managed by community members and local stakeholders.**

Description of the study
This research is being carried out in your community because your community participated in the PopART study. Some members of the community – like yourself - will be asked to participate and be interviewed by the study social science's team.

Your involvement
You are being asked to participate in one of the qualitative studies as someone who can contribute to our understanding of TB in the community. You are being approached because you are either a TB stakeholder, a TB health facility staff member, or a TB patient diagnosed either through community HIV care providers (CHiPs) or through the local health facilities.

You are being asked to participate in an interview with a study staff member who will ask you questions about your life and your own experience of TB in this community. Each interview is expected to last about 60-90 minutes. The staff member will take notes, and, with your permission, the interview will be recorded. We really value the information and time you share with us.

Confidentiality
We will do everything possible to protect your confidentiality if you join this study. We do this by giving you a study number (for example, 1234782) and any information will be labelled with this number. Your name and any other information that may identify you or your household will be kept confidential and only the research staff will be able to link this number to your name. Although we will record with your permission the interview, information will not be linked or traced back to you. When the interview is fully transcribed (written up), the transcription will not bear actual names of informants and recordings will be destroyed. The results of this research may be published and full quotes from individuals may be used, but your identity and confidentiality will be protected because the quotes will not be linked to named individuals. However, if we identify any serious health or welfare problems during the course of this research, we are obligated to refer to others who can help.

You have the right to control the use and disclosure of your personal information. People who may review your records include **[*insert any site-specific institutions, national regulatory authorities and local research committees*]**. All personnel accessing your records are required to respect your confidentiality at all times. All records identifying you will be kept confidential, and to the extent permitted by applicable laws and regulations, will not be made publicly available. No personal information will be included in the study data that will be forwarded to the sponsor or sponsor representatives. You will be identified by a coded number in any reports of publications produced from this study (study data). By signing this document, you are authorizing such access.

To protect your privacy, you will meet with the researcher in a private area.

[*Insert any other site-specific regulations that need to be included in this section*].

Data Protection

What kind of information will be collected from you?
No one will be able be to recognise you in all of the data that will be collected. A barcode ID with your study number will be allocated to you and will be used instead of your name.

[Insert any other site-specific regulations that need to be included in this section, i.e. refer to national Protection of Personal Information Act].

How will data be recorded?
Some of the information that you give us will be recorded on paper for example the assent form that you will sign. All this information will be assigned a barcode ID so that your confidentiality is maintained. We will record the IDI and we will take notes on it. Notes and recordings will be then uploaded into a centrally managed system at head office into a data protected folder which is password protected and has limited access. Recordings will be transcribed and destroyed afterwards. The transcription will be cleaned of any identifiers. We will then upload these transcriptions into a qualitative data software for analysis and coding.

How will it be stored?
All paper copies that will have your information will be kept securely in a locked cabinet in a locked room that will only be accessed by assigned study staff. All electronic data will stored

on a server and will be encrypted and password protected and will only be accessible by the data manager.

All the information collected will be stored for approximately 7 years after the study has ended after which, data will be destroyed.

<u>Who will the information be shared with?</u>
We may share it with people who check that the study is done properly (like the independent ethics committee or review boards). The data that we collect, but not your name or anything else that can identify you will be shared with other researchers working on the TREATS study. These include researchers working at Zambart in Zambia, Health Systems Trust in South Africa, the London School of Hygiene and Tropical Medicine, Imperial College and the University of Sheffield in the UK and KNCV in the Netherlands. We will publish study results in medical journals, for meetings and on the internet for other researchers to use. Your name or personal information will not appear in any publication.

After the study is complete copies of the data, without any details that could identify you, will be made publicly available via the internet for other researchers to use. To make sure you can never be identified we will remove information such as your name, where you live, your date of birth, the name of your community and any other data that may lead to someone being able to identify you.

Voluntary participation and withdrawal
Your participation in the research is voluntary. If you feel uncomfortable about any questions we ask, please feel free not to answer them. If you no longer wish to participate in this interview, you may do so with no penalty.

Alternatives to participation
The alternative to participating in the interview is not to participate, which, as noted, will not result in any penalty or loss of benefits you normally receive.

Risks and benefits
There is a chance that some of our questions may cause discomfort or emotional stress. If so, you are not obligated to answer them. There are no direct benefits associated with participation in these individual interviews, but there may be indirect benefits for your community in the future. The information gained in this study may help organizations design future HIV prevention interventions.

Compensation
You will be interviewed at a time and location convenient to you because we know your time is valuable. This could be at your home or place of work (lunch time). All qualitative activities will last about one hour and thirty minutes. During this time, we will provide a snack refreshment. [***Insert if participants will be given any compensation or will be refunded for transport costs, include amount***]

Reasons for stopping participation

You may be withdrawn from the study if the research study, or this part of the study, is stopped or cancelled. You may also be withdrawn if the study if staff feels that completing the study or this part of the study would be harmful to you or others.

Contacts for questions

If you have any questions about your participation in this research study, your rights as a research subject, or if you feel that you have experienced a research-related injury, contact:

Principle investigator's Name: **[Insert name PI]**

Research Site Address (es***): [Insert PI's address]***

Daytime telephone number (s): ***[Insert PI's telephone number]***

If you have any other questions or concerns about your rights as a research participant or want to discuss a problem, get information or offer input, you may contact:

Independent Review Board/Ethics Committee: ***[Insert IRB and/or Ethics Committee]***

Address of Independent Review Board: ***[Insert IRB and/or Ethics Committee's address]***

Daytime Telephone Number: ***[Insert IRB and/or Ethics Committee's telephone number]***

[Insert any other required authorities to be contacted for each site]

Tuberculosis Reduction through expanded antiretroviral therapy and TB screening (TREATS)

In-depth Interview

STATEMENT OF CONSENT

I have been given sufficient time to consider whether to take part in this study.

I have read the Information Sheet carefully, and has been explained to me to my satisfaction

I understand my role in the interview and why the discussion is being recorded on a voice recorder.

I understand that my participation in this research is entirely voluntary, i.e. I do not have to participate if I do not wish to

I have been informed that refusal to take part will involve no penalty or loss of services to which I am otherwise entitled.

I understand that if I decide to take part, I am free to withdraw at any time without penalty or loss of services and without giving a reason for my withdrawal.

I have been informed that I may choose not to answer particular questions that are asked in the study and that if there is anything that I would prefer not to discuss, I am free to say so

I have been told that the information collected in this interview will be kept strictly confidential.

I understand that what I tell in the interview can be written up word for word (directly quoted) in any publications or reports, but that these quotations will NOT be linked to me personally.

I understand that if I choose to participate in this interview the signed consent is required below before I proceed with the interviews.

VOLUNTARY CONSENT

I have read (or have had explained to me) the information about this research as contained in the Participant Information Sheet. I have had the opportunity to ask questions about it and any questions I have asked have been answered to my satisfaction.

I now consent voluntarily to be a participant in this study and understand that I have the right to withdraw at any time, and to choose not to answer particular questions that are asked in the course of the interviews.

I voluntarily agree to take part in this research study.

__________________________________ __________________________________

Participant's Name (print) **Participant's Signature/Thumbprint**

Date: __________________________________

I certify that the information provided was given in a language that was understandable to the participant.

__________________________________ __________________________________

Name of Study Staff
Conducting Consent Discussion (print) **Study Staff Signature**

Date: __________________________________

__________________________________ __________________________________

Witness' Name (print)
(As appropriate) Date **Witness' Signature/Thumbprint**

Date: __________________________________

FIX BARCODE HERE

STUDY COMMUNITY: ________________________

Tuberculosis Reduction through expanded antiretroviral therapy and TB screening (TREATS)

Focus Group Discussion (FGD) Information and Consent Form

Participant Information Sheet

Please ask the study investigator or the study staff to explain any words or procedures that you do not clearly understand.

This form gives you information about the research study you are being asked to join. The form describes the purpose, procedures, benefits, and risks of the research study. Please read this Information and Consent Form and ask as many questions as needed. You should not sign this form if you have any questions that have not been answered to your satisfaction. If you choose to sign this form you are giving permission to be included in this research study.

This study is being funded by the European and Developing Countries Clinical Trials Partnership (EDCTP)

Purpose of the Study

The TREATS study is made up of 4 studies that will look whether the PopART intervention that has been carried out in some communities, reduced the chance of getting TB infection or developing TB disease. The PopART intervention involved HIV testing and treatment as well as screening for the symptoms that suggest you might have TB. In total fourteen (14) communities are included in this research, 8 in Zambia and 6 in South Africa.

You are being asked to participate in the **qualitative research activities which aim to understand how TB is experienced and managed by community members and local stakeholders.**

Description of the study

This research is being carried out in your community because was a community participating in the PopART study. Some members of the community – like yourself - will be asked to participate and be interviewed by the study social science's team.

Your involvement

You are being asked to participate in one of the qualitative studies as someone who can contribute to our understanding of TB in the community. You are being approached because you are a community HIV care provider (CHiP) from the PopART study who carried out TB screening and testing in households.

You are being asked to participate in a focus group discussion with other CHiPs and a study staff member who will ask you questions about your job as a CHiP and your own experience of TB in this community. Each focus group discussion is expected to last about 60-90 minutes. The staff member will take notes, and, with your permission, the interview will be recorded. We really value the information and time you share with us.

Confidentiality
We will do everything possible to protect your confidentiality if you join this study. We do this by giving you a study number (for example, 1234782) and any information will be labelled with this number. Your name and any other information that may identify you or your household will be kept confidential and only the research staff will be able to link this number to your name. Although we will record with your permission the interview, information will not be linked or traced back to you. When the interview is fully transcribed (written up), the transcription will not bear actual names of informants and recordings will be destroyed. The results of this research may be published and full quotes from individuals may be used, but your identity and confidentiality will be protected because the quotes will not be linked to named individuals. However, if we identify any serious health or welfare problems during the course of this research, we are obligated to refer to others who can help.

You have the right to control the use and disclosure of your personal information. People who may review your records include ***[insert any site-specific institutions, national regulatory authorities and local research committees]***. All personnel accessing your records are required to respect your confidentiality at all times. All records identifying you will be kept confidential, and to the extent permitted by applicable laws and regulations, will not be made publicly available. No personal information will be included in the study data that will be forwarded to the sponsor or sponsor representatives. You will be identified by a coded number in any reports of publications produced from this study (study data). By signing this document, you are authorizing such access.

To protect your privacy, you will meet with the researcher in a private area.

[Insert any other site-specific regulations that need to be included in this section].

Data Protection

What kind of information will be collected from you?
No one will be able be to recognise you in all of the data that will be collected. A barcode ID with your study number will be allocated to you and will be used instead of your name.

[Insert any other site-specific regulations that need to be included in this section, i.e. refer *to national Protection of Personal Information Act].*

How will data be recorded?
Some of the information that you give us will be recorded on paper for example the assent form that you will sign. All this information will be assigned a barcode ID so that your confidentiality is maintained. We will record the FGD and we will take notes on it. Notes and recordings will be then uploaded into a centrally managed system at head office into a data protected folder which is password protected and has limited access. Recordings will be transcribed and destroyed afterwards. The transcription will be cleaned of any identifiers. We will then upload these transcriptions into a qualitative data software for analysis and coding.

How will it be stored?
All paper copies that will have your information will be kept securely in a locked cabinet in a locked room that will only be accessed by assigned study staff. All electronic data will stored

on a server and will be encrypted and password protected and will only be accessible by the data manager.

All the information collected will be stored for approximately 7 years after the study has ended after which, data will be destroyed.

Who will the information be shared with?
We may share it with people who check that the study is done properly (like the independent ethics committee or review boards). The data that we collect, but not your name or anything else that can identify you will be shared with other researchers working on the TREATS study. These include researchers working at Zambart in Zambia, Health Systems Trust in South Africa, the London School of Hygiene and Tropical Medicine, Imperial College and the University of Sheffield in the UK and KNCV in the Netherlands. We will publish study results in medical journals, for meetings and on the internet for other researchers to use. Your name or personal information will not appear in any publication.

After the study is complete copies of the data, without any details that could identify you, will be made publicly available via the internet for other researchers to use. To make sure you can never be identified we will remove information such as your name, where you live, your date of birth, the name of your community and any other data that may lead to someone being able to identify you.

Voluntary participation and withdrawal
Your participation in the research is voluntary. If you feel uncomfortable about any questions we ask, please feel free not to answer them. If you no longer wish to participate in this interview, you may do so with no penalty.

Alternatives to participation
The alternative to participating in the interview is not to participate, which, as noted, will not result in any penalty or loss of benefits you normally receive.

Risks and benefits
There is a chance that some of our questions may cause discomfort or emotional stress. If so, you are not obligated to answer them. There are no direct benefits associated with participation in these individual interviews, but there may be indirect benefits for your community in the future. The information gained in this study may help organizations design future HIV prevention interventions.

Compensation
You will be interviewed at a time and location convenient to you because we know your time is valuable. This could be at your home or place of work (lunch time). All qualitative activities will last about one hour and thirty minutes. During this time, we will provide a snack refreshment. ***[Insert if participants will be given any compensation or will be refunded for transport costs, include amount].***

Reasons for stopping participation
You may be withdrawn from the study if the research study, or this part of the study, is stopped or canceled. You may also be withdrawn if the study staff feels that completing the study or this part of the study would be harmful to you or others.

<u>Contacts for questions</u>

If you have any questions about your participation in this research study, your rights as a research subject, or if you feel that you have experienced a research-related injury, contact:

Principle investigator's Name: **[Insert name PI]**

Research Site Address (es***): [Insert PI's address]***

Daytime telephone number (s): ***[Insert PI's telephone number]***

If you have any other questions or concerns about your rights as a research participant or want to discuss a problem, get information or offer input, you may contact:

Independent Review Board/Ethics Committee: ***[Insert IRB and/or Ethics Committee]***

Address of Independent Review Board: ***[Insert IRB and/or Ethics Committee's address]***

Daytime Telephone Number: ***[Insert IRB and/or Ethics Committee's telephone number]***

[Insert any other required authorities to be contacted for each site]

Tuberculosis Reduction through expanded antiretroviral therapy and TB screening (TREATS)

Focus Group Discussion

STATEMENT OF CONSENT

I have been given sufficient time to consider whether to take part in this study.

I have read the Information Sheet carefully and it has been explained tome to my satisfaction

I understand why I have been approached for a focus group discussion

I understand that my participation in this research is entirely voluntary, i.e. I do not have to participate if I do not wish to

I have been informed that that refusal to take part will involve no penalty or loss of services to which I am otherwise entitled

I understand that if I decide to take part, I am still free to withdraw at any time without penalty or loss of services and without giving a reason for my withdrawal.

I have been informed that I may choose not to answer particular questions that are asked in the study, and that if there is anything that I would prefer not to discuss, I am free to say so.

I understand that the information collected in this discussion will be kept strictly confidential

I understand that what I tell in the interview can be written up word for word (directly quoted) in any publications or reports, but that these quotations will NOT be linked to your personally.

I understand that if I choose to participate in this discussion, my signed consent is required below before I proceed with the discussion

VOLUNTARY CONSENT

I have read (or have had explained to me) the information about this research as contained in the Participant Information Sheet. I have had the opportunity to ask questions about it and any questions I have asked have been answered to my satisfaction.

I now consent voluntarily to be a participant in this study and understand that I have the right to withdraw at any time, and to choose not to answer particular questions that are asked in the course of the interviews.

I voluntarily agree to take part in this research study.

______________________________	______________________________
**Participant's Name (print)**	**Participant's Signature/Thumbprint**

Date: ______________________________

I certify that the information provided was given in a language that was understandable to the participant.

______________________________	______________________________
**Name of Study Staff Conducting Consent Discussion (print)**	**Study Staff Signature**

Date: ______________________________

______________________________	______________________________
**Witness' Name (print) (As appropriate) Date**	**Witness' Signature/Thumbprint**

Date: ______________________________

FIX BARCODE HERE

STUDY COMMUNITY: ______________________

TREATS protocol amendments

The final version of the TREATS protocol is v4.2 included as supplement, below modifications relevant to the TB prevalence survey part of the TREATS study are outlined.

- **Amendment_4** requested inclusion of arm B communities. Protocol v4.2 includes the accepted changes

1. See below the summary and justification of the amendment (we included other changes not related to the arm B)

Summary

4. *1. The two interventions arms of the HPTN 071 trial (arm A and arm B) have been combined and show a statistically significant reduction in HIV incidence, compared to arm C, of 20%. The TREATS study should use a combined intervention arm for some of the TREATS analyses to be able to demonstrate the additional public health benefit of TB endpoints Proposed protocol amendment for TB Prevalence Surveys: We have included Arm B sites in the TB prevalence surveys. Our proposal would be to continue with recruitment of 4000 individuals in the Arm C sites but to recruit 2000 individuals from Arm A sites and to add 2000 from Arm B sites. The overall number of participants remains the same. and sample size calculations show that this has the potential to slightly increase the power of the study. Subsequent sections of the protocol have been amended to ensure consistency with these changes. Please see new version of the protocol with tracked changes, p 12, p 13, p 14, p 18, p 19, p 21, p 22, p 23, p 24, p 25, p 26, p 31, p 39, p 40, p 43, p 46 and p 49.*

   2. Implementation Science: The analyses contained within WP5 will now include analysis of all 3 arms of the HPTN 071 trial (Arms A, B and C). This is reflected in the new version of the protocol in p 35.

   3. Additional research beneficial to HPTN 071 could be embedded within the TREATS prevalence survey, which may provide additional understanding of the dissonant results of the HPTN 071 trial and give information on the actual levels of viral suppression seen in the general population free from the "Hawthorne effect" of having been in a research cohort. This would entail including some additional questions into the questionnaire for the prevalence surveys and some additional fingerstick blood sampling from individuals living with HIV or diagnosed with HIV during the survey. Please see new version of the protocol (p 34, p 42 and p 51).

   4. TB Prevalence Survey informed consent We have also made some modifications on the TB Prevalence Survey informed consent form to make it consistent with study changes described above. We have also simplified the language and shortened it as participants have found some parts to be too lengthy and repetitive (attached).

   5. In relation to the Infection Cohort we have made minor changes in the study procedures section, in relation to HIV testing (p 29 and p 30) and we are attaching a new questionnaire for follow-up of study participants (attached).

6. Finally we wish to add a consortium member. This member is the London School of Economics (LSE). The reason for this amendment is that Dr Ranjeeta Thomas, who is the lead health economist on the TREATS consortium has left Imperial College and moved to LSE

Justification:

The TREATS study is measuring the impact of the HPTN 071 (PopART) trial on TB. From the start of the study the study decided to concentrate on just 14 of the 21 communities in HPTN 071, for logistical and financial reasons, and so elected to compare HPTN 071 Arm A (full intervention) with HPTN 071 Arm C as we believed a priori that Arm A would be more effective than Arm B, which had the same community activities for HIV and TB case finding but followed national guidelines for ART initiation. The HPTN 071 trial released its primary endpoint results in March 2019 which can be summarized as follows: a. The PopART intervention delivered with ART according to local guidelines (Arm B, universal ART from 2016), showed a statistically significant 30% reduction in HIV incidence (primary trial outcome) compared to the control arm (Arm C) b. The PopART intervention with universal ART from the start of the trial (Arm A) showed a non-significant 7% reduction in HIV incidence compared to Arm C c. Viral suppression in individuals in the population cohort was good across all 3 arms, although higher in Arm A than arm B and both higher than arm C. We are concerned that this result may be affected by the Hawthorne effect in cohort members, as individuals in the population cohort were offered rapid HIV testing at every visit and were referred to care if they were found to be HIV positive. d. Intervention data shows that coverage in arm A and arm B were good and if anything, slightly better in Arm A. Implications for the TREATS study can be summarized as follows: a. We have no evidence that the intervention, including ACF for TB, was applied any differently in the 2 intervention arms (Arm A and Arm B) b. We have no evidence that ART use was delayed in arm A or was not as effective as in Arm B, in fact viral suppression amongst the PC members was better in Arm A than in Arm B c. From a TB perspective we should see the same effect in Arm A as in Arm B d. The challenge is that since Arm A was not effective at reducing HIV incidence we will not be able to show additional benefit or cost-effectiveness of adding the TB intervention, should there in fact be one. These findings were discussed by the Project Management Group (PMG) on March 7th and by the Study Advisory Group (SAG) on March 11th and a protocol amendment was recommended. The following are the recommendations, with the changes made in the new version of the protocol,

- When **amendment 10** was requested to make changes in sample size we did not modify the protocol. We avoided this by arguing that changes only applied to some of the TBPS communities but not all, as numbers in those that have already participated were the initial ones proposed in the protocol.

Summary

In this Amendment_10 to the TREATS protocol v4.2 we propose to reduce the number of individuals enrolled in the TREATS TB Prevalence Survey (TBPS) in the 4 communities that are yet to be completed in Zambia, and the 6 communities that are yet to be completed in SA. TBPS field activities had to be interrupted for 6 months due to the COVID pandemic. Due to the tight timeline and the limited resources available for completing the TBPS, the TREATS project management group has agreed to reduce the target from 4000 to 3000 participants in Arm C communities that are yet to be completed, and from 2000 to 1500 participants in each of the Arm A and B communities that are yet to be completed. The communities that have already been completed did reach the initial targets, and sometimes exceeded them as the approved protocol indicated that the blocks in which the target was reached had to be completed. We have estimated that the original planned total sample size of 56,000 participants will be reduced to around 50,000 to 51,000 participants (based on communities that have been completed, and our projections for communities yet to be completed), i.e. a reduction of around 10%. We have estimated that study power to show PopART intervention effectiveness of a reduction in TB prevalence of 40-50% compared with standard-of-care (Arm C) communities will be reduced by around 2-3%, which is a relatively small reduction. We have not made any changes to the current version of the protocol as this amendment will only affect enrolment numbers in remaining communities while in the ones that have already participated numbers are those reflected in the protocol.

In response to the challenges and delays created by the COVID pandemic in the running of the TREATS TBPS it was agreed within the team and the project management group that the target of enrolled individuals for the TBPS would be reduced in the communities that are yet to be completed, to allow the project to be completed on time and to enable all 21 study communities to be included in the TBPS. Overall, the TREATS TBPS was planned to be conducted in 21 communities, or 7 triplets (each triplet including one Arm A, one Arm B and one Arm C community), 12 communities or 4 triplets in Zambia and 9 communities or 3 triplets in SA. Field activities had to be interrupted due to COVID in March 2020, and at that point the TREATS TBPS had already been completed in 7 communities in Zambia and in 3 communities in South Africa. When field work was resumed (August 2020 in Zambia, September 2020 in South Africa) we realised that if we wanted to complete the TBPS on time with the available resources, it was necessary to reduce the target numbers of participants needed to be enrolled in the communities that were yet to be completed (5 communities in Zambia, 2 in triplet 2 and 3 in triplet 4; 6 communities in South Africa, 3 in triplet 6 and 3 in triplet 7). The TREATS TBPS is expected to be completed in June 2021, and the whole project finishes in October 2021.
