# Supplementary material for: Tuberculosis prevalence after 4 years of population-wide systematic TB symptom screening and universal testing and treatment for HIV in the HPTN 071 (PopART) community-randomised trial in Zambia and South Africa: A cross-sectional survey (TREATS)
Source: PLoS Med. 2023 Sep 8;20(9):e1004278. doi: 10.1371/journal.pmed.1004278 (PMC10490889; doi:10.1371/journal.pmed.1004278)
Supplement: S1 SAP — (PDF) [file pmed.1004278.s016.pdf]

TREATS TB Prevalence survey Statistical Analysis Plan (SAP) – September 2021

Authors: Sian Floyd, Eveline Klinkenberg, Thomas Gachie, Richard Hayes

TREATS and HPTN 071 trial design

The TREATS study is being conducted during 2017-2021, across 21 urban and peri-urban communities in Zambia and the Western Cape of South Africa with a total population of around 1 million individuals. In the HPTN 071 (PopART) study conducted during 2013-2018, communities were randomized to one of 3 trial arms. Communities were matched into seven triplets based on geographical area and HIV prevalence, and restricted randomisation used to allocate one community to Arm A, one community to Arm B, and one community to Arm C within each triplet; so overall, 7 communities were randomised to Arm A, 7 to Arm B, and 7 to Arm C. Four triplets were in Zambia, and three triplets were in the Western Cape of South Africa.

Arms A and B received the “full” or “intermediate” PopART intervention respectively, and Arm C received standard-of-care. The PopART intervention consisted of population-level screening for TB, combined with universal testing and treatment (UTT) for HIV, and was delivered during 2014-2017 inclusive. The intervention in Arms A and B was the same, apart from the time period during which universal ART was offered at the community government clinic. In Arm A universal treatment was offered at the clinic from the start of the intervention in January 2014, while in Arm B ART was offered according to national guidelines; in Arm B, universal treatment was offered from mid-2016.

TREATS TB prevalence survey design

A co-primary endpoint of the TREATS study is the prevalence of TB disease, measured during 2019-2021, and TB prevalence is the primary endpoint of the TREATS TB prevalence survey.

This primary endpoint of TB prevalence is measured in a random sample of individuals aged ≥15 years, in each of the 21 study communities. Target sample size was initially 4000 participants in each Arm C community, and 2000 participants in each Arm A and Arm B community. From around halfway through the survey, the target sample size was reduced to 3,500 participants aged ≥15 years in Arm C communities and 1,750 participants aged ≥15 years in each Arm A and Arm B community. From early 2021, the target sample size was reduced to 3,000 participants in Arm C communities and 1,500 in each Arm A and Arm B community.

Table 1 – Target sample size in each study community

Country	Community number	Arm	Target sample size
Zambia	1	B	2,000
Zambia	2	A	2,000
Zambia	3	C	3,500
Zambia	4	C	4,000
Zambia	5	A	2,000
Zambia	6	B	2,000
Zambia	7	C	4,000
Zambia	8	A	2,000
Zambia	9	B	2,000
Zambia	10	A	1,500
Zambia	11	B	1,500

Zambia	12	C	3,000
South Africa	14	A	2,000
South Africa	15	C	4,000
South Africa	13	B	2,000
South Africa	16	A	1,750
South Africa	17	C	3,000
South Africa	18	B	1,500
South Africa	19	A	1,500
South Africa	21	C	3,000
South Africa	20	B	1,500

The main comparison of TB prevalence is between the 14 Arm A and Arm B communities (PopART intervention) compared with the 7 Arm C (standard-of-care) communities.

Random sampling of the community population was structured according to geographically-defined blocks, with each community divided into blocks of around 200 households (~500 individuals aged ≥15 years). The blocks were randomly ordered, the prevalence survey started in the block that was randomly ordered first, and then proceeded according to the random ordering up to completion of the block in which the target sample size of participants was reached, with all households in a block and all their resident members who were aged ≥15 years eligible to participate.

Prevalence survey procedures that were consistent throughout the survey

Household enumeration, and invitation to participate

For each block that was included in the prevalence survey, all households were visited by a research assistant; and, for all households where an adult household member was contacted, permission was sought to enumerate (list) all household members. In enumerated households, an individual was eligible to participate in the survey if they were ≥15 years and usually resident in the community, with "usually resident" defined as having lived in the community for ≥9 of the previous 12 months. All eligible individuals were given barcoded invitation cards, and invited to participate in the survey at a mobile field site that was set up within or near each block.

Consent to participate, and participation, at mobile field site

All individuals who attended the mobile field site and consented to participate in the survey followed a defined order of procedures. First, questionnaire information including TB symptoms, previous history of TB treatment, and previous HIV testing history, was collected. HIV testing was offered to individuals who did not self-report they were HIV-positive. A digital chest X-ray was taken in a One Stop TB clinic (*Delft Imaging, the Netherlands*), with X-ray images read using computer-aided-detection (CAD) reading (CAD4TB version 5.0) that provided a score between 0% and 100% as an indication of the probability that an individual has TB.

Sputum eligibility

Individuals who had a cough for ≥2 weeks or who had ≥2 among 5 symptoms that are suggestive of TB (cough of any duration, unexpected weight loss for ≥4 weeks, night sweats for ≥2 weeks, chest pains for ≥2 weeks, fever for ≥2 weeks), and/or a CAD X-ray score equal to or above a pre-defined threshold were "sputum-eligible". During the Intensive Diagnostic Phase (IDP), that included the first 4 communities in which the TREATS TB prevalence survey was conducted, the CAD score threshold for sputum eligibility was 40%. For the non-IDP communities the CAD score threshold was raised to

50%. Individuals were also sputum-eligible if they consented to participate in the survey, but were unable or refused to have a chest X-ray taken.

Sputum sample provision, among "sputum-eligible" individuals

"Sputum-eligible" individuals were requested to provide two "on-the-spot" sputum samples (S1 and S2) for Xpert-Ultra testing, with the two samples taken at least 30 minutes apart. Xpert-Ultra testing of these two samples was done at the mobile field site, on the same day as sample collection or on the following day.

Classification of Xpert-Ultra test results on S1 and S2 sputum samples

Xpert-Ultra test results were classified as negative, MTB-detected-trace, MTB-detected-very low, MTB-detected-low, MTB-detected-medium, MTB-detected-high, invalid, error or no result. Test results among very low, low, medium, and high were grouped as "positive", distinguished from trace-positive and from negative. For samples with invalid, error, or no result on the first test, up to 3 repeat tests were done in order to try to obtain a valid result.

Prevalence survey procedures on "Day 2", after first participation on "Day 1", Intensive Diagnostic Phase (IDP)

The "IDP" phase was for the first 4 communities that were included in the TREATS TB prevalence survey, three in triplet 1 in Zambia and one Arm A community in triplet 4 in South Africa.

Receipt of Xpert-Ultra test results, medical officer review

All individuals who were sputum-eligible were requested to return to the mobile field site the day after providing S1 and S2 (Day 1), to receive their Xpert-Ultra test results. On this "Day 2" they met a medical officer, who reviewed all available screening and test results alongside information including self-reported previous TB treatment and HIV status, re-enquired about TB symptoms, recorded their interpretation of the chest X-ray, and made decisions on referral to TB and/or other care.

Provision of S3 sample for culture testing

All sputum eligible individuals who returned for "Day 2" were asked to provide a third "on-the-spot" sputum sample (S3). S3 samples were batched and transported from the field to a laboratory in a cooler box, with each box also including a "dummy" sputum sample of known bacterial load so as to monitor the effect of transportation on sample viability. In Zambia they were taken to the Zambart laboratory in Lusaka, and in South Africa to the National-Health-Laboratory-System (NHLS) Greenpoint laboratory in Cape Town.

Prevalence survey procedures on "Day 2", non-IDP phase

As with the IDP, all sputum-eligible individuals were requested to return to the mobile field site to receive the Xpert-Ultra test results from the S1 and S2 sputum samples, and to meet a medical officer who had reviewed their screening and test results.

Eligibility to provide S3 and S4 samples for culture testing; definition of "culture-eligible" individuals, as a subset of sputum-eligible individuals

In the non-IDP phase, individuals were eligible for culture testing depending on the results of Xpert-Ultra testing on S1 and S2. Culture eligibility could only be determined if Xpert-Ultra test results were available for both S1 and S2, and if those results were among negative, MTB-detected-trace, MTB-detected-very low, MTB-detected-low, MTB-detected-medium, or MTB-detected-high – these were considered "valid" test results. For individuals whose test results on S1 and/or S2 were among invalid or error or no result, these were all considered "invalid" test results and repeat testing was done on the S1 and/or S2 samples. For individuals who did not provide both of the S1 and S2 samples on Day 1, they were asked to provide these on "Day 2".

Among individuals with (a) Xpert-Ultra test results for both S1 and S2, and (b) with results for each of S1 and S2 that were among negative, trace-positive, or positive, culture eligibility could be determined. It was determined as follows:

1. Individuals whose S1 and S2 test results were both negative were not eligible to provide sputum samples for culture testing, and they were classified as "prevalent TB=No" based on Xpert-Ultra testing.
2. Individuals whose S1 and S2 test results were both at least very low, with at least one low, were also not eligible to provide sputum samples for culture testing, and they were classified as "prevalent TB=Yes" based on Xpert-Ultra testing.
3. **Individuals whose S1 and S2 results were combinations that included at least one test result that was trace-positive or higher, but did not meet the criteria in 2., were eligible for culture testing; i.e., they are "culture-eligible" individuals. Their prevalent TB status was determined on the basis of their culture test results on S3 and/or S4.**

In the non-IDP phase, among individuals who were in category 3. above and so were "culture-eligible", they were requested to provide two sputum samples – S3 and S4 – for culture testing.

Classification of culture test results on S3 and/or S4 sputum samples

For S3 and S4 samples, upon their arrival at the central laboratory (Zambart or Greenpoint, for Zambia and South Africa respectively) they were processed according to standard MGIT culture SOPs, and each of the S3 and S4 samples was inoculated onto 2 MGIT tubes.

Tubes were incubated in batches, with each batch also including one positive control and one negative control sample, and remained incubated until the earliest of mycobacterial or other growth, or 6 weeks later. For tubes with growth, further testing was done in order to distinguish *M. tuberculosis* from non-tuberculous mycobacteria (NTM) and from contamination.

The culture test result from each MGIT tube was classified as negative, positive for *M. tuberculosis*, positive for non-tuberculous mycobacteria (NTM), non-interpretable, or contaminated. The "final" culture result for each of S3 and S4 was defined based on the combination of results from the 2 MGIT tubes. If ≥1 tube result was positive for *M. tuberculosis* then the sputum sample (S3 or S4) was classified as culture-positive. Among S3 and S4 that were not culture-positive for *M. tuberculosis*, they were classified as culture-negative if ≥1 tube was positive for NTM or there was no mycobacterial growth, and as contaminated if both tubes were contaminated.

Thus the S3 sample, and the S4 sample, were each classified as culture-positive for *M. tuberculosis*, culture-negative for *M. tuberculosis*, or culture-contaminated.

To then classify an *individual* as culture-positive or culture-negative or culture-unknown (culture-missing) status, we combined the information from the S3 and S4 samples:

- If ≥1 S3 or S4 sputum sample culture test result was positive for *M. tuberculosis*, then the individual was classified as **culture-positive**;
- Among individuals for whom neither S3 nor S4 was culture-positive for *M. tuberculosis*, they were classified as **culture-negative** if ≥1 among S3 or S4 was positive for NTM or there was no mycobacterial growth.
- An individual was classified as of **culture-unknown (and therefore missing) status** if the results from both S3 and S4 were culture-contaminated or "missing".

For individuals with culture results from S3 and/or S4, but the sample was cultured in a batch that failed the quality control check, i.e. that a positive control sample grew and a negative control sample did not grow, the culture result was set to "missing".

For individuals who did not provide S3 and/or S4 samples, or the samples were provided but not tested, the culture status for the sample(s) that were not provided or tested was also set to "missing".

Primary outcome – numerator and denominator

The denominator for analysis includes all survey participants.

The numerator counts all individuals who are classified as having prevalent TB (yes or no) on the day of survey participation.

The outcome is then the proportion of individuals who had prevalent TB on the day of survey participation.

Survey participants can be categorised into 3 groups with respect to how they count in the numerator, based on the available data:

1. Prevalent TB = No
2. Prevalent TB = Yes
3. Prevalent TB = Unknown, due to missing data at one or more steps of the diagnostic algorithm that is used to determine if an individual who screened positive on chest X-ray and/or TB symptoms, and so was sputum-eligible, has prevalent TB (yes or no).

Categorisation of individuals into groups 1-3, among individuals who were not "sputum-eligible"

All survey participants who screened negative on TB symptoms and chest X-ray, and so were not eligible to provide sputum samples S1 and S2, are categorised in group 1, i.e. Prevalent TB = No.

Categorisation of individuals into groups 1-3, among individuals who were "sputum-eligible"

Among sputum-eligible individuals, the survey protocol was that they would provide two sputum samples, S1 and S2, for Xpert-Ultra testing.

Among individuals with Xpert-Ultra test results on both S1 and S2, they were classified as prevalent TB yes, no, or unknown, as follows:

i. S1 and S2 Xpert-Ultra test results both negative: categorised as group 1, i.e. "Prevalent TB=No".

ii. S1 and S2 Xpert-Ultra test results both at least very low, with at least one low: categorised as group 2, i.e. “Prevalent TB=Yes”.
iii. S1 and S2 Xpert-Ultra test results that were combinations that included at least one test result that was trace-positive or higher, but did not meet the criteria in ii., and culture-positive: categorised as group 1 i.e. “Prevalent TB=Yes”
iv. S1 and S2 Xpert-Ultra test results that were combinations that included at least one test result that was trace-positive or higher, but did not meet the criteria in ii., and culture-negative: categorised as group 2 i.e. “Prevalent TB=No”
v. S1 and S2 Xpert-Ultra test results that were combinations that included at least one test result that was trace-positive or higher, but did not meet the criteria in ii., and culture-unknown status: categorised as group 3 i.e. “Prevalent TB=Unknown”

Among sputum-eligible individuals who were screened on both TB symptoms and with a chest X-ray, and they had Xpert-Ultra test results missing for S1 and/or S2, they were also categorised as group 3, i.e. “Prevalent TB = Unknown”.

Among individuals who were sputum-eligible because they did not have a chest X-ray done, and they had Xpert-Ultra test results missing for S1 and/or S2, they were categorised as group 1, i.e. “Prevalent TB = No”. This was on the basis of interim data (after half the survey was completed) that showed that, among individuals who did not have a chest X-ray done, there were 0 TB cases among those with both S1 and S2 Xpert-Ultra test results available, and 0 individuals with an Xpert-Ultra test result other than negative or trace-positive. For this group of individuals, classifying them as “Prevalent TB = No” is transparent and avoids potential problems with imputation for a group with a zero or very low probability of the outcome, especially in the context that this group of individuals has missing data for an important predictor (X-ray reading) of TB status.

Multiple imputation of missing data will be used to generate datasets in which the data on the outcome of prevalent TB is complete for all sputum-eligible individuals, i.e. datasets in which all sputum-eligible individuals are categorised as either Prevalent TB=No or Prevalent TB=Yes.

Imputation of missing data, over 4 steps

Step 1 – individuals with both S1 and S2 Xpert-Ultra test results, and eligible to provide S3 and S4 for culture, but of culture-unknown status

We will create a dataset that is restricted to culture-eligible individuals, with culture eligibility as defined and applied during the non-IDP phase of the prevalence survey (see, “***Eligibility to provide S3 and S4 sputum samples for culture testing”***, above).

The variables included on this dataset will be:

1. The combination of S1/S2 Xpert-Ultra test results, categorised as (1) trace-positive only (2) ≥1 of S1 or S2 grade very low or above
2. the overall culture result based on S3 and/or S4, categorised as culture-positive (coded 1) or culture-negative (coded 0), or missing data
3. Country, i.e. Zambia or South Africa
4. Triplet (1 to 7)
5. Self-reported TB treatment history, categorised as None (coded 0), 1 (previous TB treatment, but not currently on TB treatment) and 2 (currently on TB treatment)
6. HIV status: Using a combination of self-reported information and HIV test results from testing as part of the TREATS survey. Categorised as HIV-positive (self-reported HIV-positive

or tested HIV-positive in TREATS survey), HIV-negative (tested HIV-negative in TREATS survey, or self-reported testing HIV-negative in previous 12 months), or unknown.

7. TB symptoms: Cough ≥2 weeks yes or no, number of TB symptoms (categorised as 2 or more, or <2)
8. X-ray CAD score, categorised, for example as <50, 50-69, 70+

Based on interim data analysis to identify the most appropriate explanatory variables to include in the imputation model (to impute culture status, positive or negative), the imputation will be done as follows:

(a) Using a composite variable encompassing S1/S2 Xpert results, TB treatment history, and country:

1. No previous TB treatment, S1/S2 ≥1 grade very low, Zambia or South Africa
2. No previous TB treatment, S1/S2 trace only, Zambia
3. No previous TB treatment, S1/S2 trace only, South Africa
4. Previous TB treatment, S1/S2 ≥1 very low or trace only, Zambia
5. Previous TB treatment, S1/S2 ≥1 very low or trace only, South Africa
6. Currently on TB treatment

and

(b) HIV-positive (versus HIV-negative and unknown HIV status) among groups (2) to (5) (HIV-positive status does not add to the prediction in group (1) above)

We will then use multiple imputation, to impute the missing data on the overall culture result. We will repeat the multiple imputation process ≥10 times, depending on the amount of missing data on culture results. For example, if around 20% of culture results are missing, we will create 20 imputed datasets. If around 30% of culture results are missing, we will create 30 imputed datasets.

Step 2 – Creation of "complete" datasets among sputum-eligible individuals who had Xpert-Ultra test results from both S1 and S2

1. Following Step 1, we will have created 10-30 imputed datasets with complete data on prevalent TB status (yes or no) among sputum-eligible individuals who were also culture-eligible.
2. Among sputum-eligible individuals who had both S1 and S2 Xpert-Ultra test results available, and who were not culture-eligible, their prevalent TB status (yes or no) was already complete. We will create 10-30 copies of the original data on these individuals.
3. For each of the datasets in 2., we will combine them with one of the 10-30 datasets for culture-eligible individuals that were completed (for prevalent TB status yes or no) using multiple imputation in Step 1. above
4. **We will then have 10-30 datasets that include all sputum-eligible individuals who had Xpert-Ultra test results on both S1 and S2, that are complete for the outcome variable prevalent TB (yes or no).**

Step 3 – Imputation of missing data on prevalent TB status (yes or no), among sputum-eligible individuals with missing Xpert-Ultra test data on one or both of S1 and S2

1. Following Step 2, we will have created 10-30 imputed datasets with complete data on prevalent TB status (yes or no) among sputum-eligible individuals who had Xpert-Ultra test results on both S1 and S2.
2. For each of these 10-30 datasets, we will combine them with the original data on individuals who were sputum-eligible but with Xpert-Ultra test results missing on one or both of S1 and S2.
3. We will then have 10-30 datasets that include all sputum-eligible individuals, with a subset (all those with missing Xpert-Ultra test results on one or both of S1 and S2) who have missing data on the outcome variable of prevalent TB (yes or no) and on one or both of the Xpert-Ultra test results on S1 and S2.

For each of these 10-30 datasets, we will then use multiple imputation to impute the missing data on the outcome variable of prevalent TB (yes or no). We will repeat the multiple imputation process 10-20 times, depending on the amount of missing data on S1/S2 Xpert-Ultra test results (the proportion of sputum-eligible individuals with missing data on one or both of the S1 and S2 Xpert-Ultra test results). For example, if around 10% of S1/S2 Xpert-Ultra results are missing, we will create 10 imputed datasets for each of the 10-30 datasets created following Step 2. If around 20% of S1/S2 Xpert-Ultra results are missing, we will create 20 imputed datasets for each of the 10-30 datasets created following Step 2. Following this, we will have in the range 100-600 imputed datasets for sputum-eligible individuals.

Explanatory variables in the logistic regression model with the outcome of prevalent TB (yes or no), will be (in order of priority to include, with the number of explanatory variables included depending on the number of diagnosed prevalent TB cases):

- Community (to respect the community-randomised design)
- Self-reported TB treatment history, categorised as None (coded 0), 1 (previous TB treatment, but not currently on TB treatment) and 2 (currently on TB treatment)
- X-ray CAD score, categorised, for example as <50, 50-69, 70-84, ≥85
- TB symptoms: Categorised as eligible to give sputum samples based on self-reported TB symptoms, yes or no
- HIV status. Categorised as (1) self-reported HIV-positive and self-reported on ART (2) self-reported HIV-positive and self-reported not on ART (3) newly diagnosed HIV-positive (did not self-report as HIV-positive, and tested HIV-positive in TREATS survey) (4) HIV-negative (tested HIV-negative in TREATS survey, or self-reported testing HIV-negative in previous 12 months) (5) unknown HIV status
- Sex
- Age group (categorised as 15-24, 25-34, 35-44, 45-54, 55+ years)
- Interaction terms for which there is statistical evidence they improve the prediction, or there is a priori knowledge, will be included (e.g. allowing the pattern of TB prevalence with age to be different for males and females; allowing the association between chest X-ray CAD score and prevalent TB to be different for individuals with and without a previous history of TB treatment)

Based on interim data analysis to identify the most appropriate explanatory variables to include in the imputation model for prevalent TB (yes or no), the imputation will be done using the following explanatory variables:

1. Composite variable of all combinations of X-ray score in categories (<50, 50-69, 70-84, 85+) and previous TB treatment (but not currently on TB treatment) and no previous TB treatment; this gives 8 categories of individual. A ninth category is individuals who self-reported they were currently on TB treatment. [X-ray score is a strong predictor of prevalent TB status yes/no, but the association between X-ray score and prevalent TB status yes/no is stronger for those without previous TB treatment.]
2. Eligible to give sputum samples based on self-reported TB symptoms, yes or no, for individuals with an X-ray score of 50 or above and not currently on TB treatment [individuals who were eligible to give sputum samples but they had an X-ray score of <50 must have been eligible on the basis of the TB symptoms they reported]. Assuming the odds ratio comparing those who were not eligible to give sputum samples based on TB symptoms, to those who were eligible to give sputum samples based on TB symptoms, is the same across categories of X-ray score and previous TB treatment (there was no evidence against this assumption in the data)
3. Age in 5 categories (15-24, 25-34, 35-44, 45-54, 55+), and allowing the pattern (of prevalent TB) with age group to be different for males and females
4. HIV and ART status (as defined above)
5. Community

We will conduct the multiple imputation for Step 3 separately for each combination of country and trial arm (Arms A and B grouped together, Arm C). This is so as to respect the community-randomised study design, but grouping Arm A and Arm B for the purpose of imputation because the primary comparison as set out in the prevalence survey protocol is between Arms A and B combined (PopART intervention) vs Arm C (standard-of-care).

Step 4 – Creation of "complete" datasets among survey participants

For each of the 100-600 imputed datasets on sputum-eligible individuals that will have been created following Step 3, we will append them to the original data on individuals who were not sputum-eligible. From this, we will have 100-600 imputed datasets that include all survey participants and which have complete data on the outcome of prevalent TB (yes or no).

Trial arm comparison

For each of the 21 communities, the prevalence of TB disease is calculated as the number of prevalent TB cases divided by the number of survey participants. As an overall measure of the prevalence of TB in each trial arm, the geometric mean of the 7 community-level values of TB prevalence will be calculated for each trial arm, as well as a geometric mean across the 14 PopART intervention communities in Arm A and Arm B. We will also calculate the arithmetic mean of the community-level values for each trial arm, as well as across the 14 Arm A and Arm B communities.

Due to the need for imputation of missing data (see above), these prevalence values will be calculated for each imputed dataset, and then the average of their values across the imputed datasets will be used as a summary measure.

For each triplet, the prevalence ratio (PR) comparing Arm A with Arm C will be calculated as [prevalence of TB in Arm A / prevalence of TB in Arm C], separately for each of the 7 triplets. The geometric mean of the triplet-specific prevalence ratios will be used to provide an overall comparison of Arm A with Arm C.

For each triplet, the prevalence ratio (PR) comparing Arm B with Arm C will be calculated as [prevalence of TB in Arm B / prevalence of TB in Arm C], separately for each of the 7 triplets. The geometric mean of the triplet-specific prevalence ratios will be used to provide an overall comparison of Arm B with Arm C.

An overall comparison of Arms A and B combined, with Arm C, will be made by calculating the geometric mean of the 14 triplet-specific comparisons of Arm A with Arm C, and Arm B with Arm C.

Due to the need for imputation of missing data, these prevalence ratios will be calculated for each imputed dataset, and then the average of their values across the imputed datasets will be used as a summary measure of the comparison among trial arms.

The null hypothesis is there is no difference in the prevalence of TB between the trial arms that received the PopART intervention (Arm A and Arm B) and the standard-of-care arm (Arm C) (prevalence ratio (PR) = 1).

Formal comparison of trial arms

Unadjusted analysis

To formally compare the trial arms, for each imputed dataset we will analyse the 21 community-level values of TB prevalence, using the log(community-level prevalence).

We will fit a linear regression model of log(community-level prevalence) on triplet and trial arm, in order to obtain a log(prevalence ratio) comparing Arm A with Arm C, and a log(prevalence ratio) comparing Arm B with Arm C, that respects the matched (into triplets) trial design. The standard error of the log(prevalence ratio) comparing Arm A with Arm C, and Arm B with Arm C, is calculated using the residual mean square from the linear regression model, with 12 degrees of freedom (21 communities – 2 (3 trial arms) – 6 (7 triplets) – 1). The standard error for the log(prevalence ratio) comparing Arm A with Arm C, and Arm B with Arm C, within each imputed dataset, is calculated as sqrt $(2 \times s^2/7)$, where s^2 is the residual mean square from the linear regression model.

To compare the combination of Arm A and Arm B, with Arm C, the average of the two values (a) log(prevalence ratio) comparing Arm A with Arm C and (b) log(prevalence ratio) comparing Arm B with Arm C, is calculated. The standard error for comparing the combined log(prevalence ratio) for Arm A and Arm B combined with Arm C = sqrt $(s^2/14 + s^2/7)$.

The above summaries will be calculated for each imputed dataset, and then combined across imputed datasets using Rubin's rules to account for the additional uncertainty introduced due to multiple imputation.

Adjusted analysis

We will follow the 2-stage procedure for cluster-randomised trials with <30 clusters.

We will conduct Stage 1 and Stage 2 for each of the 100-600 imputed datasets. We will combine the findings from Stage 2 (21 community-level ratio-residuals, for each imputed dataset) across the 100-600 imputed datasets, using Rubin's rules to account for the additional uncertainty that is introduced due to imputation of missing data.

In Stage 1, for each imputed dataset a logistic regression model is fitted to the individual-level data. This logistic regression model will include triplet (to respect the matched trial design), and other individual characteristics that are established determinants of prevalent TB. These individual characteristics will be sex, age group, and HIV (positive or negative) status. The Stage 1 model will be

fitted separately to (1) Zambian communities and (2) South African communities; this will be consistent with the approach to imputing prevalent TB yes/no (see above), with the approach to analysis of prevalent TB yes/no in the ZAMSTAR trial, and with prior knowledge that the patterns of TB prevalence by age, sex, and HIV status may differ between Zambian and South African communities.

In a sensitivity analysis, we will also adjust for community-level HIV prevalence, given that HIV is a strong risk factor for TB disease and that community-level HIV prevalence was adjusted for in the analysis of the primary endpoint (HIV incidence) of the HPTN 071 (PopART) trial. Community-level HIV prevalence will be estimated from the HPTN 071 (PopART) Population Cohort (PC) Study enrolment data (PC0). These community-level HIV prevalence estimates will be age-sex standardized (separately for each of the 7 triplets of communities) using data collected as part of the third round of delivering the PopART intervention in 2017. For each community, these intervention data include a listing of all household members in the community and their age (in years) and sex, among households that consented to enumeration.

At Stage 1, trial arm is not included as an explanatory variable in the regression model.

We will use this logistic regression model to predict each individual's probability of having prevalent TB, under the null hypothesis of no intervention effect on TB prevalence. We will aggregate these individual-level probabilities by community, to provide an expected number of prevalent TB cases for each community. Then for each of the 21 communities we will have an observed number of individuals with prevalent TB, and an expected number of individuals with prevalent TB under the null hypothesis.

In Stage 2, for each community we will calculate the ratio of observed (O) to expected (E) individuals with prevalent TB (O/E), and then calculate the log(ratio-residual) as log(O/E). To formally compare Arm A with Arm C, Arm B with Arm C, and the combination of Arms A and B with Arm C, we will analyse the 21 community-level values of log(O/E). We will fit a linear regression model of log(O/E) on triplet and trial arm, as described above for unadjusted analysis. Estimation of prevalence ratios is done in exactly the same way as with the linear regression of log(prevalence) that is used for unadjusted analysis, but now using a linear regression of log(O/E) instead of log(prevalence).

As with unadjusted analysis, the summaries will be calculated for each imputed dataset, and then combined across imputed datasets using Rubin's rules to account for the additional uncertainty introduced due to multiple imputation. The only difference, for the adjusted analysis, is that the degrees of freedom for the residual mean square will be 11, rather than 12, in a sensitivity analysis in which community-level HIV prevalence is adjusted for at Stage 1.

Sub-group analyses

We will repeat the analyses for sub-groups defined as follows:

(1) Sex – i.e. separate analysis for males and females

(2) Age group – separate analysis for younger (age <30 years) and older (age ≥30 years)

(3) HIV status – separate analysis for HIV-negative and HIV-positive individuals

(4) Residency during the PopART intervention period 2014-2017 – analysis restricted to individuals who were resident for at least one year of the PopART intervention delivery period. We will define this as individuals who reported (in the TREATS TB prevalence survey) that they had been resident in the study community for ≥5 years.

Sensitivity analyses

We will conduct the following sensitivity analyses:

(1) For sputum-eligible individuals who were given sputum containers for S1 and S2 samples, but they were unable to provide a sputum sample for either S1 or S2 (reason given for no sputum sample = could not produce sputum), they will be classified as Prevalent TB = No, rather than having their prevalent TB status imputed. Alongside this, for sputum-eligible individuals who were given a sputum container for S1, but they were not given a sputum container for S2, and they were unable to provide sputum for the S1 sample (reason given for no sputum sample = could not produce sputum), they will be classified as Prevalent TB = No. This is with the assumption that if an individual is unable to produce a sputum sample, they are unlikely to have TB.

(2) For sputum-eligible individuals who were given sputum containers for S1 and S2 samples, but they did not provide a sputum sample for either S1 or S2, they will be classified as Prevalent TB = No regardless of the reason for not providing sputum samples, rather than having their prevalent TB status imputed. Alongside this, for sputum-eligible individuals who were given a sputum container for S1, but they were not given a sputum container for S2, and they did not provide a sputum sample for S1, they will be classified as Prevalent TB = No regardless of the reason for not providing a sputum sample for S1. This is with the assumption that if an individual is unable to produce a sputum sample, they are unlikely to have TB.
